# Evolution of *Helicobacter*: Acquisition by Gastric Species of Two Histidine-Rich Proteins Essential for Colonization

**DOI:** 10.1371/journal.ppat.1005312

**Published:** 2015-12-07

**Authors:** Daniel Vinella, Frédéric Fischer, Egor Vorontsov, Julien Gallaud, Christian Malosse, Valérie Michel, Christine Cavazza, Marie Robbe-Saule, Pierre Richaud, Julia Chamot-Rooke, Céline Brochier-Armanet, Hilde De Reuse

**Affiliations:** 1 Institut Pasteur, Département de Microbiologie, Unité Pathogenèse de *Helicobacter*, ERL CNRS 3526, Paris, France; 2 Institut Pasteur, Département de Biologie Structurale et Chimie, Unité Spectrométrie de Masse Structurale et Protéomique, CNRS UMR 3528, Paris, France; 3 Université Paris Diderot, Sorbonne Paris Cité, Cellule Pasteur, Paris, France; 4 iRTSV/LCBM CEA, Grenoble, France; 5 CEA, DSV, IBEB, SBVME and CNRS, UMR 7265 Biol Veget & Microbiol Environ, Saint-Paul-lez-Durance, France and Aix Marseille Université, BVME UMR7265, Marseille, France; 6 Université de Lyon, Université Lyon 1, CNRS, UMR5558, Laboratoire de Biométrie et Biologie Evolutive, Villeurbanne, France; University of California Davis School of Medicine, UNITED STATES

## Abstract

Metal acquisition and intracellular trafficking are crucial for all cells and metal ions have been recognized as virulence determinants in bacterial pathogens. Virulence of the human gastric pathogen *Helicobacter pylori* is dependent on nickel, cofactor of two enzymes essential for *in vivo* colonization, urease and [NiFe] hydrogenase. We found that two small paralogous nickel-binding proteins with high content in Histidine (Hpn and Hpn-2) play a central role in maintaining non-toxic intracellular nickel content and in controlling its intracellular trafficking. Measurements of metal resistance, intracellular nickel contents, urease activities and interactomic analysis were performed. We observed that Hpn acts as a nickel-sequestration protein, while Hpn-2 is not. *In vivo*, Hpn and Hpn-2 form homo-multimers, interact with each other, Hpn interacts with the UreA urease subunit while Hpn and Hpn-2 interact with the HypAB hydrogenase maturation proteins. In addition, Hpn-2 is directly or indirectly restricting urease activity while Hpn is required for full urease activation. Based on these data, we present a model where Hpn and Hpn-2 participate in a common pathway of controlled nickel transfer to urease. Using bioinformatics and top-down proteomics to identify the predicted proteins, we established that Hpn-2 is only expressed by *H*. *pylori* and its closely related species *Helicobacter acinonychis*. Hpn was detected in every gastric *Helicobacter* species tested and is absent from the enterohepatic *Helicobacter* species. Our phylogenomic analysis revealed that Hpn acquisition was concomitant with the specialization of *Helicobacter* to colonization of the gastric environment and the duplication at the origin of *hpn-2* occurred in the common ancestor of *H*. *pylori* and *H*. *acinonychis*. Finally, Hpn and Hpn-2 were found to be required for colonization of the mouse model by *H*. *pylori*. Our data show that during evolution of the *Helicobacter* genus, acquisition of Hpn and Hpn-2 by gastric *Helicobacter* species constituted a decisive evolutionary event to allow *Helicobacter* to colonize the hostile gastric environment, in which no other bacteria persistently thrives. This acquisition was key for the emergence of one of the most successful bacterial pathogens, *H*. *pylori*.

## Author Summary

*Helicobacter pylori* is a bacterium that persistently colonizes the stomach of half of the human population. Infection by *H*. *pylori* is associated with gastritis, peptic ulcer disease and adenocarcinoma. To resist gastric acidity and proliferate in the stomach, *H*. *pylori* relies on urease, an enzyme that contains a nickel-metallocenter at its active site. Thus, nickel is a virulence determinant for *H*. *pylori*. Our aim is to characterize how *H*. *pylori* controls the intracellular nickel concentration to avoid toxicity, which protein partners are involved, and how they impact urease activity and virulence. We characterized two *H*. *pylori* proteins, Hpn and Hpn-2 that are rich in Histidine residues. We demonstrated that Hpn is involved in nickel sequestration, that the two proteins interact with each other and that their combined activities participate in a nickel transfer pathway to urease. Hpn is only expressed in gastric *Helicobacter* species able to colonize the stomach and Hpn-2 is restricted to the *H*. *pylori* and its close relative *H*. *acinonychis*. We found that both proteins are essential for colonization of a mouse model by *H*. *pylori*. We conclude that during evolution, the acquisition of Hpn and Hpn-2 by gastric *Helicobacter* species was decisive for their capacity to colonize the stomach.

## Introduction

*Helicobacter pylori* is a gram-negative bacterium that colonizes the stomach of about half of the human population. Infection by this pathogen causes the development of gastro-duodenal ulcers, MALT lymphoma and gastric carcinoma [1,2] and can lead to gastric cancer, which is responsible for about 800,000 deaths worldwide every year [3]. Virulence of *H*. *pylori* directly depends on its capacity to persistently colonize the stomach, a hostile and acidic niche. Survival under such conditions relies on the activity of the nickel-containing urease, an enzyme that catalyzes hydrolysis of urea into ammonia and bicarbonate, two buffering compounds that allow the bacterium to maintain its cytoplasmic pH close to neutrality [4]. The only other nickel-dependent enzyme in *H*. *pylori*, the [NiFe] hydrogenase [5], has also been shown to be important for colonization, presumably because it provides an alternative respiratory pathway, allowing *H*. *pylori* to use molecular hydrogen as an energy source [6]. In this regard, the transition metal ion Ni(II), which is an essential constituent of the active site of urease and [NiFe]-hydrogenase, can be considered as an essential determinant for the virulence of *H*. *pylori* and for *in vivo* colonization [6,7].

Urease is very abundant in *H*. *pylori* accounting for 10% of total soluble proteins and its maturation requires four accessory proteins for nickel delivery into the active site (UreE-F-G-H) [8]. Under neutral pH and/or low nickel availability conditions, only a fraction of the urease pool is found in a nickel-loaded form. Upon exposure to acidic and/or nickel-replete conditions, the remaining pool of urease becomes activated by the nickel maturation machinery allowing a rapid adaptation to pH variations [9]. Urease requires up to 24 nickel ions per fully active enzymatic complex [10], while [NiFe] hydrogenase depends on a binuclear [NiFe] center, coordinated by CO and CN^-^ ligands.

*H*. *pylori* therefore needs to acquire large amounts of nickel in order to maturate and activate these two essential enzymes and consequently to survive within the stomach (for a review, see [11]). Accordingly, nickel intracellular concentration of *H*. *pylori* cells is about 50-fold higher than in *Escherichia coli* [12]. The nickel concentration measured in the human body is very low (0.5 nM) [13] and, as expected from its vital need, *H*. *pylori* possesses dedicated nickel uptake mechanisms. We previously identified the FrpB4 protein as the first TonB-dependent transporter mediating energized nickel uptake across the outer membrane [14]. Once nickel reaches the periplasmic space, it is transported through the inner membrane by the high affinity transporter NixA [15]. Then, nickel accumulates in the cytoplasm where its homeostasis and trafficking is controlled by several dedicated nickel-binding proteins that are only partially characterized [16,17]. As for other metal ions, the intracellular nickel concentration needs to be tightly controlled at the level of its uptake, storage and efflux. Indeed, non-physiological accumulation of free Ni(II) ions generates toxic effects such as interference with other metal-binding proteins or metal-dependent enzymes thereby disrupting essential catalytic functions that can lead to cell death [18].

Nickel trafficking displays several originalities in *H*. *pylori*. First, the maturation factors required for nickel incorporation into urease (UreE-F-G-H) and [NiFe] hydrogenase (HypA-HypB) are functionally overlapping as shown by genetic [19] and interactomic approaches [20]. This suggests the existence of a molecular cross-talk as well as a controlled nickel distribution strategy between these two enzymes [20]. Second, *H*. *pylori* possesses three atypical histidine-rich (His-rich) proteins, namely the heat shock protein A (HspA) and the two paralogous proteins Hpn and Hpn-2. Because poly-His peptides are known to bind nickel, it was proposed that the His-rich proteins found in *H*. *pylori* could serve as nickel stores [21,22]. We recently showed that expression of *hspA*, *hpn* and *hpn-2* genes is upregulated by the nickel-responsive transcriptional regulator NikR in response to nickel [23].

**Fig 1:**
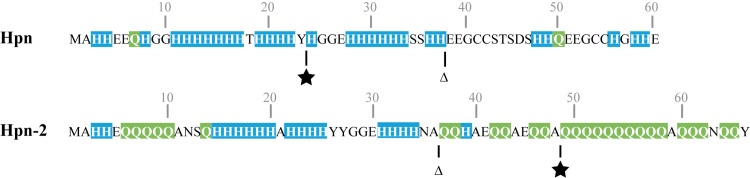
Sequences of the Hpn and Hpn-2 proteins of *H*. *pylori* strain B128 [27,28]. Histidine residues are highlighted in blue and Glutamine residues are in green. The triangle corresponds to the position at which Hpn is truncated in Hpn-∆C mutant and at which Hpn-2 is truncated in the Hpn-2∆C mutant. The black stars the positions at which the Hpn and Hpn-2 sequence are interrupted in the study of Seshadri et *al*. [29].

HspA, the sole homologue of the GroES chaperonin in *H*. *pylori*, displays a conserved C-terminal histidine and cysteine-rich (His- and Cys-rich) extension [24], absent from non-*Helicobacter* bacteria that behaves like a nickel sequestration domain [25]. This extension participates in nickel resistance and allows HspA to function as a dedicated chaperone for the maturation of [NiFe]-hydrogenase [25]. Hpn and Hpn-2 are atypical homologous proteins, because they contain an exceptionally high number of His residues, mostly clustered into stretches (Fig 1). Hpn, (HP1427 in strain 26695 [26]), was the first to be described [22]. It is a small (MW: 7.08 kDa) protein representing about 2% of total proteins in *H*. *pylori* [22]. It is composed of 60 amino acid residues among which 28 His and two Cys pairs (Fig 1). Hpn-2 (also designated Hpn-like or Hpnl), is a 66 amino-acid long protein (HP1432 in strain 26695) with a MW of 8.07 kDa [26]). Hpn-2 is similar to Hpn in its N-terminal half and central part (Fig 1). It is composed of 16 His residues (out of 34 amino acid residues), while its C-terminal region is strongly enriched in glutamine (Gln) residues (21 Gln out of 32 amino acid residues) (Fig 1).

Metal binding properties of Hpn and Hpn-2 have been characterized with purified recombinant proteins produced in *E*. *coli*. As expected from their high His content, both proteins can bind *in vitro* Ni(II) as well as other metal ions zinc (Zn(II)), cobalt (Co(II)), copper (Cu(II)) and bismuth (Bi(III)) and some residues important for binding were identified [30–34]. In solution, Hpn exists predominantly as a 20-mer [35] and each monomer binds 5 Ni(II) with a *K*_d_ of 7.1 μM. Purified Hpn can assemble *in vitro* into amyloid-like fibers, interact with membrane mimics and is cytotoxic to gastric epithelial cell cultures [36] [37]. The actual existence of such fibers in *H*. *pylori* needs however to be established. Hpn-2 appears to be a 22-mer in its native state, with one monomer binding 2 Ni(II) with a *K*_d_ of 3.8 μM [38]. The metal-binding capacity of Hpn and Hpn-2 has been addressed *in vivo* by FRET in *E*. *coli* using overexpressed engineered recombinant fluorescent proteins [39,40]. These studies revealed that Hpn did not bind nickel or zinc in *E*. *coli* while it was able to interact with Bi^3+^. In *E*. *coli* Hpn-2 is able to bind nickel and its C-terminal Gln-rich domain has little effect on metal binding [40]. Interestingly, when expressed in *E*. *coli*, Hpn and Hpn-2 both provided protection against nickel toxicity [38,41].

The *in vivo* function of Hpn and Hpn-2 has also been addressed in *H*. *pylori*. Initial studies reported that Hpn is an important player in protection against nickel and bismuth toxicity [42] and that complete deletion of the *hpn* gene does not alter urease activity [22]. More recently, inactivation of *hpn* and *hpn-2* genes by a cassette inserted approximately in the middle of the ORFs (Fig 1) suggested that Hpn-2 plays a major role in protection against nickel, while Hpn has only a minor role. The authors concluded that both proteins compete with the nickel-dependent urease maturation machinery under low nickel conditions [29]. Given these discrepancies, the respective roles of Hpn and Hpn-2 in *H*. *pylori* remained to be precisely established and were addressed by the present study.

The *Helicobacter* genus is composed of two subgroups, the enterohepatic species that infect the liver or gastrointestinal tract of mammals and some birds and a small group of gastric *Helicobacter* species (including *H*. *pylori*) [43]. Previous studies suggested that Hpn homologues are expressed in the gastric *H*. *mustelae* [22] and *H*. *felis* species [42] but the corresponding genes were neither identified nor annotated and Hpn homologues were never reported in other *Helicobacter* species. Phylogenetic distribution and evolutionary history of the atypical Hpn and Hpn-2 proteins consequently remained unknown and was established for the first time during our study.

In the present study, by combining assays to measure metal resistance, nickel accumulation and urease activity together with interactomic analysis, we provide evidence that Hpn and Hpn-2 act in a common pathway to control both nickel accumulation and urease activity in *H*. *pylori*. Using phylogenomic approaches, we determined the taxonomic distribution of *hpn* and *hpn-2* in the *Helicobacter* genus. Most interestingly, we showed that speciation of the gastric *Helicobacter* species from the other *Helicobacter* species occurred concomitantly with the emergence of *hpn*, while the duplication event at the origin of *hpn-2* took place in the common ancestors of *H*. *pylori* and *H*. *acinonychis* species. Using top-down proteomics, which is based on the analysis of intact proteins, without any digestion step, we were able to demonstrate the expression of these proteins in gastric *Helicobacter* species. In conclusion, our data suggest that acquisition of the nickel-binding protein Hpn was decisive for the gastric adaptation of *Helicobacter*. Accordingly, we observed that Hpn and Hpn-2 are both essential for colonization of the mouse stomach by *H*. *pylori*.

## Results

### Establishing a core-proteome-based phylogeny of the *Helicobacter* species

**Fig 2:**
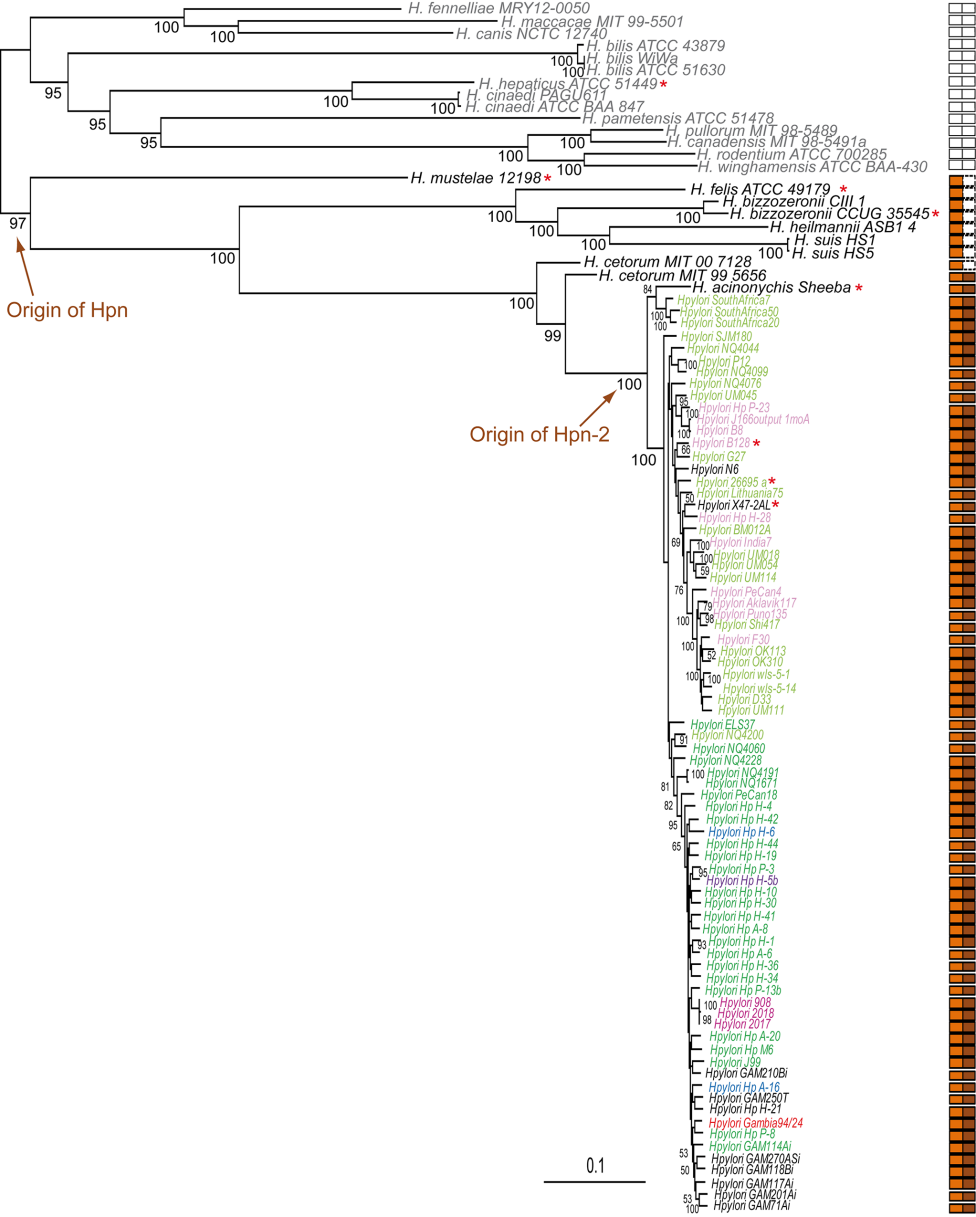
Maximum Likelihood phylogeny of a subsample of 100 out of the 330 *Helicobacter* strains analyzed in this study. The enterohepatic and gastric strains are shown in gray and black, respectively. The tree is based on the 281 protein families present in a single copy in at least 320 out of the 330 strains (82,743 amino acid positions). Numbers at nodes represent bootstrap supports (for clarity only values > 50% are shown). The scale bar indicates the average number of substitutions inferred by site. Orange and brown rectangles show the presence of *hpn* and *hpn*-2 in the corresponding strains, while orange and brown arrows indicate the origin of these genes. Strains harboring similar genomic contexts of *hpn* and *hpn-2* are indicated with similar color. Strains analyzed in this study are marked by an orange asterisk. Precise loci organizations are shown in Fig 3.

To begin our study of Hpn and Hpn-2, we decided to search for their presence and examine the taxonomic distribution of the corresponding genes in the available *Helicobacter* genomes. As a first step for this phylogenomic analysis, we establish a core-proteome-based phylogeny on 330 proteomes of *Helicobacter* from various geographical origins associated with different pathologies available at the NCBI (see Table 1 and S1 Table), including 305 *H*. *pylori* strains, 7 gastric and 11 enterohepatic non-*pylori Helicobacter* species (representing 10 and 15 strains of each respectively, see Table 1 and S1 Table). The core-proteome-based phylogeny of these strains was inferred using the 281 proteins present in single copy in at least 320 out of the 330 strains. The resulting phylogeny is relatively well resolved (most SH-like support (SH) values > 0.95) and showed a clear separation between the enterohepatic and the gastric strains (S1 Fig and Fig 2). While strains belonging to the same species clearly group together (*i*.*e*. for *H*. *suis*, *H*. *bizzozeronii*, *H*. *bilis* and *H*. *cinaedi*), our phylogeny revealed that the two *H*. *cetorum* strains MIT 00 7128 and MIT 99 5656 do not, the latter being more closely related to *H*. *acinonychis* and *H*. *pylori* (SH = 1.0, S1 Fig and Fig 2). This suggests that the two *H*. *cetorum* strains may rather correspond to two different species than to different strains of a same species. Regarding enterohepatic *Helicobacter*, we observed important discrepancies between our core-proteome-based phylogeny and 16S rRNA phylogenies (S1 Fig and Fig 2) that likely result from the use of a much larger number of positions in the core proteome phylogeny (1,500 nucleotide positions and 82,741 amino acid positions, respectively). We observed that *H*. *cinaedi* strongly grouped with *H*. *hepaticus* (SH = 1.0), while according to 16S rRNA trees it is more closely related to *H*. *bilis* [44]. Our tree supported also two robust clusters: *H*. *fennelliae*, *H*. *macacae*, and *H*. *canis* on the one hand and *H*. *pullorum*, *H*. *canadensis* and *H*. *rodentium* on the other hand (all SH = 1.0). In contrast, these species were described as dispersed in 16S rRNA trees [44]. Regarding non *H*. *pylori* gastric species, we observed a basal branching of *H*. *felis* (SH = 1.0), whereas this species represents the sister-lineage of *H*. *bizzozeronii* in 16S rRNA trees [44]. Finally, the large-feline strain *H*. *acinonychis* Sheeba [45] does not form a separate lineage, but instead emerges from within *H*. *pylori* strains, more precisely as a close relative of strains SouthAfrica7, SouthAfrica50 and SouthAfrica20 (SH-like support = 0.96). This is in agreement with previous observations suggesting that *H*. *acinonychis* results from a host jump from early humans to large felines about 200,000 years ago [45].

**Table 1:** List of the gastric and enterohepatic *Helicobacter* species analyzed in this study.

**Gastric *Helicobacter s*pecies**	**Strain**
*Helicobacter pylori*	434 different strains (see S1 Table)
*Helicobacter acinonychis*	Sheeba
*Helicobacter bizzozeronii*	CCUG 35545 and CIII 1
*Helicobacter cetorum*	MIT 00 7128 and MIT 99 5656
*Helicobacter felis*	ATCC 49179
*Helicobacter heilmannii*	ASB1 4
*Helicobacter mustelae*	12198
*Helicobacter suis*	HS1 and HS5
**Enterohepatic *Helicobacter s*pecies**	**Strain**
*Helicobacter bilis*	ATCC 43879, ATCC 51630 and WiWa
*Helicobacter canadensis*	MIT 98–5491 a and MIT 98–5491 b
*Helicobacter canis*	NCTC 12740
*Helicobacter cinaedi*	ATCC BAA 847 and PAGU611
*Helicobacter fennelliae*	MRY12-0050
*Helicobacter hepaticus*	ATCC 51449
*Helicobacter macacae*	MIT 99–5501
*Helicobacter pametensis*	ATCC 51478
*Helicobacter pullorum*	MIT 98–5489
*Helicobacter rodentium*	ATCC 700285
*Helicobacter winghamensis*	ATCC BAA-430

This novel core-proteome based phylogenetic tree provided us with a solid framework to study the distribution and evolutionary history of the *hpn* and *hpn-2* genes in the *Helicobacter* genus.

### *hpn* and *hpn-2* genes are specific to the gastric *Helicobacter* species

Surprisingly, Hpn and Hpn-2 proteins were only annotated in a few sequenced genomes of *H*. *pylori* (11 and 56 out of 305 in total, respectively) and in none of the other *Helicobacter* species (S1 Table). In several *H*. *pylori* genomes, longer ORFs in different frames overlapping the *hpn* and *hpn-2* predicted regions were annotated instead. We suspected that the *hpn* and *hpn-2* genes might have been missed because they encode small proteins with a highly biased amino acid composition. Therefore, we searched for ORFs predicted to encode these proteins in the corresponding genomic sequences. We found ORFs corresponding to Hpn and Hpn-2 in every *H*. *pylori* strain and in *Helicobacter acinonychis* (Fig 2 and S1 Table). Importantly, Hpn coding genes were detected in every gastric *Helicobacter* species, but in none of the enterohepatic species (Fig 2 and S1 Table).

### Scenario of the emergence and evolution of Hpn and Hpn-2 in the *Helicobacter* species

The strong similarity between Hpn and Hpn-2 (see multiple alignments in Suppl S2 Fig) indicates that these proteins are homologous and derived from a common ancestral sequence. Using the core-proteome based phylogenetic tree, we established the taxonomic distribution of *hpn* and *hpn-2*. It suggests that the *hpn* gene emerged in the last common ancestor shared by all gastric *Helicobacter* species whereas *hpn-2* originated later, likely through the duplication of *hpn*, in the lineage leading to *H*. *pylori* and *H*. *acinonychis* (Fig 2 and S1 Fig). To further investigate the evolution of *hpn* and *hpn-2* and determine whether horizontal gene transfer had affected their evolutionary history, we selected a subsample of 100 *Helicobacter* strains (*i*.*e*. 14 enterohepatic strains and 85 gastric strains, including 75 *H*. *pylori*) representative of the diversity of the 330 strains (Fig 2 and S1 Fig). We inferred the phylogeny of Hpn and Hpn-2 sequences present in the 75 *H*. *pylori* strains and in *H*. *acinonychis* strain Sheeba and compared it to the phylogeny of these 76 strains using the 281 core-genome markers mentioned above. The three topologies were compared with an approximately unbiased (AU) test [46]. The phylogeny of the 76 strains and that of Hpn-2 was significantly rejected by Hpn sequences, while the phylogeny of the 76 strains and that of Hpn was significantly rejected by Hpn-2 sequences (all p-values = 0.0). This indicates that the evolutionary histories of Hpn and Hpn-2 are different and do not follow the phylogeny of the strains, suggesting that horizontal gene transfer events had indeed occurred.

**Fig 3:**
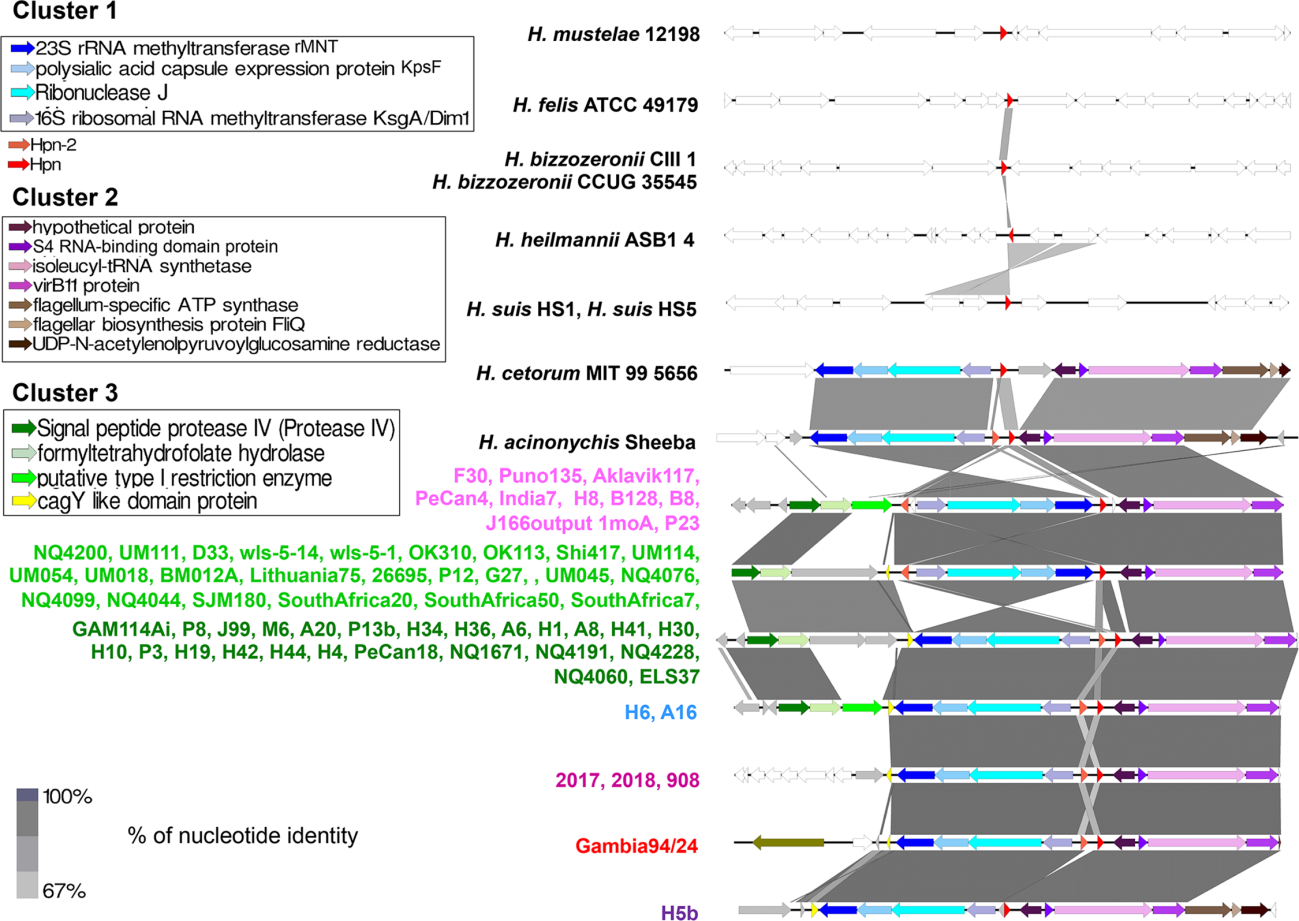
Genomic organization of the region encompassing *hpn* and *hpn-2*. Conserved homologous genes are shown with similar colors, hypothetical proteins are shown in gray, while non-conserved genes are in white. *Hpn* (in red) is located in-between two conserved cluster of genes (clusters 1 and 2), while *hpn-2* is located either near *hpn* on the same strand or on the opposite side of cluster 1 on the opposite strand. *Helicobacter* strains harboring similar genomic organization are listed on the left with similar colors.

The *hpn* genomic context in the 100 strains was examined. In *H*. *pylori*, *H*. *cetorum* and *H*. *acinonychis* genomes, *hpn* is located in a conserved region containing a gene cluster (cluster 1, in blue on Fig 3) composed of four genes corresponding to a 23S rRNA methyl-transferase (rMNT), a polysialic acid capsule expression protein (KpsF), the essential RNase J ribonuclease (RNAse J) [47] and the 16S rRNA dimethyladenosine transferase (KsgA). In contrast, in the other gastric species *H*. *mustelae*, *H*. *felis*, *H*. *bizzozeronii*, *H*. *heilmannii* and *H*. *suis*, we observed that *hpn* belongs to very different genomic regions (Fig 3). In *H*. *pylori*, the position of *hpn* and *hpn-2* relative to cluster 1 varies from one strain to another. In some strains, *hpn* and *hpn-2* surround cluster 1 and are expressed on different strands, whereas in other strains the two genes are adjacent and expressed on the same strand (Fig 3). These differences likely result from chromosomal inversions that might be the consequence of genomic recombination events. Therefore, we hypothesized that this inversion event has led to a duplication of *hpn* that gave rise to its paralogue *hpn-2*. In accordance with our conclusion of a more recent origin of Hpn-2, expansion of Gln residues occurred unequally in both the N and C-terminal regions of Hpn-2 whereas Hpn has remained nearly unchanged (S2 Fig). Finally, the genomic organization of the regions encompassing *hpn* and *hpn-2* is not fully consistent with the phylogeny of the strains. Certain closely related strains do not have the same genomic organization (e.g *H*. *pylori* NQ4060 and NQ4200, Figs 2 and 3), while distantly related strains may present a similar genomic organization (e.g. *H*. *pylori* F30 and B28). This suggests that comparable genomic reorganization events of this region occurred independently in different *H*. *pylori* strains, and/or that additional horizontal gene transfers took place at this locus (in agreement with the AU tests).

### Identification of Hpn and Hpn-2 proteins in *H*. *pylori* and in non-*pylori Helicobacter* species by top-down proteomics

Our survey of *Helicobacter* genomes led us to identify ORFs predicted to encode Hpn and Hpn-2 proteins in a misannotated DNA region. These proteins were not identified by the various proteomic studies published on *H*. *pylori* [48,49]. We speculated that these proteins have been missed because of their small size (<10 kDa) and because of the absence of Lys and Arg residues in their primary sequence, that precludes the use for their identification of classical bottom-up proteomics strategies based on trypsin digestion to generate peptidic fragments suitable for MS analysis. These two features make them however ideal candidates for top-down proteomics approaches [50,51]. Top-down proteomics is an emerging technology based on the analysis of intact proteins and thus does not involve enzymatic digestion steps. Therefore, we decided to use this technology to search for the Hpn and Hpn-2 proteins in the proteomes of *H*. *pylori* and of non-*pylori Helicobacter* species.

This study was performed with five selected representative strains belonging to the gastric *Helicobacter* group, namely *H*. *pylori* B128 (as a positive control), *H*. *acinonychis* (str. Sheeba), *H*. *felis* (ATCC 49179), *H*. *bizzozeronii* (CCUG35545) and *H*. *mustelae* (12198). In order to identify Hpn and/or Hpn-2 in total protein extracts from liquid cultures of these strains, we performed selective purification of the nickel-binding proteins, taking advantage of their property to strongly bind Ni-NTA agarose before their analysis by LC-MS/MS. Because the genomic regions containing the ORFs potentially encoding Hpn and Hpn-2 were misannotated, we manually implemented the sequences of these proteins in the corresponding databases before searching the proteomics data.

The top-down proteomics analysis procedure enabled us to unambiguously identify both Hpn and Hpn-2 proteins in *H*. *pylori* and in *H*. *acinonychis*. As a negative control, none of these proteins was detected in the enterohepatic species *H*. *hepaticus*. The Hpn protein (but not Hpn-2) was also identified in the gastric *Helicobacter* strains *H*. *felis*, *H*. *bizzozeronii* and *H*. *mustelae*. Interestingly, several proteoforms of Hpn were found in *H*. *mustelae* corresponding to N-terminal variants of the 3 first amino acid residues. The data are presented in Table 2. These results fully validate the *in silico* detection of *hpn* and *hpn-2* genes in the genomes of gastric *Helicobacter* group and confirms that both proteins are produced.

**Table 2:** Identification of Hpn and Hpn-2 proteins in samples prepared from different *Helicobacter* species by top-down proteomics.

Strain	Protein	Calculated mass, Da^1^	Measured mass, Da^2^	Mass diff., ppm	Max number of matching fragments^3^	E- value^4^
*Helicobacter pylori* B8	Hpn	6937.6567	6937.6584	0.3	50	2.26E-108
	Hpn-2	7922.4546	7922.4806	3.3	40	9.17E-71
*Helicobacter acinonychis* (Str. Sheeba)	Hpn	6969.7094	6969.7231	2.0	47	8.04E-104
	Hpn-2	7992.4496	7992.4586	1.1	12	6.75E-25
*Helicobacter bizzozeronii* (CGUG35545)	Hpn	6608.5422	6608.5614	2.9	38	2.03E-75
*Helicobacter felis* (ATCC 49179)	Hpn	6491.6014	6491.5812	-3.1	33	2.04E-65
*Helicobacter mustelae* (12198)	Hpn	7903.2685^5^	7903.3145	5.8	40	6.97E-72
		7772.2280	7772.2518	3.1	52	7.12E-91
		7511.1166^6^	7511.1444	3.7	50	1.39E-81
		7424.0846^7^	7424.1151	4.1	24	1.51E-42
		7208.9940^8^	7208.9918	-0.3	51	9.56E-100

1 –Calculated monoisotopic mass, cysteine residues oxidized to cystines.

2 –Experimental monoisotopic mass.

3 –At 5 ppm matching tolerance.

4 –Expectation value, calculated by ProSightPC software. The expectation value (e value) is the number of sequences in a database that are expected to have P-scores equal to or better than what was observed simply by chance. A P-score is the probability of obtaining at least as good a match between the observed fragment list and a sequence as by chance. Low E-values represent better matches (less likely to be false positives) than high E-values.

5, 6, 7, 8 –forms of the protein with N-terminal Met retained (5) or N-terminal NF (6), NFS (7) or NFSTN (8) residues cleaved.

### Hpn but not Hpn-2 plays a major role in protection against nickel overload

**Fig 4:**
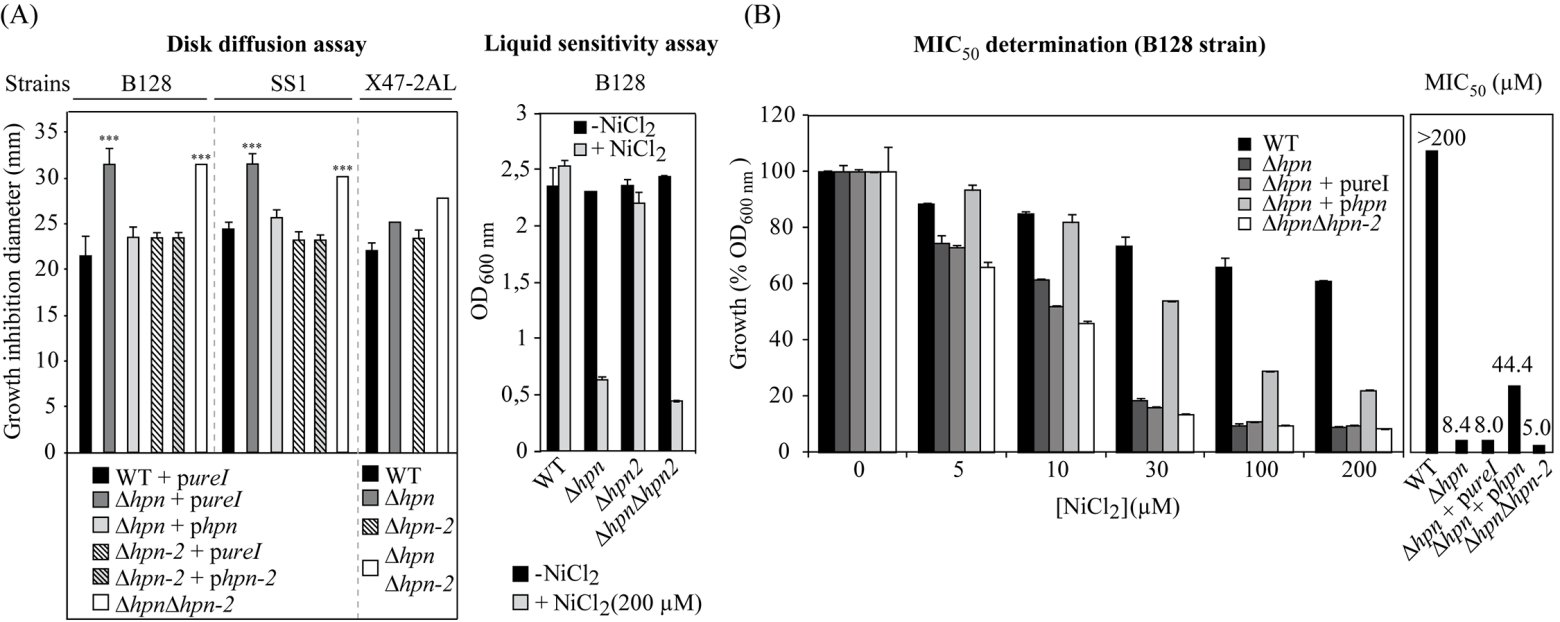
Effect of nickel on the growth of *H*. *pylori* B128, SS1 and X47-2AL wild type strain and isogenic mutants. The strains with pureI are controls in which only the pUreI is inserted at the locus at which the *hpn* or *hpn-2* genes are introduced in the complemented strains *∆hpn + phpn* and *∆hpn-2* + *phpn-2*. Panel A, nickel disk diffusion assay and sensitivity to 200 μM NiCl_2_ in liquid medium. Panel B, nickel sensitivity in liquid medium (range of NiCl_2_ concentrations) of mutants and MIC_50_ for the B128 ∆*hpn*, ∆*hpn* +p*hpn* and ∆*hpn-∆hpn-2* strains. The data correspond to the mean value of three independent experiments with at least triplicate tests for each strain. Error bars represent the standard deviation.

The His-rich composition of Hpn and Hpn-2 and previous data prompted us to examine the importance of these proteins in the sensitivity of *H*. *pylori* to nickel overload [29,42]. This was evaluated in different genetic backgrounds (B128, SS1, X47-2AL and 26695) using mutants carrying complete deletions of *hpn* or *hpn-2* and of both genes (Fig 4 and S3 Fig for strain 26695). The *hpn* and *hpn-2* single mutants in the B128 and the SS1 backgrounds were complemented *in cis* by the wild type version of these genes. Two methods were used to examine the sensitivity of these strains to nickel. Zones of inhibition (ZOI) were measured by the disc diffusion assay for the different strains (Fig 4). A more precise quantitation was performed in liquid cultures with strain B128 with increasing nickel concentrations allowing measurement of the minimal inhibitory concentration of nickel at which growth was reduced by 50% (MIC_50_) (Fig 4). The ZOI tests gave almost identical results in the different genetic backgrounds (Fig 4). The *∆hpn-2* mutant and the complemented *∆hpn-2* strain presented the same tolerance as the parental strain to increasing nickel concentrations (Fig 4). In contrast, the *∆hpn* mutant and *∆hpn ∆hpn-2* double mutant showed an identical significant increase in nickel sensitivity (MIC_50_ 8.4 and 5.0 μM, respectively) as compared to the parental strain (MIC_50_ >200 μM) (Fig 4). Nickel sensitivity of the ∆*hpn* mutant was complemented by an ectopic chromosomal copy of the wild type *hpn* gene and this complementation was only partial (MIC_50_ 44 μM in liquid culture) presumably because of insufficient expression of the ectopic *hpn* copy.

These data indicate that the Hpn protein is crucial to protect *H*. *pylori* against nickel overload while Hpn-2 is not required for this function.

### Hpn but not Hpn-2 contributes to *H*. *pylori* intracellular nickel storage

**Fig 5:**
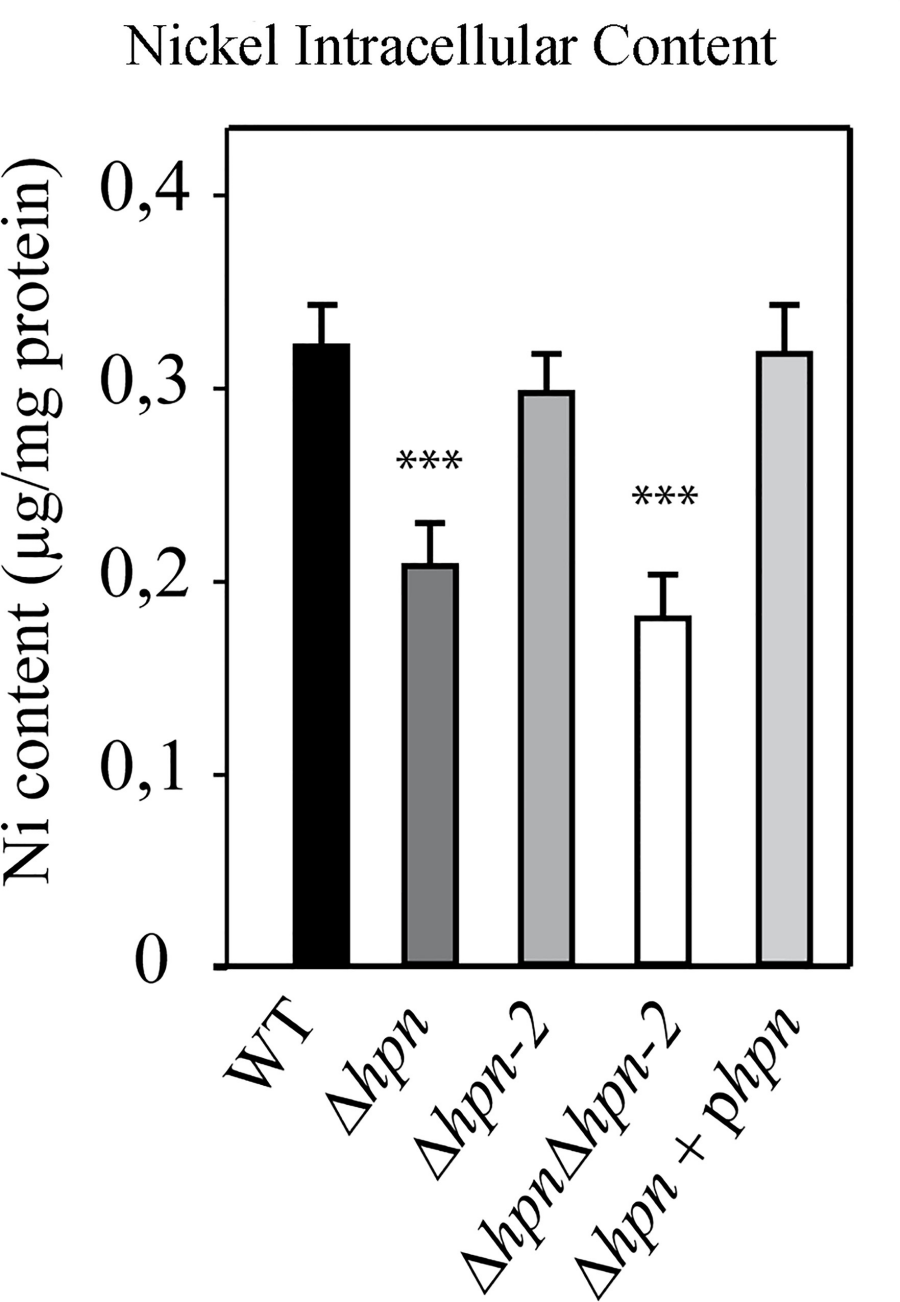
Intracellular nickel content of *H*. *pylori* B128 wild type strain, isogenic mutants and complemented strains. The identity of each strain is indicated below the bars. Nickel amounts were measured by Inductively Coupled Plasma Optical Emission Spectrometry (ICP-OES) and expressed in μg of nickel. mg of proteins^-1^. The data correspond to the mean value of two independent experiments with at least triplicate tests for each strain. Error bars represent the standard deviation. *** indicates that the mean value is significantly different from that of the wild type strain (*P* < 0.01).

To evaluate the role of Hpn and Hpn-2 in the control of nickel homeostasis in *H*. *pylori*, the total intracellular nickel content was measured by Induced-Coupled-Plasma Optical Emission Spectrometry (ICP-OES). Nickel was measured in the parental B128 strain, isogenic mutants and complemented strains exposed to NiCl_2_ (200 μM) as in our previous studies [14]. When bacteria were incubated without nickel, the intracellular nickel was below the detection limit of ICP-OES. Upon exposure to nickel, the wild-type strain contained 0.3 μg of nickel per mg of proteins (Fig 5). In all experiments, the intracellular nickel content of the *∆hpn-2* mutant was identical to that measured in the wild type strain. In contrast, we observed a decrease in the intracellular nickel content in the mutant strains ∆*hpn* (1.6 fold), and ∆*hpn∆hpn-2* (1.8 fold) as compared to the wild-type strain. Trans-complementation of ∆*hpn* with a wild type *hpn* copy restored wild type nickel content under these conditions. This result indicates that Hpn but not Hpn-2 participates in the accumulation of nickel *in vivo*.

### Both Hpn and Hpn-2 control urease activity

**Fig 6:**
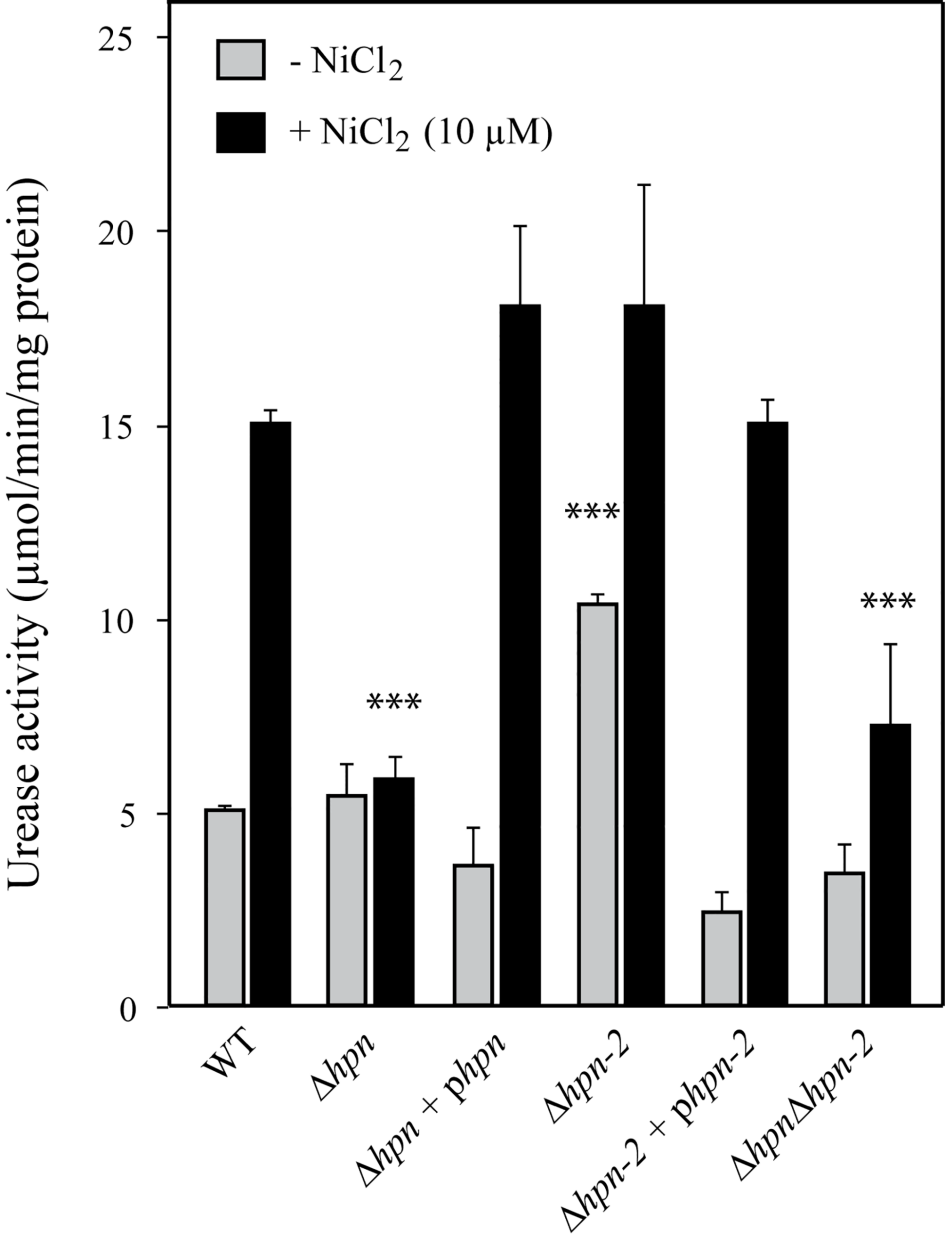
Urease activity of *H*. *pylori* B128 wild type strain, isogenic mutants and complemented strains. Strains were grown in Brucella β-cyclodextrin medium without added nickel (grey bars) or with 10 µM nickel (black bars). The identity of each strain is indicated below the bars. Urease activity is expressed as μmol of urea hydrolyzed. min^-1^. mg^-1^ of protein. The data correspond to the mean value of three independent experiments with at least triplicate tests for each strain. Error bars represent the standard deviation. *** indicates that the mean value is significantly different from that of the wild type strain (*P* < 0.01).

We then evaluated the influence of the Hpn and Hpn-2 proteins on the activity of hydrogenase and urease, the two nickel-containing enzymes of *H*. *pylori*. Hydrogenase activity, measured as previously described [25], was not altered in the mutant strains grown in BBβ medium supplemented or not with nickel. Urease activity was monitored by measuring NH_3_ production of intact bacteria incubated in BBβ with 5 mM urea (Fig 6). Nickel concentration in BBβ medium is about 0.2 μM [52]. We compared urease activities of wild type and mutant strains grown without or with 10 μM NiCl_2_. As expected, urease activity of the wild type strain was enhanced (about 3-fold) in the presence of 10 μM NiCl_2_. In non-supplemented medium, urease activity of the *∆hpn* mutant was identical to the parental strain. Interestingly, nickel-dependent urease activation was lost in this latter mutant, and restored to wild type level upon complementation (∆*hpn* + p*hpn*) (Fig 6). In addition, we found that under nickel-restricted conditions, urease activity of the ∆*hpn-2* mutant was significantly enhanced (2-fold) as compared to the wild type strain, a phenotype that was restored by complementation with a wild type *hpn-2* gene. Upon nickel addition, urease activity of the *∆hpn-2* mutant increased to wild-type levels. We concluded that Hpn is necessary for full urease activation by nickel, while Hpn-2 directly or indirectly restricts basal urease activity under low nickel conditions. Surprisingly, in contrast to the single *∆hpn-2* mutant, basal urease activity of the *∆hpn∆hpn-2* double mutant was similar to that of the wild type strain. This indicates that *hpn* deletion suppresses the phenotype of the *∆hpn-2* mutant. Upon nickel addition, urease activity of the double mutant is slightly induced and reached a lower level than the wild type strain.

Western blotting using anti-UreA antibody was performed to test whether the differences in urease activity of the mutants were caused by changes in the amounts of urease (S4 Fig). Under nickel-restricted conditions, UreA levels were similar in all strains tested. As expected, the expression of UreA was induced in the presence of nickel (2-fold after 8 hours). We concluded that the differences in urease activities displayed by the mutants are not due to variation in urease amounts but rather to modification in nickel loading into the enzyme.

Taken together, these results suggest that Hpn and Hpn-2 genetically interact in a common pathway to control urease activation, possibly at the level of nickel insertion.

### Protein interaction networks between the nickel trafficking proteins

**Fig 7:**
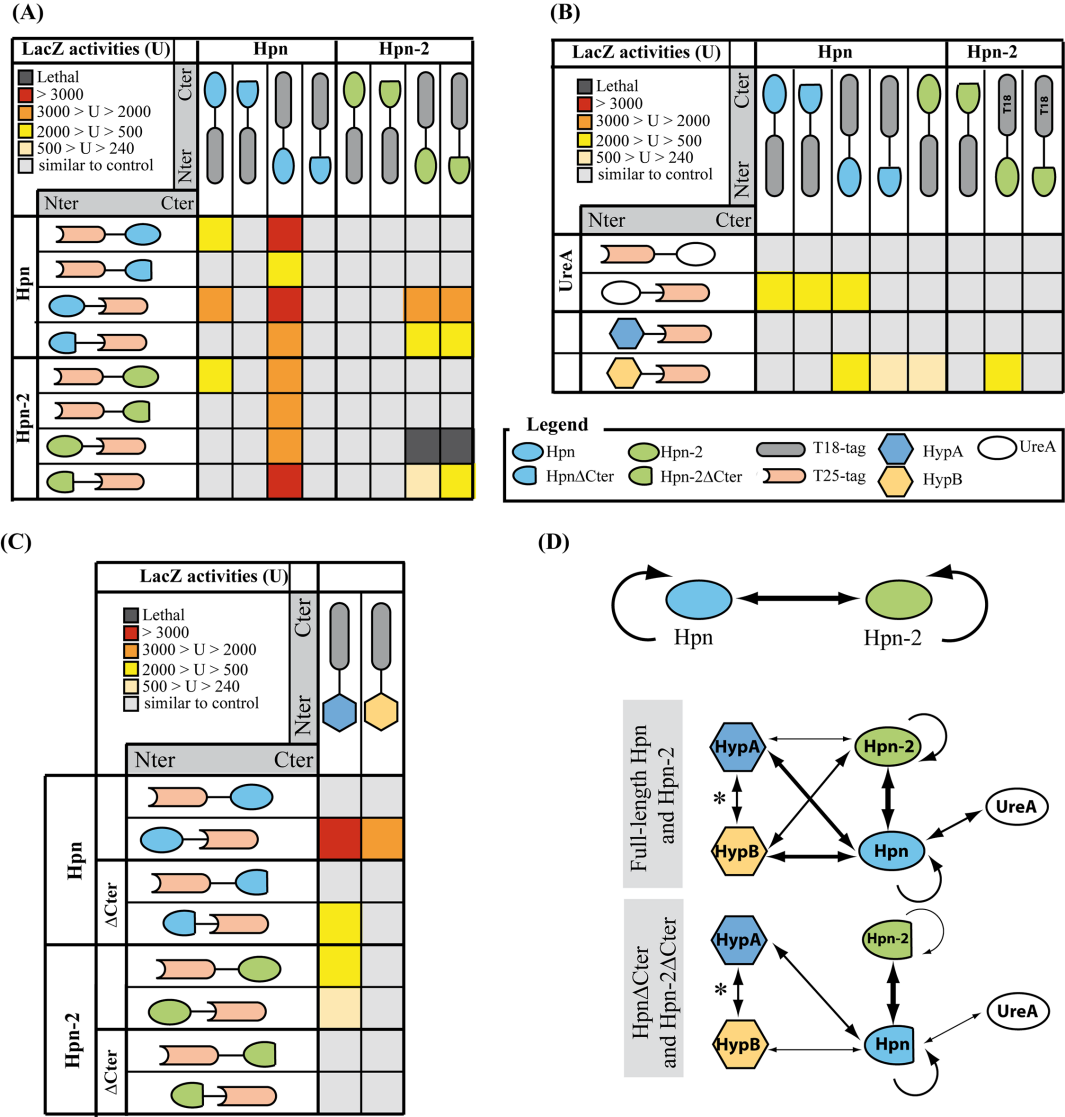
Analysis of the protein-protein interaction network of Hpn and Hpn-2 by *in vivo* BACTH. (A-C) the tables represent the results of the β-galactosidase activity measurements on *E*. *coli* strains containing the pair-wise combinations of the different constructs. T18 and T25 correspond to the two fragments of the adenylate cyclase and the protein extremity at which the fusion was made (N_ter_ or C_ter_) is shown. Panel A: interaction between Hpn, Hpn∆Cter, Hpn-2 and Hpn-2∆Cter. Panel B and C: interaction of Hpn, Hpn∆Cter, Hpn-2 and Hpn-2∆Cter with UreA, HypA and HypB. Red squares correspond to β-galactosidase activity that are > 3,000 U, orange squares between 2,000 and 3,000 U, yellow squares between 500 and 2,000 U and light yellow squares, between 240 and 500 U. None of the potential false positive interactions was presented (interactions of pUT18/pKNT25(Hpn) and pUT18(Hpn)/pKNT25 with ß-galactosidase activity below 600 units). Black squares represent combinations that lead to a lethal phenotype. The β-galactosidase activity values for each strain and the controls are indicated in S2 Table. (D) Schematic representation of the protein-protein interaction network of Hpn and Hpn-2. Thick arrows correspond to strong interactions, thin arrows to moderately strong interactions, according to the ß-galactosidase. * Indicates that this interaction was established before [53].

Previous studies showed that *H*. *pylori* Hpn and Hpn-2, purified from *E*. *coli*, each form homo-multimers *in vitro* [35,38]. Our data suggest that in *H*. *pylori* Hpn and Hpn-2 act in a common pathway to regulate urease activity. We consequently explored the *in vivo* interactions of these two proteins with each other, and with (i) the urease structural subunits (UreA-B), (ii) the urease accessory proteins involved in nickel incorporation (UreE-F-G-H), (iii) the urea channel (UreI) and finally (iv) the HypA-B urease/hydrogenase maturation subunits that were known to interact with each other [53]. In addition, we tested the influence of the C-terminal regions of Hpn and of Hpn-2 on the interactions (deletion sites represented in Fig 1). For this large study, we used the bacterial two-hybrid approach that tested pairwise interactions in *E*. *coli* [54]. The *hpn*, *hpn-2*, *hpn∆Cter* and *hpn-2∆Cter* genes were cloned in frame at their 5’ or 3’ extremities, with fragments encoding either the N-terminal (T25) or the C-terminal part (T18) of the *Bordetella pertussis* adenylate cyclase resulting into four different constructs for each protein. The *ureA*, *ureB*, *ureI*, *ureE*, *ureF*, *ureG*, *ureH*, *hypA* and *hypB* genes were cloned in frame at their 3’ extremities with the T25 and T18 fragments. The different plasmids were transformed into *E*. *coli* in combination of pairs and the protein-protein interactions were evaluated by measuring the expression of the *lacZ* reporter gene. The results are illustrated in Fig 7 and the ß-galactosidase activity data are in S2 Table. As a control, background adenylate cyclase activity was measured for each fusion plasmid by co-transforming *E*. *coli* with the compatible empty vector. Only the two combinations pUT18/pKNT25(Hpn) and pUT18(Hpn)/pKNT25 presented relatively high background ß-galactosidase activity 400 and 600 units respectively (light green in S2 Table). Therefore, interactions with these fusions were only considered as positive when ß-galactosidase activity was significantly higher than 600 units, thereby avoiding false positives.

First, all four combinations of plasmids expressing Hpn fusions scored positive in ß-galactosidase assays, showing that Hpn is able to form homo-oligomers *in vivo*. The C-terminal region of Hpn region is important but not essential for this interaction.

Concerning the interaction of Hpn-2 with itself, we observed that co-transformation of the plasmids expressing pKNT25(Hpn-2) fusion protein with either pUT18(Hpn-2) or pUT18(Hpn-2∆C) was lethal (materials and methods). We suspect that this is due to very strong Hpn-2/Hpn-2 interaction, resulting in extremely high adenylate cyclase activities that are toxic to *E*. *coli*, possibly through depletion of the ATP pool (as already observed by D. Ladant, personal communication). The pKNT25(Hpn-2∆C) fusion interacted with both pUT18(Hpn-2) or pUT18(Hpn-2∆C). In this context, our results strongly suggest that Hpn-2 is able to form homomultimers and that its C-terminal Gln-rich region is important but not essential for this interaction (Fig 7).

We then examined the interactions between Hpn and Hpn-2. Four out of the eight combinations of Hpn and Hpn-2 fusions scored highly positive, indicating very strong interaction between Hpn and Hpn-2.

The interactions of Hpn and Hpn-2 with the urease structural subunits and accessory proteins were then tested. No interaction was detected with UreB, UreE, UreH, UreF and UreG, neither with the UreI urea channel protein. Importantly, UreA presented strong interaction with Hpn but not with Hpn-2, an interaction that was apparently reduced with the Hpn∆C fusion (Fig 7). Finally, both Hpn and Hpn-2 interact with HypA and HypB, the two hydrogenase accessory proteins. The ß-galactosidase activities of the Hpn/HypA and Hpn/HypB combinations were higher than those with Hpn-2. Deletion of the Hpn-2 C-terminal region resulted in a complete loss of its interaction with HypB.

In conclusion, these data show that Hpn and Hpn-2 form both homo- and hetero- multimeric protein complexes *in vivo* (Fig 7). In addition, Hpn strongly interacts with the UreA structural subunit and both Hpn and Hpn-2 interact with HypA and HypB. The Hpn/Hpn-2 protein-protein interaction network summarized in Fig 7 is perfectly in line with the data presented above suggesting that Hpn and Hpn-2 participate in a common pathway of nickel transfer.

### Hpn and Hpn-2 are essential for colonization of the mouse model

**Fig 8:**
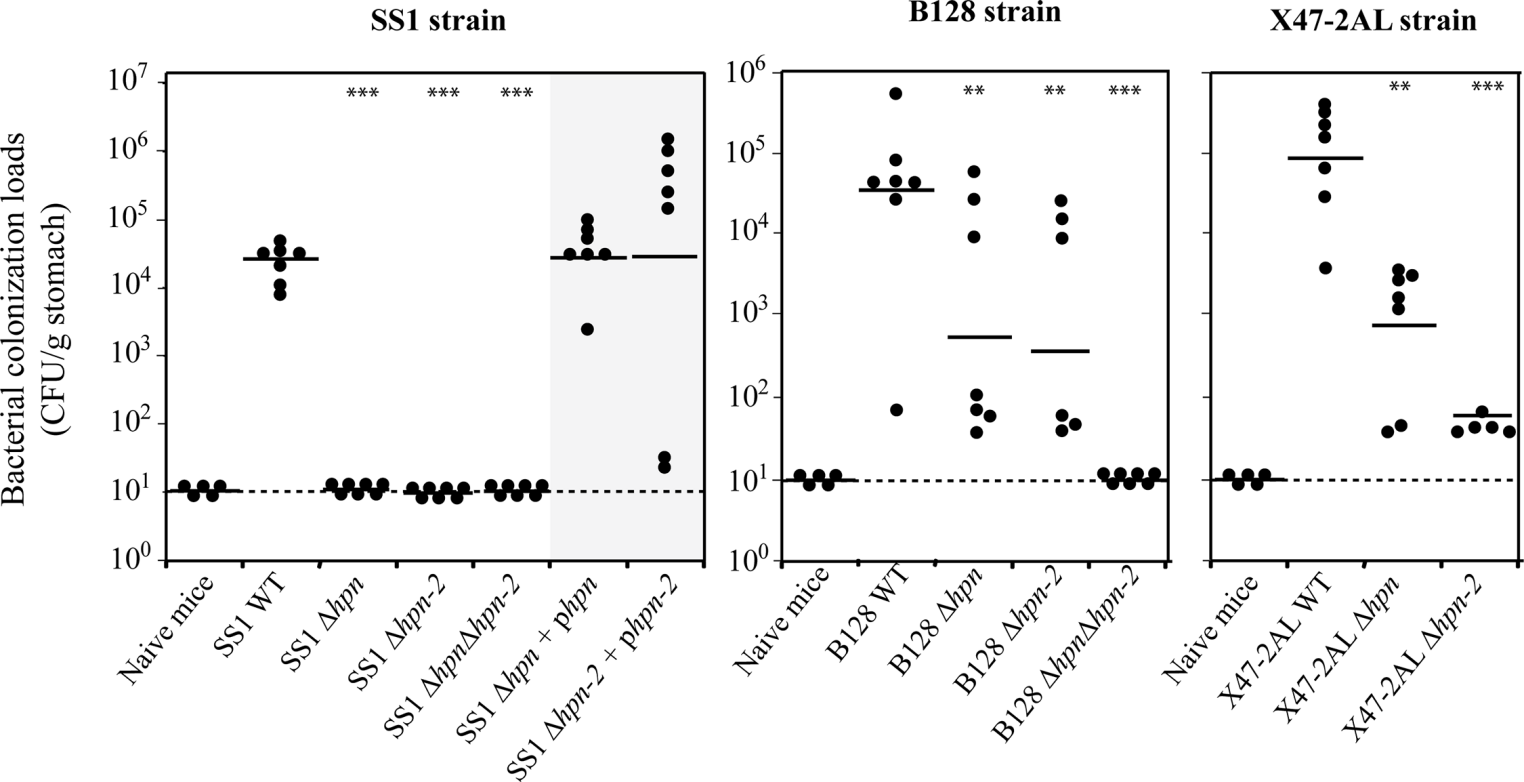
Hpn and Hpn-2 are required for full mouse colonization by *H*. *pylori* SS1, B128 and X47-2AL strains. Each point corresponds to the colonization load for one mouse, with the strain indicated below. Each strain was tested in a group of seven mice. Horizontal bars represent the geometric means of the colonization load for the wild type and each mutant. The detection limit is shown by a dashed horizontal line. *** and ** indicate that the geometric mean is significantly different from that of the wild type strain (*P* < 0.01 and *P* < 0.05, respectively).

The role of Hpn and Hpn-2 during gastric colonization was evaluated using the mouse model. Three different *H*. *pylori* strains able to colonize the mouse stomach were tested: SS1, X47-2AL and B128. Mutants carrying complete deletions of *hpn*, *hpn-2* and of both genes were constructed. In the SS1 background, the *∆hpn* and the *∆hpn-2* mutants were complemented *in cis* by the corresponding wild type genes. All strains were orogastrically inoculated to seven NMRI mice per strain. One month later, colonization was assessed by quantitative cultures of stomach homogenates (Fig 8). We observed that depending on the genetic background, the single *∆hpn* and *∆hpn-2* mutants were either strongly affected or completely deficient in their ability to colonize the mouse stomach. The complemented SS1 mutants recovered the capacity to colonize the murine stomach. Importantly, the double *∆hpn∆hpn-2* mutant was totally unable to colonize the mouse stomach in every genetic background.

These results revealed for the first time a crucial role of Hpn and Hpn-2 during *in vivo* colonization.

## Discussion

The relation between metal trafficking and bacterial virulence has attracted large interest these last years. In addition to uptake and efflux, it is now admitted that cytoplasmic concentration of transition metals is often controlled by specific proteins that can detoxify and/or act as metal reservoirs that can be mobilized when necessary [55]. In *H*. *pylori*, nickel is required for stomach colonization as it is the cofactor of two enzymes essential in the animal model, the very abundant urease and the [NiFe] hydrogenase [11]. Large amounts of nickel must be acquired by *H*. *pylori* in order to supply these enzymes with their metal cofactor. This bacterium expresses three His-rich proteins that were proposed to serve as nickel stores, HspA, Hpn and Hpn-2. We previously showed that HspA is a nickel sequestration protein and a specialized Ni(II) chaperone for hydrogenase [25]. However, little information was available regarding the *in vivo* role of the two other His-rich proteins of *H*. *pylori*, Hpn and Hpn-2. In addition, nothing was known on the evolution and phylogenetic distribution of these unusual proteins in bacteria.

### Role of Hpn in the control of intracellular nickel content of *H*. *pylori*

In a previous study, Hpn-2 and Hpn were reported to play respectively a major and a minor role in nickel tolerance [29]. In the present work, we found in agreement with Mobley *et al* [42], that a mutant carrying a complete *hpn* deletion is much more sensitive to the toxic effects of nickel than the corresponding wild type strain. Lower intracellular nickel amounts accumulated in the *hpn* deletion mutant, a phenotype that was restored to wild type concentrations in the complemented strain. This suggests that Hpn is sequestering nickel *in vivo* in agreement with its capacity to bind nickel *in vitro* [35]. In contrast, we observed that Hpn-2 is not required for protection against the deleterious effects of nickel and has no influence on the intracellular nickel content. Strengthening our conclusions, we observed identical phenotypes for the mutants in four different genetic backgrounds (B128, X47-2AL, SS1 and 26695) and recomplemented the Hpn phenotype in two of these strains (Fig 4). One plausible explanation for the discrepancy between our data and Seshadri’s work [29] is that their *hpn* and *hpn-2* mutants were constructed by insertion mutagenesis implying that N-terminal fragments of both proteins are most likely still expressed and may partially retain a function because of their repetitive composition (for Hpn a fragment with 14 His out of 27 and for Hpn-2 a fragment with 26 His out of 27, shown in Fig 1). In contrast, our analysis was performed with complete deletion mutants and allowed us to unambiguously attribute a major role of Hpn in nickel sequestration.

Small iron-binding proteins that are able to form oligomeric structures, such as ferritins [56], have long been known to be involved in storage and detoxification of this metal. Given the *in vitro* behavior of Hpn, the phenotypes associated with its depletion and the fact that it represents about 2% of the total protein content of *H*. *pylori*, it is tempting to attribute a similar function to Hpn. *In vitro*, Hpn can bind copper, zinc and bismuth in addition to nickel [32,35,40]. However, our phenotypic analysis of the *∆hpn* mutant indicates that besides nickel storage/detoxification, Hpn might additionally be involved in protection against bismuth (our data and previous work by [42]).

Finally, further experiments are needed to understand how metal ions bound to Hpn are mobilized and delivered to other proteins *in vivo*, whether, as we suspect, Hpn-2 is involved in this process and finally if metal binding to Hpn and Hpn-2 controls their multimerization and interaction.

### Control of urease activity by Hpn and Hpn-2

**Fig 9:**
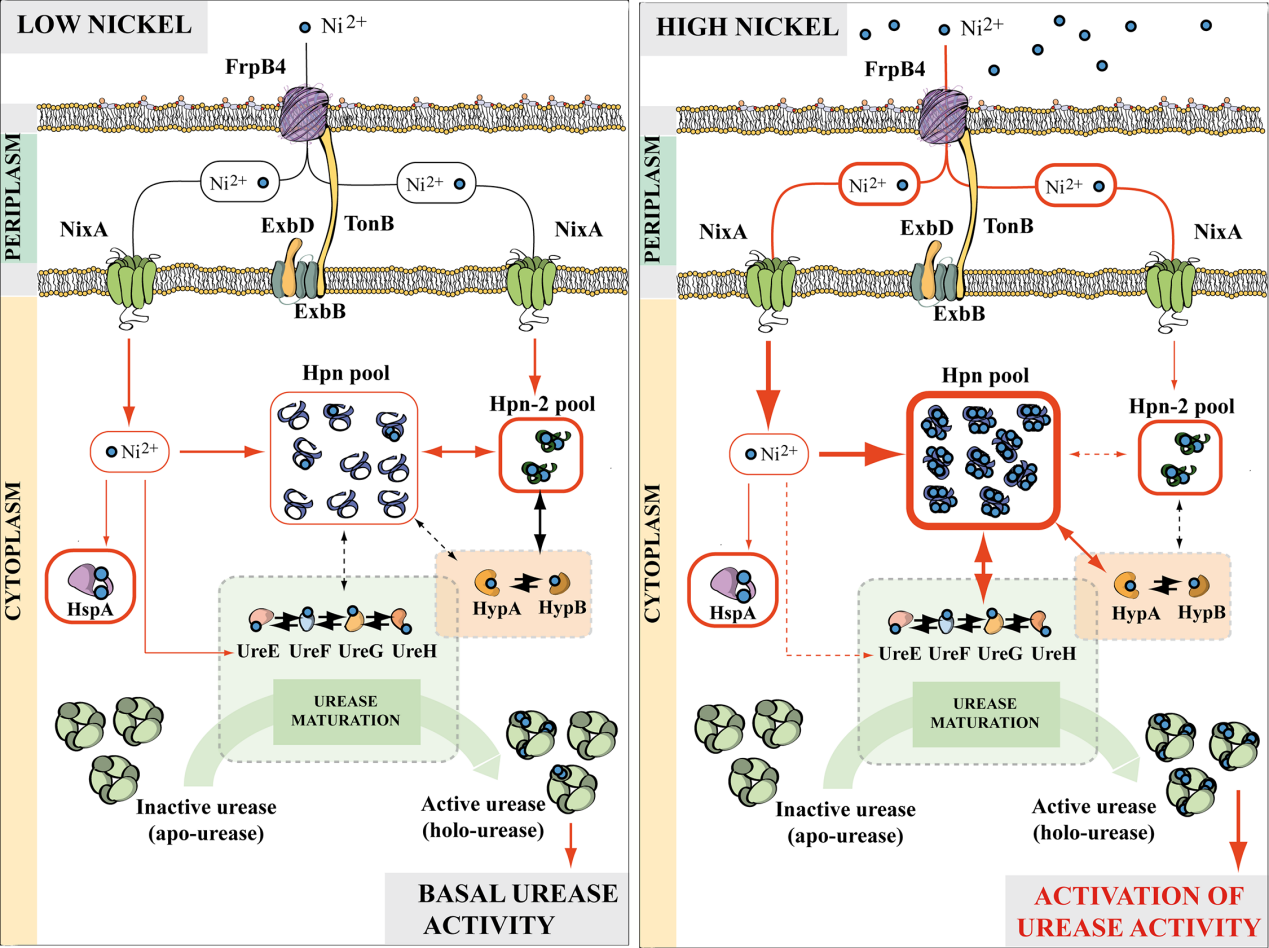
Model of the role of Hpn and Hpn-2 in nickel trafficking *H*. *pylori*. In *H*. *pylori*, nickel is transported across the outer membrane by FrpB4, a TonB-dependent transporter. Once in the periplasm, uptake through the inner membrane occurs through the NixA permease (green). In the cytoplasm, nickel can follow several pathways, via the UreE/F/G/H or HypA/B accessory proteins dedicated to urease and/or hydrogenase maturation, the HspA co-chaperonine and Hpn or Hpn-2. **(A)** Under low nickel concentrations, and because Hpn is abundant, the Hpn cellular pool is not saturated with nickel, while Hpn-2, which is less abundant, can compete with Hpn (similar affinities for nickel). Nickel transfer from Hpn to the urease maturation machinery is low. This results in the “basal” urease activity level monitored in wild type *H*. *pylori* cells. **(B)** Under high nickel conditions, the Hpn protein pool stores more nickel and becomes saturated with this metal. Since Hpn-2 is less abundant, it rapidly becomes outcompeted. Under such conditions, interactions between Hpn and urease and/or hydrogenase maturation proteins enables Hpn-stored nickel transfer towards urease, leading to an increase in nickel incorporation, and enhanced urease activity.

Phenotypic and enzymatic assays on *H*. *pylori* strains revealed that Hpn is a key player in nickel homeostasis, as well as in the control of urease activity. The basal urease activity of the *∆hpn* mutant is similar to that of the wild-type strain, in contrast no increase in urease activity of this mutant was observed under conditions of high exogenous nickel. This suggests that Hpn is involved in activation of the cellular urease pool, when nickel increases. Given that Hpn is a nickel-binding protein involved in protection against metal overload, it is tempting to assign a direct role of Hpn in nickel delivery to urease or urease maturation machinery. Our two-hybrid experiments suggest that Hpn may achieve this role through direct interaction with the structural protein UreA, but not with the UreE-F-G-H accessory proteins. In bacteria, nickel incorporation into urease requires UreE that acts as a nickel chaperone [9]. Compared to other bacteria, the *H*. *pylori* UreE has a lower nickel-binding capacity (1 Ni(II) ion per monomer) and a poor content in His residues [57] [58]. These molecular features might limit the overall nickel incorporation capacity of the urease maturation machinery, when intracellular nickel concentration increases. Based on our enzymatic and two-hybrid experiments, we propose the following model illustrated in Fig 9. Hpn would directly interact with urease (UreA) and increase the nickel incorporation capacity of the maturation machinery, thereby compensating for this limitation. We observed that Hpn also interacts with HypA and HypB (Fig 7), proteins that have been both reported to participate in nickel incorporation into both urease and hydrogenase [19], which could represent an alternative and/or additional pathway influencing the nickel incorporation capacity.

The role of Hpn-2 is more intriguing. Our data revealed that Hpn-2 restricts basal urease activity, since the *∆hpn-2* mutant presented enhanced urease activity under low nickel conditions, whereas at higher nickel concentrations the mutant behaved like a wild type strain (Fig 6). This phenotype depends on the presence of Hpn. Indeed, the ∆*hpn* mutation was found to be dominant over the *∆hpn-2* mutation in the *∆hpn∆hpn-2* mutant suggesting that the two proteins are involved in a common pathway of nickel transfer to urease (Fig 6). In the *∆hpn-2* mutant, urease activity restriction under low nickel conditions could be due to a yet unknown, direct or indirect, regulatory or physiological mechanisms. However, since our two-hybrid experiments provided evidence for a direct interaction of Hpn-2 with Hpn (but not with UreA), and also with HypA and HypB, we propose the following model (Fig 9). This model takes into account these interactions and the fact that Hpn and Hpn-2 were shown to have similar affinities for nickel, but to differ in their metal binding capacity (5 and 2 nickel ions per monomer respectively) and the fact that Hpn is much more abundant than Hpn-2 in *H*. *pylori*. When nickel availability is low, the abundant Hpn proteins would not be saturated with nickel. Since Hpn and Hpn-2 strongly interact, we propose that competition for nickel through direct interaction would occur, resulting in limited transfer of nickel from Hpn to urease, thereby restricting its activation. At higher nickel concentrations, the nickel-bound Hpn pool would strongly increase, while Hpn-2 might readily become saturated with nickel because of its lower abundance. Under these conditions, competition with Hpn for nickel would become negligible and would fail to impact urease activation. However, given the importance and complexity of nickel trafficking in *H*. *pylori*, we cannot exclude the participation of additional proteins responding to nickel, this will be the subject of future investigations.

### *In vivo* essentiality of Hpn and Hpn-2

Our results reveal for the first time that Hpn and Hpn-2 proteins are individually required for full colonization of mice by *H*. *pylori*. This was demonstrated with three different *H*. *pylori* mouse-adapted strains (B128, X47-2AL and SS1). Complementation of the mutations by wild type *hpn* or *hpn-2* genes restored full colonization. In addition, the *∆hpn-∆hpn-*2 double mutants were totally unable to establish infection. Our results revealed that Hpn and Hpn-2 are involved in nickel storage and regulation of urease activity. The essentiality of these proteins during gastric colonization is possibly associated to the higher solubility of nickel under acid conditions and the strict dependence of *H*. *pylori* on high and tightly controlled urease activity for acid resistance. We cannot exclude that these proteins have additional essential functions *in vivo*. Further work is required to explore this possibility.

Our results contrast with those of Benoit *et al* [59] who reported wild type colonization of the mouse model by a *∆hpn-∆hpn-*2 double mutant and observed an anecdotic 3-fold lowering of colonization loads in mice that were fed with a Ni-deficient chow. Unfortunately, this paper does not mention the mouse lineage used for colonization and the *H*. *pylori* mutants were not further characterized. Given the lack of information in the other study, the reason for the discrepancy between our results consequently remains enigmatic, one possibility might be the difference in mouse lineage and/or in diet.

Our data are consolidated by the fact that we obtained consistent results with well-characterized mutants in three different genetic backgrounds as well as restoration of the observed phenotypes in complemented mutants.

### Evolutionary history of Hpn and Hpn-2, two proteins required for *in vivo* colonization

The experimental data presented here support the existence of a nickel trafficking pathway in *H*. *pylori*. Our phylogenomic analysis showed that *hpn* and *hpn-2* genes, encoding two key players of this pathway, are present in every available *H*. *pylori* complete genome as well as in *H*. *acinonychis*. These two genes are thus part of the *H*. *pylori* and *H*. *acinonychis* core-genome underlining their important role for these organisms. *hpn-2* gene is restricted to these two species. In contrast, *hpn* genes were predicted in every available genome of gastric *Helicobacter* species, whereas it was absent from the genomes of enterohepatic *Helicobacter* species (Fig 2 and S1 Fig). The sequence of Hpn in *H*. *mustelae*, the first diverging gastric lineage (Fig 2 and S1 Fig), deserves attention since it contains 11 [GEH_3_] motifs corresponding to [GGNGAG(CAT)_2_CA] repeats (S2 Fig). This indicates that short tandem duplications of an ancestral [GGNGAG(CAT)_2_CA] motif could be at the origin of *hpn*. Every Hpn sequence from the gastric strains contains one or two -CC- motifs located either in their N-terminus (*H*. *suis*, *H*. *heilmannii*, *H*. *bizzozeronii*, *H*. *felis*) or C-terminus (*H*. *pylori*, *H*. *acinonychis* and *H*. *cetorum*) (S2 Fig). We propose that these motifs are functionally important, as cysteine pairs are often involved in metal binding [30]. Our survey of the genomic regions encompassing *hpn* and *hpn-2* highlighted several chromosomal inversions of a gene cluster composed of 4 genes (cluster 1 in Fig 3). Depending on the orientation of this gene cluster, *hpn* is either neighbor to *hpn-2*, with both genes located on the same strand, or *hpn* and *hpn-2* surround this cluster and are located on different strands. Some examples of DNA duplication associated with inversions have previously been reported in *H*. *pylori* [60], which could account for a putative mechanism explaining our observations. We consequently hypothesize that an inversion event has triggered the duplication of *hpn* that gave rise to its paralogue, *hpn-2*. In accordance with a more recent emergence of Hpn-2, following a duplication event, expansion of Gln residues occurred unequally in both the N and C-terminal regions of Hpn-2 in *H*. *pylori* and *H*. *acinonychis*, whereas Hpn has remained nearly unchanged. Because of the unusual amino acid composition of these proteins, the corresponding genes escaped currently used automated-annotation procedures and were not annotated in most *Helicobacter* genomes. Using a cutting edge technology, namely top-down proteomics, we confirmed the expression of these genes in several gastric *Helicobacter* species. Together with the *in vivo* essentiality of Hpn and Hpn-2, our observations indicate that the acquisition of Hpn in the common ancestor of gastric *Helicobacter* has been a decisive step for their adaptation to the human stomach, a niche that no other bacterium colonizes and in which metals are more soluble than in the intestine. Finally, we hypothesize that Hpn-2 provides an additional advantage for gastric colonization.

### Conclusion

Our data suggest that during evolution of the *Helicobacter* genus, acquisition of Hpn by gastric *Helicobacter* species was a decisive evolutionary event to allow *Helicobacter* to colonize the hostile gastric environment and was important for the emergence of one of the most successful bacterial pathogens, *H*. *pylori*.

Since no nickel containing proteins have been described in humans, nickel trafficking, and in particular Hpn and Hpn-2 proteins, constitute privileged targets for the development of novel anti-*Helicobacter* drugs [17]. In support of that, a pilot study has shown that, in *H*. *pylori*-infected patients, a nickel-free diet during one month enhanced the efficiency of the standard *Helicobacter* triple eradication therapy [61].

## Materials and Methods

### Ethics statement

Experiments in mice were carried out in strict accordance with the recommendations in the Specific Guide for the Care and the Use of Laboratory Animals of the Institut Pasteur, according to the European Directive (2010/63/UE) and the corresponding French law on animal experimentation (Arrêtés de 1988). The protocol has been approved by the Committee of Central Animal Facility Board of the Institut Pasteur. To follow the new European directives, the project was approved by the CETEA, Comité d’éthique en Expérimentation Animale of the Institut Pasteur (*#*2013–0051) and submitted for final approval to the Ministère de l’Enseignement Supérieur et de la recherche (#00317).

### Bacterial strains and growth conditions

Plasmids used to create or complement mutants of *H*. *pylori* were constructed and amplified using *Escherichia coli* strain MC1061 [62] (S3 Table) grown on solid or liquid Luria-Bertani medium [63] with spectinomycin 100 μg.mL^-1^, kanamycin 50 μg.mL^-1^ or chloramphenicol 30 μg.mL^-1^ for selection of transformants. The *E*. *coli* strain BTH101 [64] was used as a recipient for the Bacterial two hybrid analyses and BL21(DE3) (Novagen) for protein overexpression. The *H*. *pylori* strains used in this study (S3 Table) are derivatives of B128 [27,28], of X47-2AL [65] and of SS1 [66]. *H*. *pylori* strains were grown on Blood Agar Base 2 (Oxoid) plates supplemented with 10% defibrinated horse blood and with the following antibiotics-antifungal cocktail: amphotericin B 2.5 μg.ml^-1^, polymyxin B 0.31 μg.ml^-1^, trimethoprim 6.25 μg.ml^-1^ and vancomycin 12.5 μg.ml^-1^. For liquid cultures, we used Brucella broth (BD Difco) supplemented with 10% fetal calf serum (FCS, Eurobio) or with 0.2% β-cyclodextrin (Sigma) (designated here BBß), with the antibiotics-antifungal cocktail and the selective antibiotic when required.

Non-pylori gastric *Helicobacter* species were *Helicobacter acinonychis* (str. Sheeba) [45], *Helicobacter felis* (ATCC 49179) [67], *Helicobacter bizzozeronii* (CCUG35545) [68,69] and *Helicobacter mustelae* (12198) [70] and were grown in Brucella broth supplemented with 10% FCS for *H*. *acinonychis* and *H*. *mustelae* and with 20% FCS for *H*. *bizzozeronii* and *H*. *felis*. *Helicobacter* strains were grown at 37°C under microaerophilic atmosphere conditions (6% O_2_, 10% CO_2_, 84% N_2_) using an Anoxomat (MART Microbiology) atmosphere generator. Selection of *H*. *pylori* mutants was performed using kanamycin 20 μg.ml^-1^ or chloramphenicol 5 μg.ml^-1^, or both antibiotics when needed.

### Molecular techniques

Molecular biology experiments were performed according to standard procedures [71] and the supplier (Fermentas) recommendations. NucleoBond Xtra Midi Kit (Macherey-Nagel) and QIAamp DNA Mini Kit (Qiagen) were used for plasmid preparations and *H*. *pylori* genomic DNA extractions, respectively. PCR were performed either with Taq Core DNA polymerase (MP Biomedicals), or with Phusion Hot Start DNA polymerase (Finnzymes) when the product required high fidelity polymerase. The PCR8/GW/TOPO TA cloning kit (Invitrogen) was used to construct in *E*. *coli*, the *H*. *pylori* suicide plasmids that served for mutagenesis in *H*. *pylori*.

### Construction of *H*. *pylori* mutants

Chromosomal deletions of the entire genes encoding Hpn and Hpn-2 were performed in strains B128, X47-2AL and SS1. Despite strong conservation of these genes with their homologues from *H*. *pylori* strain 26695 (gene number *hp1432* for *hpn* and *hp1427* for *hpn-2* [26]), the corresponding regions in the genomes of strain B128, of its close derivative B8, strain X47-2AL and SS1 were surprisingly wrongly annotated [27,28,65] (for SS1, the sequence of the complete genome was kindly communicated to us by Dr. R. Ferrero). Deletions were introduced by allelic exchange using *H*. *pylori* suicide plasmids (see S3 Table) derived from PCR8/GW/TOPO in which about 600 bp of the 5’-end and the 3’-end regions immediately flanking the open reading frame of the target gene and an antibiotic resistance cassette (non-polar kanamycin cassette or non-polar chloramphenicol cassette [72]) were cloned using PCR fragments generated with the primers indicated in S4 Table. The mutants of *H*. *pylori* were obtained by natural transformation as described previously [73], with 1 μg of a preparation of the suicide plasmid DNA and selection on the corresponding antibiotic. The *∆hpn ∆hpn-2* double mutant was constructed in two steps: inactivation of *hpn* with the non-polar chloramphenicol cassette followed by the deletion of *hpn-2* and replacement with the non-polar kanamycin cassette. Deletion of the genes of interest and correct insertion of cassettes were verified by PCR and sequencing of the gene region.

To complement the ∆*hpn*::*kan* and ∆*hpn-2*::*kan* mutants in SS1 and B128 strains, we used a strategy described by Langford *et al* [74] to introduce the wild type *hpn* and *hpn-2* genes into the chromosome. This strategy was preferred to the classical plasmid-complementation to ensure that the wild type copies were not lost in the absence of selection pressure during mouse stomach colonization. This system uses the pIR203C04 suicide plasmid vector that contains fragments of an *H*. *pylori* strain 26695 intergenic region (*hp0203*–*hp0204*) flanking a chloramphenicol acetyltransferase cassette (*cat*) conferring chloramphenicol resistance and a multiple-cloning site. Insertion into this region has been shown by Langford *et al* [74] to affect neither *in vitro* growth nor mouse colonization. To ensure that the genes were actually transcribed, we first introduced the PureI promoter into the pIR203C04 plasmid by cloning a PCR fragment obtained by amplification from the chromosome of B128 using the couple of primer PureIUP/PureIDO and subsequent digestion with *Pst*I and *Bam*HI (for primers see S4 Table). Primer PureIDO introduced an *Eco*RV site at the 3’ end of the PureI promoter. The *hpn* and *hpn-2* genes were then amplified from the B128 chromosome (PCR using the couple of primer hpnUP/HpnDO or Hpn-2UP/Hpn-2DO) and cloned into the *EcoR*V/*Pst*I sites of the pIRC(PureI) plasmid, allowing the transcription of the genes from the PureI promoter. The p(PureI::hpn) and p(PureI::hpn-2) plasmids were used to transform strain 26695 selecting for chloramphenicol resistance. We verified the presence of the wild type copy of the *hpn* or *hpn-2* genes at the correct location by sequencing. In the figures, strains labeled as “+ pureI” are controls in which only the PureI promoter is inserted. The chromosomal DNAs from correct chloramphenicol resistant clones (*i*.*e*. where the wild type genes and the *cat* cassette integrated by homologous recombination between *hp0203* and *hp0204*) were then used to introduce the *hpn* and *hpn-2* genes into various strains by natural transformation and selection for chloramphenicol resistance.

### Mouse model of colonization

NMRI-specific pathogen-free mice (Charles River Laboratories) were orogastrically inoculated with 10^9^ CFU of *H*. *pylori* strains prepared in 100 μL of peptone broth. Several strains were tested (i) the wild type B128 parental strain and its isogenic ∆*hpn*, ∆*hpn-2* or ∆*hpn* ∆*hpn-2* mutants, (ii) the wild type X47-2AL strain and its isogenic ∆*hpn*, ∆*hpn-2* mutants and (iii) the wild type SS1 parental strain, its isogenic ∆*hpn*, ∆*hpn-2* or ∆*hpn* ∆*hpn-2* mutants and the two complemented strains ∆*hpn*/p(PureI::*hpn*) and ∆*hpn-2*/p(PureI::*hpn-2*). Each strain was used to inoculate a group of seven mice and the experiments were reproduced twice for SS1 and B128 strains and their mutants. As described in [72], one month after inoculation, mice were sacrificed and stomachs were crushed in peptone broth. Viable *H*. *pylori* colonizing the stomach were enumerated by culture of serial dilutions of homogenized tissue on blood agar plates containing bacitracin (200 μg.mL^-1^) and nalidixic acid (10 μg.mL^-1^).

### Measurement of metal sensitivity by disc diffusion and growth assays

*H*. *pylori* strains were tested for their sensitivity to nickel by the disc agar diffusion method. Therefore, 2.10^6^ CFU of each strain were prepared from liquid culture and were spread on plates containing Brucella broth, 1.5% bacto agar and 10% FCS. Sterile antibiotic assay discs (Whatman) were soaked in 4 μmol NiCl_2_ (Sigma) and placed in the center of the plate. The data correspond to at least three independent experiments with two replicates per experiment. Growth inhibition diameters (zones of inhibition, ZOI) were measured after 5 days of culture. For tests of metal sensitivity during growth in liquid, *H*. *pylori* cells were inoculated at OD_600_ 0.05 in 10 mL Brucella-Broth containing 10% FCS and increasing nickel concentrations (0, 5, 10, 30, 100 or 200 μM). Bacterial growth was monitored 17 hours later by measuring OD_600_. The MIC_50_ corresponds to the inhibitory nickel concentration that reduced growth by 50%.

### Nickel content measurements by Inductively Coupled Plasma Optical Emission Spectrometry (ICP-OES)

Overnight liquid cultures of *H*. *pylori* strain were grown until OD_600_ 0.9 at 37°C in 6 ml Brucella broth (pH adjusted to 7), then 200 μM NiCl_2_ were added and the cultures were left to grow until OD_600_ 6. Then, the 6 ml of culture were centrifuged at 4000 g at 4°C for 25 min through 400 μL of a 1:2 mixture of the silicone oils AR20/AR200 (Wacker) in order to separate the cells from the medium. Cells were lysed with 400 μL 0.2 M NaOH/1% SDS for 60 min at 95°C. Samples were calibrated by protein concentration measurements with the DC Protein Assay kit (BioRad). Then, the samples were mineralized overnight at 80°C with 300 μL of ultrapure 70% nitric acid (JT Baker) and diluted to 1/20 in ultrapure water. Nickel contents were measured by ICP-OES using a Vista MPX spectrometer (Varian). The content of Ni(II) was determined using a curve established with certified ICP grade standards. The measurement of each strain in each condition was performed in triplicates in at least three independent experiments.

### Urease activity measurements

Urease activity of whole *H*. *pylori* cells was tested by measuring the ammonia production using the Ammonia-Assay Kit (Sigma) as described [75]. *H*. *pylori* bacteria grown on blood agar plates for 24 hours were inoculated at OD_600_ 0.1 in BBß liquid medium and grown overnight. This preculture was used to inoculate the bacteria at OD_600_ 0.2 in BBß liquid medium without added nickel or supplemented with 10 μM of NiCl_2_. From this culture, log-phase bacteria (OD_600_ 0.5) were harvested and washed once with phosphate-buffered saline (Sigma). 5.10^7^ CFU of bacteria were resuspended in 1 mL of buffer (citric acid, 0.1M; Na_2_HPO_4_, 0.2 M pH 7) containing 5 mM urea. The data correspond to at least three independent experiments with two technical replicates each time. Aliquots were taken after 10 min of incubation at 37°C and centrifuged to pellet the bacteria. The NH_3_ concentration in the supernatant was measured immediately with the ammonia-assay kit according to the manufacturer’s (SIGMA) recommendations. This assay is based on the following reaction: in the presence of NH_3_, α-ketoglutaric acid and NADPH, the enzyme glutamate dehydrogenase produces glutamate and NADP^+^. The oxidation of NADPH to NADP^+^ results in a decrease in the absorbance at 340 nm that is proportional to the concentration of NH_3_. One unit (U) of urease activity was defined as the amount of enzyme that hydrolyzes 1 μmol urea per min per mg of total proteins.

### Purification of nickel-binding proteins

To identify the concentration range of imidazole required for the elution of Hpn and/or Hpn-2 after binding to the Ni-NTA agarose column, we first tested the procedure on *E*. *coli* BL-21 cells harboring plasmids overexpressing either Hpn or Hpn-2. To this aim, we constructed plasmids *p*RSFDuet1-*hpn* and *p*RSFDuet1*-hpn-2* (S3 and S4 Tables). pRSFDuet1-*hpn* is a derivative of vector pRSFDuet1 (Novagen) in which *hpn* was cloned into MCS1 between the *Nco*I and *Hin*dIII restriction sites. pRSFDuet1-*hpn-2* is a derivative of the same vector in which *hpn-2* was cloned into MCS2 between *Nde*I and *Kpn*I restriction sites. Both plasmids were transformed into *E*. *coli* BL-21 strain (Novagen) and transformants were selected on LB-Kanamycin. These strains were grown overnight in LB medium at 37°C and then diluted to OD_600_ 0.1 in 50 mL fresh LB medium containing the appropriate antibiotic. Cultures were grown to OD_600_ 0.6 and expression of Hpn or Hpn-2 proteins was induced by addition of 0.1 mM IPTG during 3 hours at 37°C. Cells were then harvested by centrifugation (10,000x*g*, 15 min, 4°C), washed in PBS, and resuspended in buffer 1 (Tris-HCl 25 mM, pH 7.5, containing NaCl 300 mM, Imidazole 5 mM, 2-mercaptoethanol 5 mM and proteases inhibitors). Both Hpn and Hpn-2 proteins remained bound on Ni-NTA agarose with concentrations of 100 mM imidazole and were eluted with 500 mM imidazole. Assuming that Hpn and Hpn-2 from the other gastric *Helicobacter* species would behave similarly, the same protocol was applied to extract the nickel-binding proteins from the five cultured gastric *Helicobacter* strains before their analysis by LC-MS/MS.

Gastric *Helicobacter* strains were grown in liquid to an OD_600_ of 1.5–2.0 and diluted to an OD_600_ of 0.05 (*H*. *pylori*, *H*. *acinonychis*, *H*. *mustelae*) or 0.2 (*H*. *bizzozeronii* and *H*. *felis*) in fresh Brucella broth. Cells were grown to an OD_600_ of 1.5 and recovered by centrifugation, washed in PBS, and resuspended in buffer 1. Then, cells were disrupted by sonication, and debris were removed by centrifugation (20,000x*g*, 30 min at 4°C). The supernatant was recovered was incubated 1h at 4°C with gentle shaking after addition of 0.5–1 mL of Ni-NTA agarose beads (Protino Ni-NTA agarose, Marcherey-Nagel). Extracts were then loaded onto 10 mL poly-prep chromatography columns (Bio Rad) and left at 4°C until all the beads pelleted. The beads were washed with 5 volumes of buffer 1, followed by a 5 volumes wash with buffer 2 (Tris-HCl 25 mM, pH 7.5, containing NaCl 300 mM, Imidazole 25 mM). Elution of Ni-binding proteins started with a 5-volume wash with buffer 3 (Tris-HCl 25 mM, pH 7.5, containing NaCl 300 mM, Imidazole 100 mM) in a first step, and then with a 3-volume wash with buffer 4 (Tris-HCl 25 mM, pH 7.5, containing imidazole 500 mM without NaCl or 2-mercaptoethanol). Aliquots of 0.5 mL were collected during the two last steps, and samples were kept at -20°C until use for LC-MS/MS analyzes. Presence of proteins was assessed by SDS-PAGE 14%.

### LC-MS/MS analysis

A nanoflow ultra-high pressure liquid chromatography Dionex Ultimate 3000 RSLCnano (Thermo Fisher) coupled to an Orbitrap Fusion Tribrid Mass Spectrometer (Thermo Fisher San Jose, USA) was used for all LC-MS/MS analyses. Fractions enriched in Ni-binding proteins were acidified with formic acid (~ 1% by volume) and loaded on a trap column (Dionex PepMap300 C_4_ 5 μm, 5 mm x 300 μm). Intact proteins were further separated on an Easy-Spray analytical column (Thermo Scientific PepSwift monolithic PS-DVB, 25 cm x 200 μm,). Solvent A consisted of 0.1% aqueous formic acid, solvent B consisted of 80% acetonitrile with 0.1% of formic acid. Sample loading was performed with a flow rate of 15 μL/min during 3 min, then a flow rate of 1 μL/min was used for protein separation on the analytical column. A 25 min (for targeted acquisitions) or 60 min (for data dependent analyses) gradient was used with the following conditions: from 1% to 50% solvent B in either 25 min or 60 min, then to 90% B in 1 min. Column compartment and analytical column were kept at 60°C. The "intact protein" mode was enabled on the Orbitrap Tribrid Fusion mass spectrometer. Tandem mass spectrometry experiments were performed with a resolution of 60,000 for MS^1^ and 120,000 MS^2^, averaging 3–6 microscans for MS^1^ and several microscans for MS^2^ (20 microscans for *H*. *mustelae* and *H*. *bizzozeronii* and 5–8 for the other strains). Fragmentation was performed either with HCD (collision energy 25 eV) or with ETD (8 ms reaction time, 2.0E5 reagent AGC target). The precursors were selected for fragmentation either in data-dependent mode (4 most intense ions, excluding charge states 1–5, 180 s dynamic exclusion) or in a targeted mode with an inclusion list of the theoretical mass-to-charge ratios of Hpn and Hpn-2 (with charge states between 7 and 13).

Data processing was accomplished using Thermo Scientific ProSightPC 3 software. Raw files were firstly deconvoluted with the Xtract algorithm. A three-stage database search was then performed for each strain against the corresponding Uniprot databases re-implemented with the sequences of Hpn and Hpn-2. The "absolute mass" mode was initially used with a precursor ion tolerance window of 4.08 Da (to account for potential S-S bonds) and 5 ppm for fragments. Then the "biomarker" mode was used with the same parameters to search for truncated proteoforms. Finally, an "absolute mass" search enlarging the precursor window to 10 kDa was used to search for all post-translationally modified proteoforms. Results are summarized in Table 2.

### Bacterial Two-Hybrid tests

The Bacterial Two-Hybrid (BACTH) test is based on the reconstitution of adenylate cyclase activity in a *cya* ^*-*^ *E*. *coli* strain as a result of the interaction between two proteins: a bait and a prey fused to two separate catalytic domains (T18 and T25) of the *Bordetella pertussis* adenylate cyclase. DNA inserts encoding the proteins of interest were obtained by PCR using the primers listed in S4 Table and chromosomal DNA from *H*. *pylori* B128 strain as a template. To detect interactions between the proteins of interest, several plasmids were constructed (S3 Table) expressing either a N-terminal or a C-terminal fusion of these proteins with the T25 catalytic domain (derived from vectors pKNT25 and pKT25, respectively) or either a N-terminal or a C-terminal fusion with the T18 catalytic domain (derived from vectors pUT18 and pUT18C, respectively). All insert were digested by *Eco*RI and *Pst*I then cloned into plasmids pUT18 and pKNT25 (PCR with primers UT18UP/UT18DO), pUT18C (PCR with primers UT18cUP/KT25DO) and pKT25 (PCR with primers KT25UP/KT25DO, S4 Table). The two plasmids expressing fusions to be tested were co-transformed in *E*. *coli* strain BTH101. The screening procedure involved growth of the transformants spread on Luria-Bertani agar plates containing kanamycin and ampicillin plus X-Gal with or without IPTG at a final concentration of 0.4% and 0.1 mM, respectively and incubated at 30°C for 2 days. Five mL of LB medium supplemented with antibiotics and IPTG 5x10^-4^M were inoculated with the transformants clones and incubated overnight at 30°C. Quantification of the interactions in strains carrying each plasmid combination was obtained by measurement of the β-galactosidase activity performed in 10 replicates as described in [64] (S2 Table).

Negative controls corresponded to strains with plasmids expressing one of the fusion proteins tested again the empty vector used to express the other partner (S2 Table). Positive controls consisted in a strain containing the plasmid used as a vector to construct the baits and the preys but carrying a GCN4 leucine zipper domain fused with the T18 or T25 domains (Zip-Zip construct in S2 Table).

### Immuno blotting

Overnight *H*. *pylori* B128 parental strain, *∆hpn*, *∆hpn-2* and *∆hpn ∆hpn-2* mutants grown in BBß were diluted to OD_600nm_ 0.2 in fresh BBß either without added nickel or with 10 μM NiCl_2_. Aliquots were taken after 4, 8 and 24 hours culture. Bacteria were pelleted and frozen. Immuno blotting was performed on crude extracts after cell lysis in 1% SDS pH 5 according to standard protocols. Protein amounts in the crude extracts were calibrated using the Bradford DC Protein Assay (Biorad) with bovine serum albumin (BSA) as a standard. 20 μg of total extracts were separated by 12.5% SDS-PAGE using the Criterion TGX Stain Free Any kD acrylamide gels (BioRad). Gels were blotted on a nitrocellulose membrane (GE healthcare). The *H*. *pylori* UreA protein was specifically detected with an anti-ureA rabbit polyclonal antibody used at a dilution of 1:5000 [76]. Goat anti-rabbit IgG-HRP (Santa Cruz) were used as secondary antibodies and the detection was achieved with the ECL reagent (Pierce). Band intensity was quantified with a Pharos imager using Image Quant Software (Molecular Dynamics).

### Bioinformatic analyses

The complete genome proteome sequences of 330 *Helicobacter* strains available at the NCBI (http://www.ncbi.nlm.nih.gov) in September 2014 were downloaded.

A massive all-against-all pairwise comparison of the proteins composing the 330 *Helicobacter* proteomes was performed with BLASTP [77]. Homologous sequences were clustered into protein families using SiLiX [78], a minimum thresholds of 80% length overlap and 30% amino acid identity. The 281 protein families present in a single copy in *Helicobacter* in at least 320 out the 330 proteomes were selected and aligned using MAFFT version 7 (option L-INS-I) [79]. The resulting alignments were trimmed with BMGE (default parameters) [80] and combined to build a large supermatrix (82,741 amino acid positions). The supermatrix was used to infer the phylogeny of the 330 strains with FastTree 2.1.7 [81], a WAG + Γ4 model. A maximum likelihood tree of a subsample of 100 strains was inferred with PhYML version 3.1 [82], the LG [83] + Γ4 model (estimated α parameter), and the NNI + SPR tree topology exploration strategy. The robustness of the resulting ML trees was assessed using the non-parametric bootstrap method implemented in PhYML (100 replicates of the original dataset).

Homologues of Hpn and Hpn-2 proteins and coding genes were searched in the 330 *Helicobacter* proteomes and genomes available using BLASTP and TBLASTN with default parameters, except the removal of the Low complexity regions filter and the matrix amino acid composition adjustment. There are a few *H*. *pylori* draft genomes *i*.*e*. strains Aklavik86 and PZ5086 in which *hpn* was not detected, and in strains Hp H5-b, E48, and PZ5024 in which *hpn-2* was not detected likely because the genomes are not complete.

Phylogenies of Hpn and Hpn-2 proteins were inferred with PhYML as described above. Tree topology tests were performed using the AU test [46] implemented in TreeFinder version 2011 [84]. The genomic regions surrounding the *hpn* and *hpn*-2 genes were investigated using EasyFig [85].

### Statistical analysis

The Student’s *t* test was used to determine significance of the means of the data. The Mann-Whitney test was used for mouse colonization assay to compare geometrical means of colonization loads.

## Supporting Information

S1 Fig Maximum Likelihood phylogeny of the *Helicobacter* core genome of the 330 strains analyzed. The tree was inferred with FastTree using a large supermatrix gathering the 281 single copy protein families present in at least 320 out of the 330 strains. Numbers at nodes represent SH-like supports computed with FastTree. The scale bar indicates the average number of substitution per site. The 100 strains in blue have been used for further analyses. Arrows indicate the likely origin of *hpn* and *hpn-2*.
(PDF)

S2 Fig Multiple alignment of the Hpn (A) and Hpn-2 (B) and Hpn-Hpn-2 (C) sequences found in the 330 *Helicobacter* strains. (DOCX)

S3 Fig Effect of nickel on the growth of *H*. *pylori* 26695 wild type strain and isogenic mutants measured with the nickel disk diffusion assay. (PDF)

S4 Fig Western blots of soluble proteins extracted from cultures of *H*. *pylori* B128 wild type strain and isogenic mutants in response to nickel supplementation of the medium (10 μM of NiCl_2_). Targeted protein is UreA. Each lane contains 20 μg proteins.
(PDF)

S1 Table List of the 330 *Helicobacter* strains analyzed during this study. The location of *hpn* and *hpn-2* in genomic sequences are indicated, as well as the accession number of the corresponding proteins when they were annotated in public databases. The phylogeny of these 330 strains is shown in S1 Fig. Strains in bold have been used for further phylogenetic analysis.
(XLSX)

S2 Table β-galactosidase activities (in miller units) of strains containing the pairs of plasmids indicated, measured in the presence of IPTG. (XLS)

S3 Table Bacteria and plasmids used in this study. (PDF)

S4 Table Primers used in this study. (PDF)
