# Supplementary figures and images for: Evolution of Helicobacter: Acquisition by Gastric Species of Two Histidine-Rich Proteins Essential for Colonization
Source: PLoS Pathog. 2015 Dec 7;11(12):e1005312. doi: 10.1371/journal.ppat.1005312 (PMC4671568; doi:10.1371/journal.ppat.1005312)

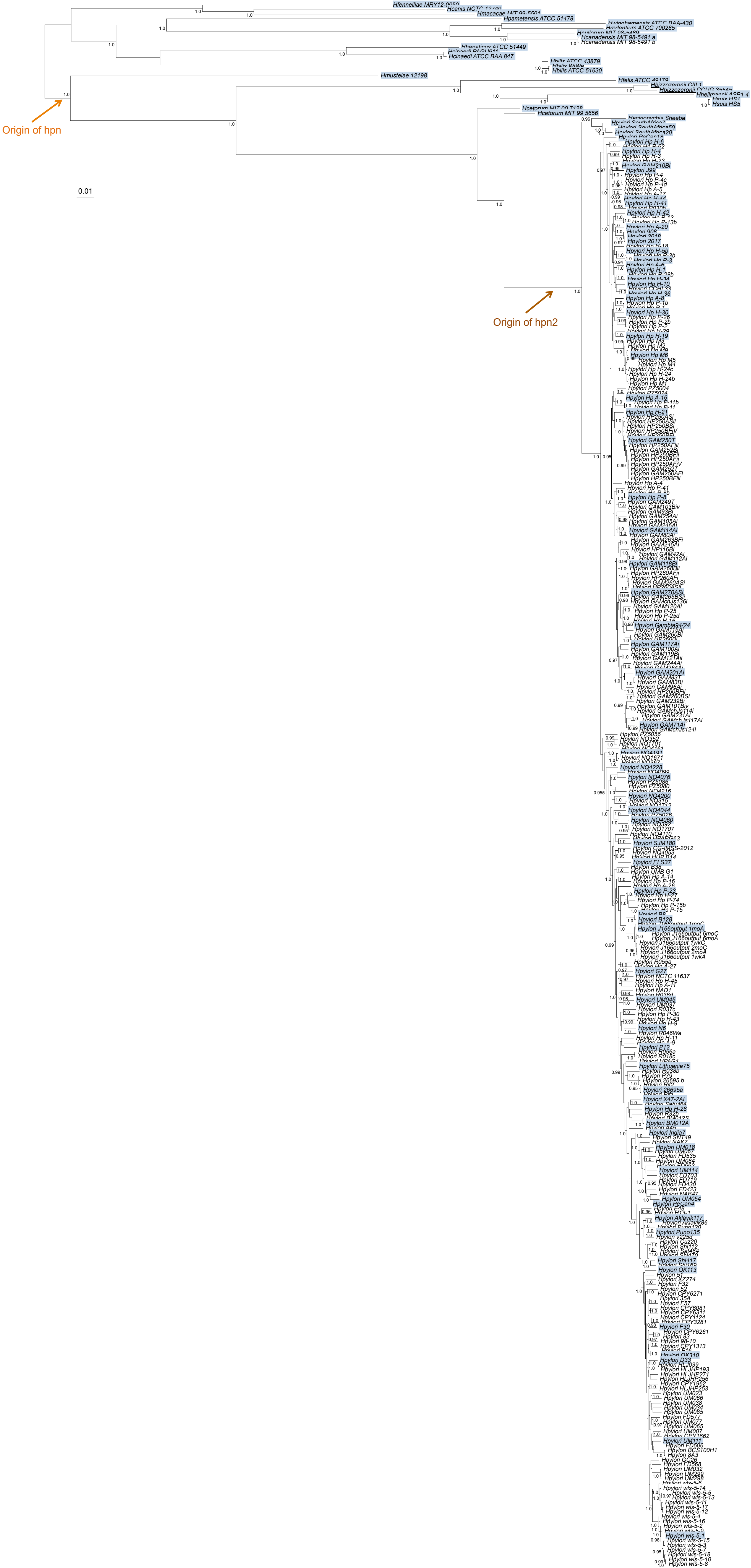

Supplement: S1 Fig — The tree was inferred with FastTree using a large supermatrix gathering the 281 single copy protein families present in at least 320 out of the 330 strains. Numbers at nodes represent SH-like supports computed with FastTree. The scale bar indicates the average number of substitution per site. The 100 strains in blue have been used for further analyses. Arrows indicate the likely origin of hpn and hpn-2. (PDF) [file ppat.1005312.s001.pdf]

Figure S3

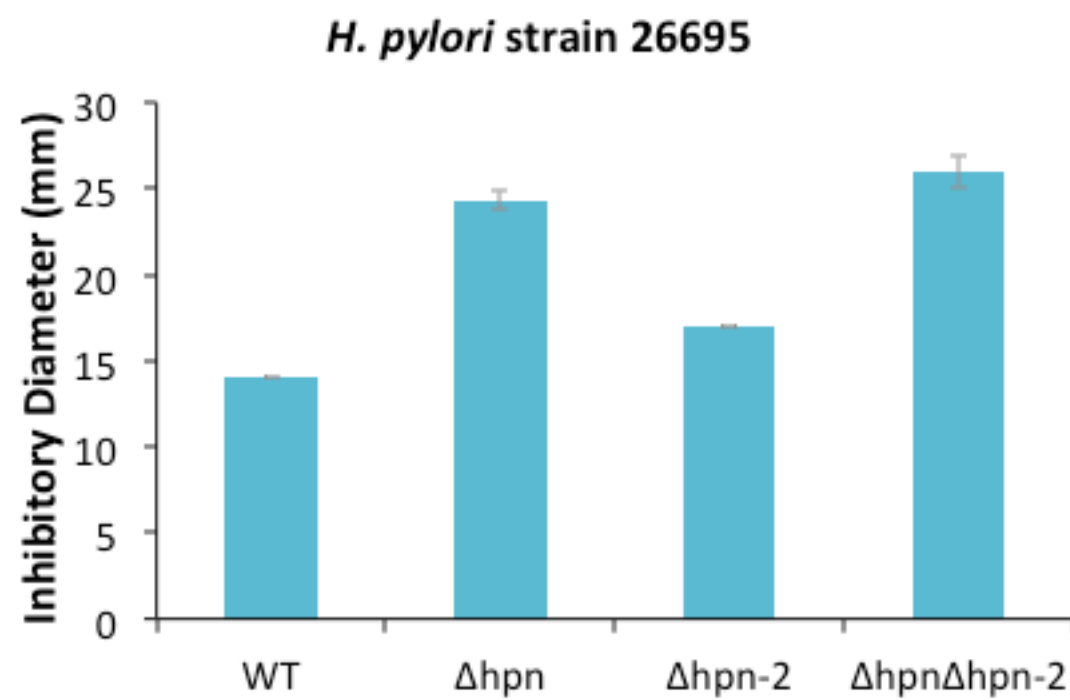

Supplement: S3 Fig — (PDF) [file ppat.1005312.s003.pdf]

Suppl Fig. 4

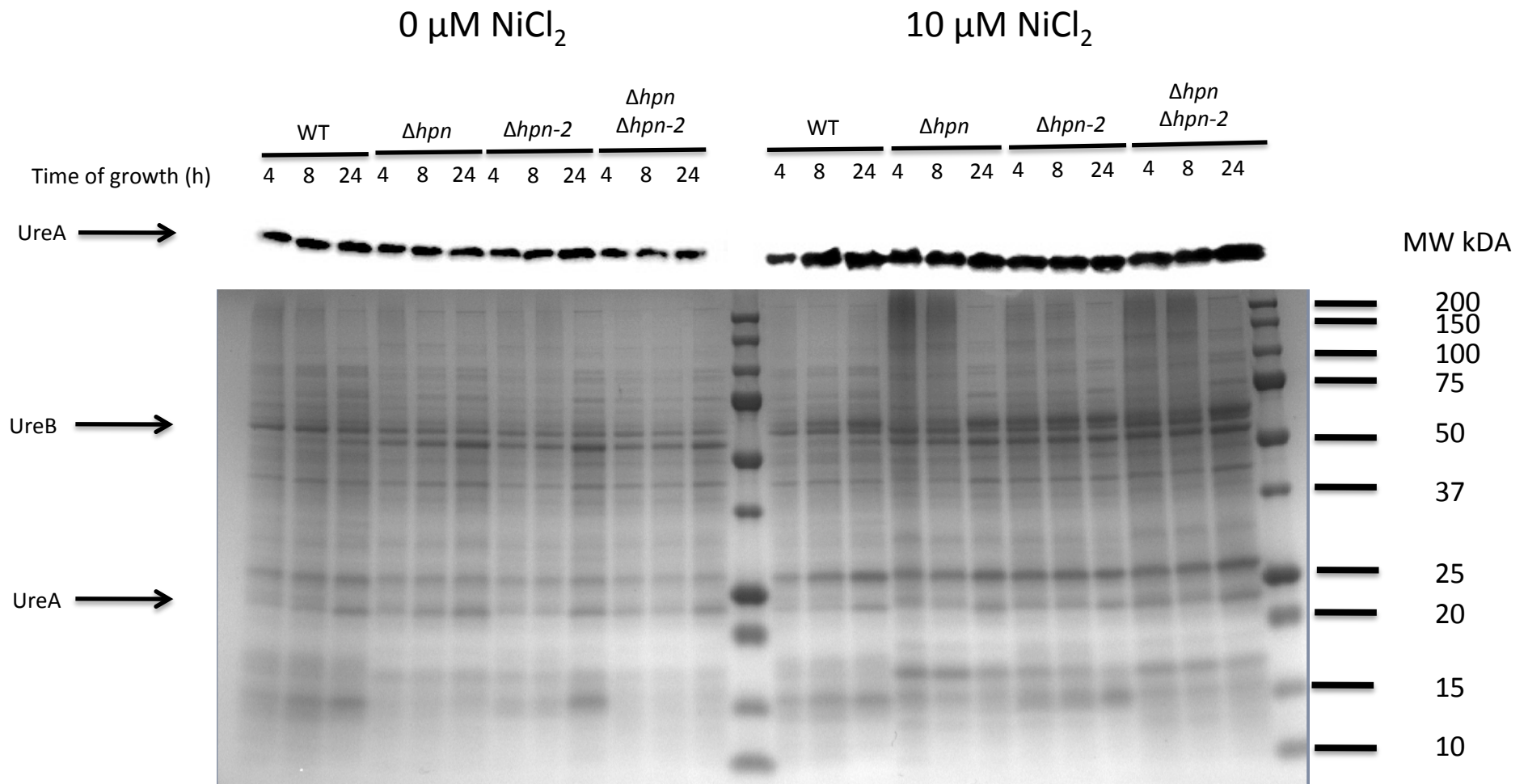

Supplement: S4 Fig — Targeted protein is UreA. Each lane contains 20 μg proteins. (PDF) [file ppat.1005312.s004.pdf]
