# Supplementary material for: Evolution of Helicobacter: Acquisition by Gastric Species of Two Histidine-Rich Proteins Essential for Colonization
Source: PLoS Pathog. 2015 Dec 7;11(12):e1005312. doi: 10.1371/journal.ppat.1005312 (PMC4671568; doi:10.1371/journal.ppat.1005312)
Supplement: S2 Fig — (DOCX) [file ppat.1005312.s002.docx]

**Suppl. Fig. S2A (Hpn multiple alignment)**

10 20 30 40 50 60 70 80 90

....|....|....|....|....|....|....|....|....|....|....|....|....|....|....|....|....|....|....|....

**Hpylori-GAM71Ai__459544270_rev** **------MAHHEEQHGGHHH------------HHHHTHHHHYHGGEHHH-HHHSSHHEE-------------GCCSASDSH--HQEEGCCHGHHE-----**

**Hpylori-GAM201Ai__459498646_fo** **------MAHHEEQHGGHHH------------HHHHTHHHHYHGGEHHH-HHHSSHHEE-------------GCCSTSDSH--HQEEGCCHGHHE-----**

**Hpylori-GAM117Ai__535930598_fo** **------MAHHEEQHGGHHH------------HHHHTHHHHYHGGEHHH-HHHSSHHEE-------------GCCSTSDSH--HQEEGCCHGHHE-----**

**Hpylori-GAM118Bi__459498783_re** **------MAHHEEQHGGHHH------------HHHHTHHHHYHGGEHHH-HHHSSHHEE-------------GCCSTSDSH--HQEEGCCHGHHE-----**

**Hpylori-GAM270ASi__459540604_f** **-------------------------------HHHHTHHHHYHGGEHHH-HHHSSHHEE-------------GCCSTSDSH--HQEEGCCHGHHE-----**

**Hpylori-GAM114Ai__459493577_fo** **------MAHHEEQHGGHHH------------HHHHTHHHHYHGGEHHH-HHHSSHHEE-------------GCCSTSDSH--HQEEGCCHGHHE-----**

**Hpylori-Hp-P-8__393100538_reve** **------MAHHEEQHGGHHH------------HHHHTHHHHYHGGEHHH-HHHSSHHEE-------------GCCSTSDSH--HQEEGCCHGHHE-----**

**Hpylori-Gambia94-24__317013424** **------MAHHEEQHGGHHH------------HHHHTHHHHYHGGEHHH-HHHSSHHEE-------------GCCSTSDSH--HQEEGCCHGHHE-----**

**Hpylori-Hp-H-21__393089925_for** **------MAHHEEQHGGHHH------------HHHHTHHHHYHGGEHHH-HHHSSHHEE-------------GCCSTSDSH--HQEEGCCHGHHE-----**

**Hpylori-GAM250T__459523467_rev** **------MAHHEEQHGGHHH------------HHHHTHHHHYHGGEHHH-HHHSSHHEE-------------GCCSTSDSH--HQEEGCCHGHHE-----**

**Hpylori-Hp-A-16__393072533_rev** **------MAHHEEQHGGHHH------------HHHHTHHHHYHGGEHHH-HHHSSHHEE-------------GCCSTSDSH--HQEECCCHGHHE-----**

**Hpylori-GAM210Bi__459506981_re** **------MAHHEEQHGGHHH------------HHHHTHHHHYHGGEHHH-HHHSSHHEE-------------GCCSTSDSH--HQEEGCCHGHHE-----**

**Hpylori-J99__12057207_reverse_** **------MAHHEEQHGGHHH------------HHHHTHHHHYHGGEHHH-HHHSSHHEE-------------GCCSTSDSH--HQEEGCCHGHHE-----**

**Hpylori-Hp-M6__393145710_rever** **------MAHHEEQHGG-HH------------HHHHTHHHHYHGGEHHH-HHHSSHHEE-------------GCCSTSDSH--HQEEGCCHGHHE-----**

**Hpylori-Hp-A-20__393045038_rev** **----------------------------------------------------SSHHEE-------------GCCSTSDSH--HQEEGCCHGHHE-----**

**Hpylori-2017__325996870_revers** **------MAHHEEQHGGHHH------------HHHHTHHHHYHGGEHHH-HHHSSHHEE-------------GCCSTSDSH--HQEEGCCHGHHE-----**

**Hpylori-2018__325995266_revers** **------MAHHEEQHGGHHH------------HHHHTHHHHYHGGEHHH-HHHSSHHEE-------------GCCSTSDSH--HQEEGCCHGHHE-----**

**Hpylori-908__307636682_reverse** **------MAHHEEQHGGHHH------------HHHHTHHHHYHGGEHHH-HHHSSHHEE-------------GCCSTSDSH--HQEEGCCHGHHE-----**

**Hpylori-Hp-P-13b__393129874_fo** **------MAHHEEQHGGHHH------------HHHHTHHHHYHGGEHHH-HHHSSHHEE-------------GCCSTSDSH--HQEEGCCHGHHE-----**

**Hpylori-Hp-H-34__393096911_rev** **------MAHHEEQHGGHHH------------HHHHTHHHHYHGGEHHH-HHHSSHHEE-------------GCCSTSDSH--HQEEGCCHGHHE-----**

**Hpylori-Hp-H-36__393060291_for** **------MAHHEEQHGGHHH------------HHHHTHHHHYHGGEHHH-HHHSSHHEE-------------GCCSTSDSH--HQEEGCCHGHHE-----**

**Hpylori-Hp-A-6__393066921_reve** **------MAHHEEQHGGHHH------------HHHHTHHHHYHGGEHHH-HHHSSHHEE-------------GCCSTSDSH--HQEEGCCHGHHE-----**

**Hpylori-Hp-H-1__471384880_reve** **------MAHHEEQHGGHHH------------HHHHTHHHHYHGGEHHH-HHHSSHHEE-------------GCCSTSDSH--HQEEGCCHGHHE-----**

**Hpylori-Hp-A-8__393067645_reve** **------MAHHEEQHGGHHH------------HHHHTHHHHYHGGEHHH-HHHSSHHEE-------------GCCSTSDSH--HQEEGCCHGHHE-----**

**Hpylori-Hp-H-41__393058399_rev** **------MAHHEEQHGGHHH------------HHHHTHHHHYHGGEHHH-HHHSSHHEE-------------GCCCTSDSH--HQEEGCCHGHHE-----**

**Hpylori-Hp-H-30__393054214_rev** **------MAHHEEQHGGHHH------------HHHHTHHHHYHGGEHHH-HHHSSHHEE-------------GCCSTSDSH--HQEEGCCHGHHE-----**

**Hpylori-Hp-H-10__393083611_rev** **------MAHHEEQHGGHHH------------HHHHTHHHHYHGGEHHH-HHHSSHHEE-------------GCCSTSDSH--HQEEGCCHGHHE-----**

**Hpylori-Hp-H-5b__393125211_rev** **------MAHHEEQHGGHHH------------HHHHTHHHHYHGGEHHH-HHHSSHHEE-------------GCCSTSDSH--HQEEGCCHGHHE-----**

**Hpylori-Hp-P-3__393098685_reve** **------MAHHEEQHGGHHH------------HHHHTHHHHYHGGEHHH-HHHSSHHEEGCCSTSDSHHQEEGCCSTSDSH--HQEEGCCHGHHE-----**

**Hpylori-Hp-H-19__393090315_rev** **------MAHHEEQHGGHHH------------HHHHTHHHHYHGGEHHH-HHHSSHHEE-------------GCCSTSDSH--HQEEGCCHGHHE-----**

**Hpylori-Hp-H-44__393062481_rev** **------MAHHEEQHGGHHH------------HHHHTHHHHYHGGEHHH-HHHSSHHEE-------------GCCSTSDSH--HQEEGCCHGHHE-----**

**Hpylori-Hp-H-6__393078985_reve** **------MAHHEEQHGGHHH------------HHHHTHHHHYHGGEHHH-HHHSSHHEE-------------GCCSTSDSH--HQEEGCCHGHHE-----**

**Hpylori-Hp-H-42__393059666_for** **------MAHHEEQHGGHHH------------HHHHTHHHHYHGGEHHH-HHHSSHHEE-------------GCCSTSDSH--HQEEGCCHGHHE-----**

**Hpylori-Hp-H-4__393077812_reve** **------MAHHEEQHGGHHH------------HHHHTHHHHYHGGEHHH-HHHNSHHEE-------------GCCSTSDSH--HQEEGCCHGHHE-----**

**Hpylori-PeCan18__384561342_for** **------MAHHEEQHGGHHH------------HHHHTHHHHYHGGEHHH-HHHSSHHEE-------------GCCSTSDSH--HQEEGCCHGHHE-----**

**Hpylori-NQ1671__327407074_forw** **------MAHHEEQHGGHHH------------HHHHTHHHHYHGGEHHH-HHHSSHHEE-------------GCCSTSDSH--HQEEGCCHGHHE-----**

**Hpylori-NQ4191__327407129_reve** **------MAHHEEQHGGHHH------------HHHHTHHHHYHGGEHHH-HHHSSHHEE-------------GCCSTSDSH--HQEEGCCHGHHE-----**

**Hpylori-NQ4228__393029926_reve** **------MAHHEEQHGGHHH------------HHHHTHHHHYHGGEHHH-HHHSSHHEE-------------GCCSTSDSH--HQEEGCCHGHHE-----**

**Hpylori-NQ4060__327406737_forw** **------MAHHEEQHGGHHH------------HHHHTHHHHYHGGEHHH-HHHSSHHEE-------------GCCSTSDSH--HQEEGCCHGHHE-----**

**Hpylori-NQ4200__393025980_forw** **------MAHHEEQHGGHHH------------HHHHTHHHHYHGGEHHH-HHHSSHHEE-------------GCCSTSDSH--HQEEGCCHGHHE-----**

**Hpylori-ELS37__380873809_rever** **------MAHHEEQHGGHHH------------HHHHTHHHHYHGGEHHH-HHHSSHHEE-------------GCCSTSDSH--HQEEGCCHGHHE-----**

**Hpylori-UM111__529185287_forwa** **------MAHHEEQHGGHHH------------HHHHTHHHHYHGGEHHH-HHHSSHHEE-------------GCCSSSDSH--HQEEGCCHGHHE-----**

**Hpylori-D33__657213133_forward** **------MAHHEEQHGGHHH------------HHHHTHHHHYHGGEHHH-HHHSSHHEE-------------GCCSTSDSH--HQEEGCCHGHHE-----**

**Hpylori-wls-5-14__594649680_re** **------MAHHEEQHGGHHH------------HHHHTHHHHYHGGEHHH-HHHSSHHEE-------------GCCSTSDSH--HQEEGCCHGHHE-----**

**Hpylori-wls-5-1__594682035_rev** **------MAHHEEQHGGHHH------------HHHHTHHHHYHGGEHHH-HHHSSHHEE-------------GCCSTSDSH--HQEEGCCHGHHE-----**

**Hpylori-OK310__459943864_rever** **------MAHHEEQHGGHHH------------HHHHTHHHHYHGGEHHH-HHHSSHHEE-------------GCCSTSDSH--HQEEGCCHGHHE-----**

**Hpylori-OK113__459942343_rever** **------MAHHEEQHGGHHH------------HHHHTHHHHYHGGEHHH-HHHSSHHEE-------------GCCSTSDSH--HQEEGCCHGHHE-----**

**Hpylori-F30__317178309_reverse** **------MAHHEEQHGGHHH------------HHHHTHHHHYHGGEHHH-HHHSSHHEE-------------GCCSTSDSH--HQEEGCCHGHHE-----**

**Hpylori-Shi417__384556702_reve** **------MAHHEEQHG-EHH------------HHHHTHHHHYHGGEHHH-HHHSSHHEE-------------GCCSTSDSH--HQEEGCCHGHHE-----**

**Hpylori-Puno135__344335753_rev** **------MAHHEEQHGGHHH------------HHHHTHHHHYHGGEHHH-HHHSSHHEE-------------GCCSTSDSH--HQEEGCCHGHHE-----**

**Hpylori-Aklavik117__425626953_** **------MAHHEEQHGGHHH------------HHHHTHHHHYHGGEHHH-HHHSSHHEE-------------GCCSASDSH--HQEEGCCHGHHE-----**

**Hpylori-PeCan4__308064373_reve** **------MAHHEEQHGGHHH------------HHHHTHHHHYHGGEHHH-HHHSSHHEE-------------GCCSTSDSH--HQEEGCCHGHHE-----**

**Hpylori-UM114__529216043_rever** **------MAHHEEQHGGHHH------------HHHHTHHHHYHGGEHHH-HHHSSHHEE-------------GCCSTSDSH--HQEEGCCHGHHE-----**

**Hpylori-UM054__507146682_rever** **------MAHHEEQHGGHHH------------HHHHTHHHHYHGGEHHH-HHHSSHHEE-------------GCCSTSDSH--HQEEGCCHGHHE-----**

**Hpylori-UM018__507146652_forwa** **------MAHHEEQHGGHHH------------HHHHTHHHHYHGGEHHH-HHHSSHHEE-------------GCCSTSDSH--HQEEGCCHGHHE-----**

**Hpylori-India7__317008680_forw** **------MAHHEEQHGGHHH------------HHHHTHHHHYHGGEHHH-HHHSSHHEE-------------GCCSTSDSH--HQEEGCCHGHHE-----**

**Hpylori-BM012A__558757555_forw** **------MAHHEEQHGGHHH------------HHHHTHHHHYHGGEHHH-HHHSSHHEE-------------GCCSTSDSH--HQEECCCHGHHE-----**

**Hpylori-Hp-H-28__393051219_rev** **------MAHHEEQHGGHHH------------HHHHTHHHHYHGGEHHH-HHHSSHHEE-------------GCCSTSDSH--HQEEGCCHGHHE-----**

**Hpylori-X47-2AL__558552313_for** **------MAHHEEQHGGHHH------------HHHHTHHHHYHGGEHHH-HHHSSHHEE-------------GCCSTSDSH--HQEEGCCHGHHE-----**

**Hpylori-Lithuania75__317011857** **------MAHHEEQHGGHHH------------HHHHTHHHHYHGGEHHH-HHHSSHHEE-------------GCCSTSDSH--HQEEGCCHGHHE-----**

**Hpylori-26695-a__409893163_rev** **------MAHHEEQHGGHHH------------HHHHTHHHHYHGGEHHH-HHHSSHHEE-------------GCCSTSDSH--HQEEGCCHGHHE-----**

**Hpylori-N6__384527583_forward_** **------MAHHEEQHGGHHH------------HHHHTHHHHYHGGEHHH-HHHSSHHEE-------------GCCSTSDSH--HQEEGCCHGHHE-----**

**Hpylori-P12__210132169_reverse** **------MAHHEEQHGGEHH------------HHHHTHHHHYHGGEHHH-HHHSSHHEE-------------GCCSTSDSH--HQEEGCCHGHHE-----**

**Hpylori-G27__208431905_reverse** **------MAHHEEQHGGHHH------------HHHHTHHHHYHGGEHHH-HHHSSHHEE-------------GCCSASDSH--HQEEGCCHGHHE-----**

**Hpylori-B128__216946188_revers** **------MAHHEEQHGGHHH------------HHHHTHHHHYHGGEHHH-HHHSSHHEE-------------GCCSTSDSH--HQEEGCCHGHHE-----**

**Hpylori-B8__298354685_forward_** **------MAHHEEQHGGHHH------------HHHHTHHHHYHGGEHHH-HHHSSHHEE-------------GCCSTSDSH--HQEEGCCHGHHE-----**

**Hpylori-J166output-1moA__63328** **------MAHHEEQHGGHHH------------HHHHTHHHHYHGGEHHH-HHHSSHHEE-------------GCCCTSDSH--HQEEGCCHGHHE-----**

**Hpylori-Hp-P-23__393110378_rev** **------MAHHEEQHGGHHH------------HHHHTHHHHYHGGEHHH-HHHSSHHEE-------------GCCSTSDSH--HQEEGCCHGHHE-----**

**Hpylori-UM045__507146963_rever** **------MAHHEEQHGGHHH------------HHHHTHHHHYHGGEHHH-HHHSSHHEE-------------GCCSTSDSH--HQEEGCCHGHHE-----**

**Hpylori-NQ4076__393031057_reve** **------MAHHEEQHGGHHH------------HHHHTHHHHYHGGEHHH-HHHSSHHEE-------------GCCSTSDSH--HQEEGCCHGHHE-----**

**Hpylori-NQ4099__393029120_forw** **------MAHHEEQHGGHHH------------HHHHTHHHHYHGGEHHHHHHHSSHHEE-------------GCCSTSDSH--HQEEGCCHGHHE-----**

**Hpylori-NQ4044__393030900_reve** **------MAHHEEQHGGHHH------------HHHHTHHHHYHGGEHHH-HHHSSHHEE-------------GCCSTSDSH--HQEEGCCHGHHE-----**

**Hpylori-SJM180__308059716_reve** **------MAHHEEQHGGHHH------------HHHHTHHHHYHGGEHHH-HHHSSHHEE-------------GCCSTSDSH--HQEEGCCHGHHE-----**

**Hpylori-SouthAfrica20__5321058** **------MAHHEEQHGGHHH------------HHHHTHHHHYHGGEHHH-HHHSSHHEE-------------GCCSSSNSH--HQEEGCCHGHHE-----**

**Hpylori-SouthAfrica50__5310740** **------MAHHEEQHGGHHH------------HHHHTHHHHYHGGEHHH-HHHSSHHEE-------------GCCSSSNSH--HQEEGCCHGHHE-----**

**Hpylori-SouthAfrica7__31701028** **------MAHHEEQHGGHHH------------HHHHTHHHHYHGGEHHH-HHHSSHHEE-------------GCCSSSNSH--HQEEGCCHGHHE-----**

**Hacinonychis-str.-Sheeba__1097** **------MAHHEEKHGEHH-------------HHHHTHHHHYHGGEHHHHHHHGSHHEE-------------GCCNTHHEQ--H-EDGCCHGHHE-----**

**Hcetorum-MIT-00-7128__38455496** **------MAHH-EHHGEHHH------------HHQHHHHHHYHGGEHHH-HHHHYSGGE-------------------EHHGEHHEEGCCHGHHGHHEED**

**Hcetorum-MIT-99-5656__38455327** **------MSGHKEHHGEHHH------------HHSHTHHHHYHGGEHHH-HHHSSHHEE-------------NCCHDGKHHGEHHEEGCCHGHHGHHE--**

**Hsuis-HS5__321146766_forward_2** **------MSHHTDECCHEHE--------ECAEHHHHHHHHHYYGGHHHH-HHH--HH---------------G-----EHH--HH-----HEHEEEHN--**

**Hsuis-HS1__321148481_forward_3** **------MSHHTDECCHEHE--------ECAEHHHHHHHHHYYGGHHHH-HHH--HH---------------G-----EHH--HH-----HEHEE--EHN**

**Hheilmannii-ASB1.4__408906187_** **------MA---DECCHDHNEGGCCHDHEGHGDHHHHHHHHYYGGHHHH-HHH--HH---------------GG--------------------------**

**Hbizzozeronii-CCUG-35545__3753** **------MSGHSEECCHGHE--------NECSEHHHHHHHHYHGGTHHH-HHH--HHGE-------------------HHH--HHHDGDHHHHEG-----**

**Hbizzozeronii-CIII-1__33533225** **------MSGHSEECCHGHE--------NECSEHHHHHHHHYHGGTHHH-HHH--HHGE-------------------HHH--HHHDGDHHHHEG-----**

**Hfelis-ATCC-49179__326319845_f** **------MSEHG-ECCHGHE-------GHEGHGVHHHHHHHYHGGTHHH-HHH--HH---------------G--GEHHHH-HHNQHGEHGSN------**

**Hmustelae-12198__290963437_rev** **------MNFSTNGKHNGEHH------HGEHHHGEHHHGEHHHGEHHHGEHHHGEHHH--------------GEHHHGEHHHGEHHHGEHHHGK----**

**Suppl. Fig. S2B (Hpn-2 multiple alignment)**

10 20 30 40 50 60 70 80 90 100

....|....|....|....|....|....|....|....|....|....|....|....|....|....|....|....|....|....|....|....|...

**Hpylori GAM71Ai__459544270_rev** **MAHHE----QQHQA-----QQQQQQANSQHHHHHHAHHHHYYGGEHHHHNAQQHAEQQAEQQAQQ------------------------QQQQQAHQQNQQY-**

**Hpylori GAM201Ai__459498646_re** **MAHHE----QQHQA----QQQQQQQANSQHHHHHHAHHHHYYGGEHHHHNAQQHAEQQAEQQAQQQ----------------QQQQAHQQQQQKAQQQNQQY-**

**Hpylori GAM117Ai__535930598_fo** **MAHHE----QQHQA----QQQQQQQANSQHHHHHHAHHHHYYGGEHHHHNAQQHAEQQAEQHAQQ------------------------QQQQQAQQQNQQY-**

**Hpylori GAM118Bi__459498783_re** **MAHHE--------------QQQQQQANSQHHHHHHAHHHHYYGGEHHHHNAQQHAEQQAEQHAQQQ----------------QQQQAH-QQQQKAQQQNQQY-**

**Hpylori GAM270ASi__459540653_f** **MAHHE----QQHQA----QQQQQQQANSQHHHHHHTHHHHYYGGEHHHHNAQQHAEQQAEQHAQQQ----------------QQQQAHQQQQQKAQQQNQQY-**

**Hpylori GAM114Ai__459493577_fo** **MAHHE----QQHQA----QQQQQQQANSQHHHHHHEHHHHYYGGEHHHHNAQQHAEQQAEQHAQQQ----------------QQQQAHQQQQQKAQQ*NQQY-**

**Hpylori Hp P-8__393100538_reve** **MAHHE----QQHQA----QQQQQQQANSQHHHHHHAHHHHYYGGEHHHHNAQQHAEQQAEQHAQQ------------------------QQQQKAQQQNQQY-**

**Hpylori Gambia94/24__317013424** **MAHHE----QQHQA-----QQQQQQANS---HHHHVHHHHYYGGEHHHHNAQQHAEQQAEQHAQQ------------------------QQQQQAHQQNQQY-**

**Hpylori Hp H-21__393089925_for** **MAHHE----QQHQA----QQQQQQQANSQHHHHHHAHHHHYYGGEHHHHNAQQHAEQQAEQHAQQQ----------------QQQQAHQQQQQKAQQQNQQY-**

**Hpylori GAM250T__459523467_rev** **MAHHE----QQHQV----QQQQQQQANSQHHHHHHAHHHHYYGGEHHHHNAQQHAEQQAEQHAQQQ----------------QQQQAHQQQQQKAQQQNQQY-**

**Hpylori Hp A-16__393072533_rev** **MAHHE----QQHQA----QQQQQQQANSQHHHHHHAHHHHYYGGEHHHHNAQQHAEQQAEQHAQQ------------------------QQQQKAQQQNQQY-**

**Hpylori GAM210Bi__459506981_re** **MAHHE----QQHQA-----QQQQQQANSQHHHHHHAHHHHYYGGEHHHHNAQQHAEQQAEQHVQQQ----------------QQQQAHQQQQQKAQQQNQQY-**

**Hpylori J99__12057207_reverse_** **MAHHE----QQHQA-----QQQQQQANSQHHHHHHVHHHHYYGGEHHHHNAQQHAEQQAEQHAQQQ----------------QQQQAHQQQQQKAQQQNQQY-**

**Hpylori Hp M6__393145710_rever** **MAHHE----QQHQA----QQQQQQQANSQHHHHHHVHHHHYYGGEHHHHNAQQHAEQQAEQHAQQQ----------------QQQQAHQQQQQKAQQQNQQY-**

**Hpylori Hp A-20__393045038_rev** **MAHHE----QQHQA----QQQQQQQANSQHHHHHHAHHHHYYGGEHHHHNAQQHAEQQAEQHAQQQ----------------QQQQAHQQQQQKAQQQNQQY-**

**Hpylori 2017__325996870_revers** **MAHHE----QQHQV-----QQQQQQANSQHHHHHHAHHHHYYGGEHHHHNAQQHAEQQAEQHAQQ------------------------QQQQKAQQQNQQY-**

**Hpylori 2018__325995266_revers** **MAHHE----QQHQV-----QQQQQQANSQHHHHHHAHHHHYYGGEHHHHNAQQHAEQQAEQHAQQ------------------------QQQQKAQQQNQQY-**

**Hpylori 908__307636682_reverse** **MAHHE----QQHQV-----QQQQQQANSQHHHHHHAHHHHYYGGEHHHHNAQQHAEQQAEQHAQQ------------------------QQQQKAQQQNQQY-**

**Hpylori Hp P-13b__393129874_fo** **MAHHE----QQHQA-----QQQQQQANSQHHHHHHAHHHHYYGGEHHHHNAQQHAEQQAEQQAQQQ----------------QQQQAHQQQQQKAQQQNQQY-**

**Hpylori Hp H-34__393096911_rev** **MAHHE-------------QQQQQQQANSQHHHHHHAHHHHYYGGEHHHHNVQQHAEQQAEQHAQQ------------------------QQQQQAHQQNQQY-**

**Hpylori Hp H-36__393060291_for** **MAHHE----QQHQA----QQQQQQQANSQHHHHHHAHHHHYYGGEHHHHNVQQHAEQQAEQHAQQ------------------------QQQQQAHQQNQQY-**

**Hpylori Hp A-6 HpA__393066921_** **MAHHE----QQHQA-------------------------------------------------Q------------------------QQQQQKAQQQNQQY-**

**Hpylori Hp H-1__471384880_reve** **MAHHE----QQHQA----QQQQQQQANSQHHHHHHAHHHHYYGGEHHHHNAQQHAEQQAEQHAQQQ----------------QQQQAHQQQQQKAQQQQQQY-**

**Hpylori Hp A-8__393067645_reve** **MAHHE----QQHQA----QQQQQQQANSQHHYHHHAHHHHYYGGEHHHHNVQQHAEQQAEQHAQQQ----------------QRQQAHQ--------------**

**Hpylori Hp H-41__393058399_rev** **MAHHE----QQHQA-----QQQQQQANSQHHHHHHVHHHHYYGGEHHHHNAQQHAEQQAEQHAQQQ----------------QQQQAHQQQQQKAQQQNQQY-**

**Hpylori Hp H-30__393054214_rev** **MAHHE----QQHQA------QQQQQANSQHHHHHHAHHHHYYGGEHHHHNAQQHAEQQAEQHAQQ------------------------QQQQKAQQQNQQY-**

**Hpylori Hp H-10__393083611_rev** **MAHHE----QQHQA----QQQQQQQANSQHHHHHHAHHHHYYGGEHHHHNAQQHAEQQAEQHAQQ------------------------QQQQKAQQQNQQY-**

**Hpylori Hp P-3__393098685_reve** **MAHHE----QQHQA-----QQQQQQANSQHHHHHHAHHHHYYGGEHHHHNAQQHAEQQAEQHAQQ------------------------QQQQKAQQQNQQY-**

**Hpylori Hp H-19__393090315_rev** **MAHHE----QQHQA-------------------------------------------------Q------------------------QQQQQKAQQQNQQY-**

**Hpylori Hp H-44__393062481_rev** **MAHHE----QQHQA-------------------------------------------------Q------------------------QQQQQKAQQQNQQY-**

**Hpylori Hp H-6__393078985_reve** **MAHHE----QQHQA----QQQQQQQANSQ-HHHHHTHHHHYYGGEHHHHNAQQHAEQQAEQHAQQQ----------------QQQQAHQQQQQKAQQQNQQY-**

**Hpylori Hp H-42__393059666_for** **MAHHE----QQHQA----QQQQQQQANSQHHHHHHAHHHHYYGGEHHHHNAQQHAEQQAEQHAQQQ-----------------------QQQQKAQQQNQQY-**

**Hpylori Hp H-4__393077812_reve** **MAHHE----QQHQAQQQQQQQQQQQANSQHHHHHHAHHHHYYGGEHHHHNAQQHAEQQAEQHVQQ-----------------QAHQ---QQQQKAQQQNQQY-**

**Hpylori PeCan18__384561342_for** **MAHHE----QQHQA----QQQQQQQANSQHHHHHHAHHHHYYGGEHHHHNAQQHAEQQAEQHAQQQ----------------QQQQAHQQQQQKAQQQNQQY-**

**Hpylori NQ1671__327407074_forw** **MAHHE----QQHQA----QQQQQQQANSQHHHHHHAHHHHYYGGEHHHHNAQQHAEQQAEQHAQQ------------------------QQQQKAQQQNQQY-**

**Hpylori NQ4191__327407129_reve** **MAHHE----QQHQA----QQQQQQQANSQHHHHHHAHHHHYYGGEHHHHNAQQHAEQQAEQHAQQ------------------------QQQQKAQQQNQQY-**

**Hpylori NQ4228__393029926_reve** **MAHHE----------------QQQQANSQHHHHHHAHHHHYYGGEHHHHNAQQQAEQQAEQQAQQ------------------------QQQQKAQQQNQQY-**

**Hpylori NQ4060__327406737_forw** **MAHHE---------------QQQQQANSQHHHHHHAHHHHYYGGEHHHHNAQQHAEQQAEQQAQQQ----------------QQQQAHQQQQQKAQQQNQQY-**

**Hpylori NQ4200__393025980_reve** **MAHHE----QQHQA----QQQQQQQANSQHHHHHHAHHHHYYGGEHHHHNAQQHAEQQAEQQAQQ------------------------QNQQKAQQQNQQY-**

**Hpylori ELS37__380873809_rever** **MAHHE-------------QQQQQQQANSQHHHHHHAHHHHYYGGEHHHHNAQQHAEQQAEQQAQQQ-----------------------QQQQKAQQQNQQY-**

**Hpylori UM111__529185287_rever** **MAHHE----------------QQQQANSQHHHHHHAHHHHYYGGEHHHHNAQQHAEQQAEQQAQQQ----------------QQQKAQQQQQQQAQQQNQQY-**

**Hpylori D33__657213133_reverse** **MAHHE----------------QQQQANSQHHHHHHEHHHHYYGGEHHHHNAQQHAEQQAEQQAQQQ----------------QQQKAQQQQQQKAQQQNQQY-**

**Hpylori wls-5-14__594649680_fo** **MAHHE-----------------QQQANSQHHHHHHAHHHHYYGGEHHHHNAQQQAEQQAEQQAEQQ----------------AQQQAQQQQQQKAQQQNQQY-**

**Hpylori wls-5-1__594682035_for** **MAHHE----------------QQQQANSQHHHHHHEHHHHYYGGEHHHHNAQQQAEQQAEQQAQQQ----------------QQQQAHQQQQQKAQQQNQQY-**

**Hpylori OK310__459943864_forwa** **MAHHE--------------QQQQQQANSQHHHHHHAHHHHYYGGEHHHHNVQQHAEQQAEQQA--------------------------QQQQKAQQQNQQY-**

**Hpylori OK113__459942343_forwa** **MAHHE--------------QQQQQQANSQHHHHHHAHHHHYYGGEHHHHNAQQHAEQQAEQQAQQ------------------------QQQQKAQQQNQQY-**

**Hpylori F30__317178309_forward** **MAHHE---------------QQQQQANSQHHHHHHAHHHHYYGGEHHHHNVQQHAEQQAEQQAQQQ----------------AQQQAHQQQQQKAQQQNQQY-**

**Hpylori Shi417__384556702_forw** **MAHQQ-------QA----QQQQQQQANSHHHHHHHAHHHHYYGGEHHHHNAQQHAEQQAEQQAQQQ----------------QQQQAHQQQQQKAQQQNQQY-**

**Hpylori Puno135__344335753_for** **MAH------------------QQQQANSQHHHHHHAHHHHYYGGEHHHHNAQQHAEQQAEQQVQQQ--------------------AQQQQQQQAQQQNQQY-**

**Hpylori Aklavik117__425626953_** **MAHHE----------------QQQQANSQHHHHHHAHHHHYYGGEHHHHNAQQHAEQQAEQKAQQQ----------------QQQKAQQ--------------**

**Hpylori PeCan4__308064373_forw** **MAHHE-------------QQQQQQQANSQHHHHHHAHHHHYYGGEHHHHNAQQHAEQQAEQQAQQQ----------------QQQQAHQQQQQKAQQQNQQY-**

**Hpylori UM114__529216043_forwa** **MAHHE----QQHQA----QQQQQQQANSQ-HHHHHVHHHHYYGGEHHHHNAQQHAEQQAEQQAHQ------------------------QQQQKAQQQNQQY-**

**Hpylori UM054__507146682_forwa** **MAHHE----QQHQA-----QQQQQQANSQHHHHHHAHHHHYYGGEHHHHNAEQHAEQQAEQQAQQQ----------------QQQQAHQQQQQKAQQQNQQY-**

**Hpylori UM018__507146652_rever** **MAHHE----QQHQA-----QQQQQQANSQHHHHHHVHHHHYYGGEHHHHNAQQHAEQQAEQQAQQQ----------------QQQQAHQQQQQKAQQQNQQY-**

**Hpylori India7__317008680_reve** **MAHHE----QQHQA----QQQQQQQANSQHHHHHHAHHHHYYGGEHHHHNAQQHAEQQAEQQAQQQ-----------------------QQQQKAQQQNQQY-**

**Hpylori BM012A__558757555_reve** **MAHHE--------------QQQQQQANSQHHHHHHAHHHHYYGGEHHHHNAQQHAEQQAEQQAQQQ-----------------------QQQQKAQQQNQQY-**

**Hpylori Hp H-28__393051219_for** **MAHHE--------------QQQQQQANSQHHHHHHAHHHHYYGGEHHHHNAQQHAEQQAEQQAQQ------------------------QQQQKAQQQNQQY-**

**Hpylori X47-2AL__558552313_rev** **MAHHE--------------QQQQQQANSQHHHHHHAHHHHYYGGEHHHHNAQQHAEQQAEQQAQQQ----------------QQQQAQQQQQQKAQQQNQQY-**

**Hpylori Lithuania75__317011857** **MAHHE-------------QQQQQQQANSQHHHHHHAHHHHYYGGEHHHHNAEQHAEQQAEQQAEQQAQQHAEQQ--------AEQQAQQQQQQKAQQQNQQY-**

**Hpylori 26695a__409893163_forw** **MAHHE--------------QQQQQQANSQHHHHHHAHHHHYYGGEHHHHNAQQHAEQQAEQQAQQHX---------------QQQQAHQQQQQKAQQQNQQYX**

**Hpylori P12__210132169_forward** **MAHHE----QQHQA----QQQQQQQANSQHHHHHHEHHHHYYGGEHHHHNAQQHAEQQAEQQAQQQ----------------QQQQAHQQQQQKVQQQNQQY-**

**Hpylori G27__208431905_forward** **MAHHE-------------QQQQQQQANSQHHHHHHAHHHHYYGGEHHHHNAQQHAEQQAEQQAQQQ----------------QQQQAHQQQQQKAQQQNQQY-**

**Hpylori B128__216946188_forwar** **MAHHE---------------QQQQQANSQHHHHHHAHHHHYYGGEHHHHNAQQHAEQQAEQQAQQQ---------------------QQQQQQQAQQQNQQY-**

**Hpylori B8__298354685_reverse_** **MAHHE---------------QQQQQANSQHHHHHHAHHHHYYGGEHHHHNAQQHAEQQAEQQAQQQ---------------------QQQQQQQAQQQNQQY-**

**Hpylori J166output 1moA__63328** **MAHHE---------------QQQQQANSQHHHHHHAHHHHYYGGEHHHHNAQQHAEQQAEQQAQQQ---------------------QQQQQQQAQQQNQQY-**

**Hpylori Hp P-23__393110378_for** **MAHHE----QQHQA-----QQQQQQANSQHHHHHHAHHHHYYGGEHHHHNAQQQAEQQAEQQAEQQAQQQ------------QQQQAHQQQQQKAQQQNQQY-**

**Hpylori UM045__507146963_forwa** **MAHHE----QQHQA-----QQQQQQANSQHHHHHHAHHHHYYGGEHHHHNAQQHAEQQAEQQAQQQ----------------QQQQAHQQQQQKAQQQNQQY-**

**Hpylori NQ4076__393031057_forw** **MAHHE--------------QQQQQQANSQHHHHHHAHHHHYYGGEHHHHNAQQHAEQQAEQQAQQQ----------------QQQQAHQQQQQKAQQQNQQY-**

**Hpylori NQ4099__393029120_reve** **MAHHE----QQHQV----QQQQQQQANSQHHHHHHAHHHHYYGGEHHHHNAQQHAEQQAEQHAEQQAEQQAQQQQQQQAQQQQQQQAQQQNQQKAQQQNQQY-**

**Hpylori NQ4044__393030900_forw** **MAHHE----QQHQA----QQQQQQQANSQHHHHHHAHHHHYYGGEHHHHNAQQHAEQQAEQQAQQQ----------------QQQQAHQQQQQKAQQQNQQY-**

**Hpylori SJM180__308059716_forw** **MAHHE----QQHQA----QQQQQQQANSQHHHHHHAHHHHYYGGEHHHHNAQQHAEQQAEQQAQQQ----------------QQQQAHQQQQQKAQQQNQQY-**

**Hpylori SouthAfrica20__5321058** **MAHHE----QQ--------QQAQQQANSQHHHHHHAHHHHYYGGEHHHHNAQQYAEQQAEQQAQQQ----------------QQQQAHQQQQQKAQQQNQQY-**

**Hpylori SouthAfrica50__5310740** **MAHHE------------QQQQAQQQANSQHHHHHHAHHHHYYGGEHHHHNAQQHAEQQAEQQAQQ------------------------QQQQKAQQQNQQY-**

**Hpylori SouthAfrica7__31701028** **MAHHE-------------QQQAQQQANSQHHHHHHAHHHHYYGGEHHHHNAQQYAEQQAEQQAEQQ----------------AQQ---QQQQQKAQQQNQQY-**

**Hacinonychis str. Sheeba__1097** **MANHEHNQQNQQQAQ--ACQQNQQQANGQ--HHHHEHHHHYYGGTHHHH---HHAEQHAEQQAN---------------------------QQARQQQNQQS-**

**Hpylori N6__384527583_reverse_** **MAHHE----QQHQA----QQQQQQQANSQHHHHHHEHHHHYYGGEHHHHNAQQHAEQQAEQQAQQQ----------------QQQQAHQQQQQKAQQQNQQY-**

**Suppl. Fig. S2C (Hpn - Hpn-2 multiple alignment)**

10 20 30 40 50 60 70 80 90 100 110 120

....|....|....|....|....|....|....|....|....|....|....|....|....|....|....|....|....|....|....|....|....|....|....|....|....|..

**Hpylori-GAM71Ai__459544270_rev** **------MAHHE----QQHQA-----QQQQQQANSQ----------HHHHHHAHHHHYYGGEHHH-HNAQQHAEQQAEQQAQQ------------------------QQ--QQQAHQQNQQY------**

**Hpylori-GAM201Ai__459498646_re** **------MAHHE----QQHQA----QQQQQQQANSQ----------HHHHHHAHHHHYYGGEHHH-HNAQQHAEQQAEQQAQQQ----------------QQQQAHQQQ--QQKAQQQNQQY------**

**Hpylori-GAM117Ai__535930598_fo** **------MAHHE----QQHQA----QQQQQQQANSQ----------HHHHHHAHHHHYYGGEHHH-HNAQQHAEQQAEQHAQQ------------------------QQ--QQQAQQQNQQY------**

**Hpylori-GAM118Bi__459498783_re** **------MAHHE--------------QQQQQQANSQ----------HHHHHHAHHHHYYGGEHHH-HNAQQHAEQQAEQHAQQQ----------------QQQQAH-QQ--QQKAQQQNQQY------**

**Hpylori-GAM270ASi__459540653_f** **------MAHHE----QQHQA----QQQQQQQANSQ----------HHHHHHTHHHHYYGGEHHH-HNAQQHAEQQAEQHAQQQ----------------QQQQAHQQQ--QQKAQQQNQQY------**

**Hpylori-GAM114Ai__459493577_fo** **------MAHHE----QQHQA----QQQQQQQANSQ----------HHHHHHEHHHHYYGGEHHH-HNAQQHAEQQAEQHAQQQ----------------QQQQAHQQQ--QQKAQQ*NQQY------**

**Hpylori-Hp-P-8__393100538_reve** **------MAHHE----QQHQA----QQQQQQQANSQ----------HHHHHHAHHHHYYGGEHHH-HNAQQHAEQQAEQHAQQ------------------------QQ--QQKAQQQNQQY------**

**Hpylori-Gambia94/24__317013424** **------MAHHE----QQHQA-----QQQQQQANS-------------HHHHVHHHHYYGGEHHH-HNAQQHAEQQAEQHAQQ------------------------QQ--QQQAHQQNQQY------**

**Hpylori-Hp-H-21__393089925_for** **------MAHHE----QQHQA----QQQQQQQANSQ----------HHHHHHAHHHHYYGGEHHH-HNAQQHAEQQAEQHAQQQ----------------QQQQAHQQQ--QQKAQQQNQQY------**

**Hpylori-GAM250T__459523467_rev** **------MAHHE----QQHQV----QQQQQQQANSQ----------HHHHHHAHHHHYYGGEHHH-HNAQQHAEQQAEQHAQQQ----------------QQQQAHQQQ--QQKAQQQNQQY------**

**Hpylori-Hp-A-16__393072533_rev** **------MAHHE----QQHQA----QQQQQQQANSQ----------HHHHHHAHHHHYYGGEHHH-HNAQQHAEQQAEQHAQQ------------------------QQ--QQKAQQQNQQY------**

**Hpylori-GAM210Bi__459506981_re** **------MAHHE----QQHQA-----QQQQQQANSQ----------HHHHHHAHHHHYYGGEHHH-HNAQQHAEQQAEQHVQQQ----------------QQQQAHQQQ--QQKAQQQNQQY------**

**Hpylori-J99__12057207_reverse_** **------MAHHE----QQHQA-----QQQQQQANSQ----------HHHHHHVHHHHYYGGEHHH-HNAQQHAEQQAEQHAQQQ----------------QQQQAHQQQ--QQKAQQQNQQY------**

**Hpylori-Hp-M6__393145710_rever** **------MAHHE----QQHQA----QQQQQQQANSQ----------HHHHHHVHHHHYYGGEHHH-HNAQQHAEQQAEQHAQQQ----------------QQQQAHQQQ--QQKAQQQNQQY------**

**Hpylori-Hp-A-20__393045038_rev** **------MAHHE----QQHQA----QQQQQQQANSQ----------HHHHHHAHHHHYYGGEHHH-HNAQQHAEQQAEQHAQQQ----------------QQQQAHQQQ--QQKAQQQNQQY------**

**Hpylori-2017__325996870_revers** **------MAHHE----QQHQV-----QQQQQQANSQ----------HHHHHHAHHHHYYGGEHHH-HNAQQHAEQQAEQHAQQ------------------------QQ--QQKAQQQNQQY------**

**Hpylori-2018__325995266_revers** **------MAHHE----QQHQV-----QQQQQQANSQ----------HHHHHHAHHHHYYGGEHHH-HNAQQHAEQQAEQHAQQ------------------------QQ--QQKAQQQNQQY------**

**Hpylori-908__307636682_reverse** **------MAHHE----QQHQV-----QQQQQQANSQ----------HHHHHHAHHHHYYGGEHHH-HNAQQHAEQQAEQHAQQ------------------------QQ--QQKAQQQNQQY------**

**Hpylori-Hp-P-13b__393129874_fo** **------MAHHE----QQHQA-----QQQQQQANSQ----------HHHHHHAHHHHYYGGEHHH-HNAQQHAEQQAEQQAQQQ----------------QQQQAHQQQ--QQKAQQQNQQY------**

**Hpylori-Hp-H-34__393096911_rev** **------MAHHE-------------QQQQQQQANSQ----------HHHHHHAHHHHYYGGEHHH-HNVQQHAEQQAEQHAQQ------------------------QQ--QQQAHQQNQQY------**

**Hpylori-Hp-H-36__393060291_for** **------MAHHE----QQHQA----QQQQQQQANSQ----------HHHHHHAHHHHYYGGEHHH-HNVQQHAEQQAEQHAQQ------------------------QQ--QQQAHQQNQQY------**

**Hpylori-Hp-A-6-HpA__393066921_** **------MAHHE----QQHQA------------------------------------------------------------Q------------------------QQQ--QQKAQQQNQQY------**

**Hpylori-Hp-H-1__471384880_reve** **------MAHHE----QQHQA----QQQQQQQANSQ----------HHHHHHAHHHHYYGGEHHH-HNAQQHAEQQAEQHAQQQ----------------QQQQAHQQQ--QQKAQQQQQQY------**

**Hpylori-Hp-A-8__393067645_reve** **------MAHHE----QQHQA----QQQQQQQANSQ----------HHYHHHAHHHHYYGGEHHH-HNVQQHAEQQAEQHAQQQ----------------QRQQAHQ---------------------**

**Hpylori-Hp-H-41__393058399_rev** **------MAHHE----QQHQA-----QQQQQQANSQ----------HHHHHHVHHHHYYGGEHHH-HNAQQHAEQQAEQHAQQQ----------------QQQQAHQQQ--QQKAQQQNQQY------**

**Hpylori-Hp-H-30__393054214_rev** **------MAHHE----QQHQA------QQQQQANSQ----------HHHHHHAHHHHYYGGEHHH-HNAQQHAEQQAEQHAQQ------------------------QQ--QQKAQQQNQQY------**

**Hpylori-Hp-H-10__393083611_rev** **------MAHHE----QQHQA----QQQQQQQANSQ----------HHHHHHAHHHHYYGGEHHH-HNAQQHAEQQAEQHAQQ------------------------QQ--QQKAQQQNQQY------**

**Hpylori-Hp-P-3__393098685_reve** **------MAHHE----QQHQA-----QQQQQQANSQ----------HHHHHHAHHHHYYGGEHHH-HNAQQHAEQQAEQHAQQ------------------------QQ--QQKAQQQNQQY------**

**Hpylori-Hp-H-19__393090315_rev** **------MAHHE----QQHQA------------------------------------------------------------Q------------------------QQQ--QQKAQQQNQQY------**

**Hpylori-Hp-H-44__393062481_rev** **------MAHHE----QQHQA------------------------------------------------------------Q------------------------QQQ--QQKAQQQNQQY------**

**Hpylori-Hp-H-6__393078985_reve** **------MAHHE----QQHQA----QQQQQQQANSQ-----------HHHHHTHHHHYYGGEHHH-HNAQQHAEQQAEQHAQQQ----------------QQQQAHQQQ--QQKAQQQNQQY------**

**Hpylori-Hp-H-42__393059666_for** **------MAHHE----QQHQA----QQQQQQQANSQ----------HHHHHHAHHHHYYGGEHHH-HNAQQHAEQQAEQHAQQQ-----------------------QQ--QQKAQQQNQQY------**

**Hpylori-Hp-H-4__393077812_reve** **------MAHHE----QQHQAQQQQQQQQQQQANSQ----------HHHHHHAHHHHYYGGEHHH-HNAQQHAEQQAEQHVQQ-----------------QAHQ---QQ--QQKAQQQNQQY------**

**Hpylori-PeCan18__384561342_for** **------MAHHE----QQHQA----QQQQQQQANSQ----------HHHHHHAHHHHYYGGEHHH-HNAQQHAEQQAEQHAQQQ----------------QQQQAHQQQ--QQKAQQQNQQY------**

**Hpylori-NQ1671__327407074_forw** **------MAHHE----QQHQA----QQQQQQQANSQ----------HHHHHHAHHHHYYGGEHHH-HNAQQHAEQQAEQHAQQ------------------------QQ--QQKAQQQNQQY------**

**Hpylori-NQ4191__327407129_reve** **------MAHHE----QQHQA----QQQQQQQANSQ----------HHHHHHAHHHHYYGGEHHH-HNAQQHAEQQAEQHAQQ------------------------QQ--QQKAQQQNQQY------**

**Hpylori-NQ4228__393029926_reve** **------MAHHE----------------QQQQANSQ----------HHHHHHAHHHHYYGGEHHH-HNAQQQAEQQAEQQAQQ------------------------QQ--QQKAQQQNQQY------**

**Hpylori-NQ4060__327406737_forw** **------MAHHE---------------QQQQQANSQ----------HHHHHHAHHHHYYGGEHHH-HNAQQHAEQQAEQQAQQQ----------------QQQQAHQQQ--QQKAQQQNQQY------**

**Hpylori-NQ4200__393025980_reve** **------MAHHE----QQHQA----QQQQQQQANSQ----------HHHHHHAHHHHYYGGEHHH-HNAQQHAEQQAEQQAQQ------------------------QN--QQKAQQQNQQY------**

**Hpylori-ELS37__380873809_rever** **------MAHHE-------------QQQQQQQANSQ----------HHHHHHAHHHHYYGGEHHH-HNAQQHAEQQAEQQAQQQ-----------------------QQ--QQKAQQQNQQY------**

**Hpylori-UM111__529185287_rever** **------MAHHE----------------QQQQANSQ----------HHHHHHAHHHHYYGGEHHH-HNAQQHAEQQAEQQAQQQ----------------QQQKAQQQQ--QQQAQQQNQQY------**

**Hpylori-D33__657213133_reverse** **------MAHHE----------------QQQQANSQ----------HHHHHHEHHHHYYGGEHHH-HNAQQHAEQQAEQQAQQQ----------------QQQKAQQQQ--QQKAQQQNQQY------**

**Hpylori-wls-5-14__594649680_fo** **------MAHHE-----------------QQQANSQ----------HHHHHHAHHHHYYGGEHHH-HNAQQQAEQQAEQQAEQQ----------------AQQQAQQQQ--QQKAQQQNQQY------**

**Hpylori-wls-5-1__594682035_for** **------MAHHE----------------QQQQANSQ----------HHHHHHEHHHHYYGGEHHH-HNAQQQAEQQAEQQAQQQ----------------QQQQAHQQQ--QQKAQQQNQQY------**

**Hpylori-OK310__459943864_forwa** **------MAHHE--------------QQQQQQANSQ----------HHHHHHAHHHHYYGGEHHH-HNVQQHAEQQAEQQA--------------------------QQ--QQKAQQQNQQY------**

**Hpylori-OK113__459942343_forwa** **------MAHHE--------------QQQQQQANSQ----------HHHHHHAHHHHYYGGEHHH-HNAQQHAEQQAEQQAQQ------------------------QQ--QQKAQQQNQQY------**

**Hpylori-F30__317178309_forward** **------MAHHE---------------QQQQQANSQ----------HHHHHHAHHHHYYGGEHHH-HNVQQHAEQQAEQQAQQQ----------------AQQQAHQQQ--QQKAQQQNQQY------**

**Hpylori-Shi417__384556702_forw** **------MAHQQ-------QA----QQQQQQQANSH----------HHHHHHAHHHHYYGGEHHH-HNAQQHAEQQAEQQAQQQ----------------QQQQAHQQQ--QQKAQQQNQQY------**

**Hpylori-Puno135__344335753_for** **------MAH------------------QQQQANSQ----------HHHHHHAHHHHYYGGEHHH-HNAQQHAEQQAEQQVQQQ--------------------AQQQQ--QQQAQQQNQQY------**

**Hpylori-Aklavik117__425626953_** **------MAHHE----------------QQQQANSQ----------HHHHHHAHHHHYYGGEHHH-HNAQQHAEQQAEQKAQQQ----------------QQQKAQQ---------------------**

**Hpylori-PeCan4__308064373_forw** **------MAHHE-------------QQQQQQQANSQ----------HHHHHHAHHHHYYGGEHHH-HNAQQHAEQQAEQQAQQQ----------------QQQQAHQQQ--QQKAQQQNQQY------**

**Hpylori-UM114__529216043_forwa** **------MAHHE----QQHQA----QQQQQQQANSQ-----------HHHHHVHHHHYYGGEHHH-HNAQQHAEQQAEQQAHQ------------------------QQ--QQKAQQQNQQY------**

**Hpylori-UM054__507146682_forwa** **------MAHHE----QQHQA-----QQQQQQANSQ----------HHHHHHAHHHHYYGGEHHH-HNAEQHAEQQAEQQAQQQ----------------QQQQAHQQQ--QQKAQQQNQQY------**

**Hpylori-UM018__507146652_rever** **------MAHHE----QQHQA-----QQQQQQANSQ----------HHHHHHVHHHHYYGGEHHH-HNAQQHAEQQAEQQAQQQ----------------QQQQAHQQQ--QQKAQQQNQQY------**

**Hpylori-India7__317008680_reve** **------MAHHE----QQHQA----QQQQQQQANSQ----------HHHHHHAHHHHYYGGEHHH-HNAQQHAEQQAEQQAQQQ-----------------------QQ--QQKAQQQNQQY------**

**Hpylori-BM012A__558757555_reve** **------MAHHE--------------QQQQQQANSQ----------HHHHHHAHHHHYYGGEHHH-HNAQQHAEQQAEQQAQQQ-----------------------QQ--QQKAQQQNQQY------**

**Hpylori-Hp-H-28__393051219_for** **------MAHHE--------------QQQQQQANSQ----------HHHHHHAHHHHYYGGEHHH-HNAQQHAEQQAEQQAQQ------------------------QQ--QQKAQQQNQQY------**

**Hpylori-X47-2AL__558552313_rev** **------MAHHE--------------QQQQQQANSQ----------HHHHHHAHHHHYYGGEHHH-HNAQQHAEQQAEQQAQQQ----------------QQQQAQQQQ--QQKAQQQNQQY------**

**Hpylori-Lithuania75__317011857** **------MAHHE-------------QQQQQQQANSQ----------HHHHHHAHHHHYYGGEHHH-HNAEQHAEQQAEQQAEQQAQQHAEQQ--------AEQQAQQQQ--QQKAQQQNQQY------**

**Hpylori-26695a__409893163_forw** **------MAHHE--------------QQQQQQANSQ----------HHHHHHAHHHHYYGGEHHH-HNAQQHAEQQAEQQAQQHX---------------QQQQAHQQQ--QQKAQQQNQQY------**

**Hpylori-P12__210132169_forward** **------MAHHE----QQHQA----QQQQQQQANSQ----------HHHHHHEHHHHYYGGEHHH-HNAQQHAEQQAEQQAQQQ----------------QQQQAHQQQ--QQKVQQQNQQY------**

**Hpylori-G27__208431905_forward** **------MAHHE-------------QQQQQQQANSQ----------HHHHHHAHHHHYYGGEHHH-HNAQQHAEQQAEQQAQQQ----------------QQQQAHQQQ--QQKAQQQNQQY------**

**Hpylori-B128__216946188_forwar** **------MAHHE---------------QQQQQANSQ----------HHHHHHAHHHHYYGGEHHH-HNAQQHAEQQAEQQAQQQ---------------------QQQQ--QQQAQQQNQQY------**

**Hpylori-B8__298354685_reverse_** **------MAHHE---------------QQQQQANSQ----------HHHHHHAHHHHYYGGEHHH-HNAQQHAEQQAEQQAQQQ---------------------QQQQ--QQQAQQQNQQY------**

**Hpylori-J166output-1moA__63328** **------MAHHE---------------QQQQQANSQ----------HHHHHHAHHHHYYGGEHHH-HNAQQHAEQQAEQQAQQQ---------------------QQQQ--QQQAQQQNQQY------**

**Hpylori-Hp-P-23__393110378_for** **------MAHHE----QQHQA-----QQQQQQANSQ----------HHHHHHAHHHHYYGGEHHH-HNAQQQAEQQAEQQAEQQAQQQ------------QQQQAHQQQ--QQKAQQQNQQY------**

**Hpylori-UM045__507146963_forwa** **------MAHHE----QQHQA-----QQQQQQANSQ----------HHHHHHAHHHHYYGGEHHH-HNAQQHAEQQAEQQAQQQ----------------QQQQAHQQQ--QQKAQQQNQQY------**

**Hpylori-NQ4076__393031057_forw** **------MAHHE--------------QQQQQQANSQ----------HHHHHHAHHHHYYGGEHHH-HNAQQHAEQQAEQQAQQQ----------------QQQQAHQQQ--QQKAQQQNQQY------**

**Hpylori-NQ4099__393029120_reve** **------MAHHE----QQHQV----QQQQQQQANSQ----------HHHHHHAHHHHYYGGEHHH-HNAQQHAEQQAEQHAEQQAEQQAQQQQQQQAQQQQQQQAQQQN--QQKAQQQNQQY------**

**Hpylori-NQ4044__393030900_forw** **------MAHHE----QQHQA----QQQQQQQANSQ----------HHHHHHAHHHHYYGGEHHH-HNAQQHAEQQAEQQAQQQ----------------QQQQAHQQQ--QQKAQQQNQQY------**

**Hpylori-SJM180__308059716_forw** **------MAHHE----QQHQA----QQQQQQQANSQ----------HHHHHHAHHHHYYGGEHHH-HNAQQHAEQQAEQQAQQQ----------------QQQQAHQQQ--QQKAQQQNQQY------**

**Hpylori-SouthAfrica20__5321058** **------MAHHE----QQ--------QQAQQQANSQ----------HHHHHHAHHHHYYGGEHHH-HNAQQYAEQQAEQQAQQQ----------------QQQQAHQQQ--QQKAQQQNQQY------**

**Hpylori-SouthAfrica50__5310740** **------MAHHE------------QQQQAQQQANSQ----------HHHHHHAHHHHYYGGEHHH-HNAQQHAEQQAEQQAQQ------------------------QQ--QQKAQQQNQQY------**

**Hpylori-SouthAfrica7__31701028** **------MAHHE-------------QQQAQQQANSQ----------HHHHHHAHHHHYYGGEHHH-HNAQQYAEQQAEQQAEQQ----------------AQQ---QQQ--QQKAQQQNQQY------**

**Hacinonychis-str.-Sheeba__1097** **------MANHEHNQQNQQQAQ--ACQQNQQQANGQ------------HHHHEHHHHYYGGTHHH-H---HHAEQHAEQQAN-----------------------------QQARQQQNQQS------**

**Hpylori-N6__384527583_reverse_** **------MAHHE----QQHQA----QQQQQQQANSQ----------HHHHHHEHHHHYYGGEHHH-HNAQQHAEQQAEQQAQQQ----------------QQQQAHQQQ--QQKAQQQNQQY------**

**Hpylori-GAM71Ai__459544270_rev** **------MAHHE----------------EQHGGHHH------------HHHHTHHHHYHGGEHHH-HHHSSHHEE-------------------------GCCSASDSH--HQEEGCCHGHHE-----**

**Hpylori-GAM201Ai__459498646_fo** **------MAHHE----------------EQHGGHHH------------HHHHTHHHHYHGGEHHH-HHHSSHHEE-------------------------GCCSTSDSH--HQEEGCCHGHHE-----**

**Hpylori-GAM117Ai__535930598_fo** **------MAHHE----------------EQHGGHHH------------HHHHTHHHHYHGGEHHH-HHHSSHHEE-------------------------GCCSTSDSH--HQEEGCCHGHHE-----**

**Hpylori-GAM118Bi__459498783_re** **------MAHHE----------------EQHGGHHH------------HHHHTHHHHYHGGEHHH-HHHSSHHEE-------------------------GCCSTSDSH--HQEEGCCHGHHE-----**

**Hpylori-GAM270ASi__459540604_f** **-----------------------------------------------HHHHTHHHHYHGGEHHH-HHHSSHHEE-------------------------GCCSTSDSH--HQEEGCCHGHHE-----**

**Hpylori-GAM114Ai__459493577_fo** **------MAHHE----------------EQHGGHHH------------HHHHTHHHHYHGGEHHH-HHHSSHHEE-------------------------GCCSTSDSH--HQEEGCCHGHHE-----**

**Hpylori-Hp-P-8__393100538_reve** **------MAHHE----------------EQHGGHHH------------HHHHTHHHHYHGGEHHH-HHHSSHHEE-------------------------GCCSTSDSH--HQEEGCCHGHHE-----**

**Hpylori-Gambia94-24__317013424** **------MAHHE----------------EQHGGHHH------------HHHHTHHHHYHGGEHHH-HHHSSHHEE-------------------------GCCSTSDSH--HQEEGCCHGHHE-----**

**Hpylori-Hp-H-21__393089925_for** **------MAHHE----------------EQHGGHHH------------HHHHTHHHHYHGGEHHH-HHHSSHHEE-------------------------GCCSTSDSH--HQEEGCCHGHHE-----**

**Hpylori-GAM250T__459523467_rev** **------MAHHE----------------EQHGGHHH------------HHHHTHHHHYHGGEHHH-HHHSSHHEE-------------------------GCCSTSDSH--HQEEGCCHGHHE-----**

**Hpylori-Hp-A-16__393072533_rev** **------MAHHE----------------EQHGGHHH------------HHHHTHHHHYHGGEHHH-HHHSSHHEE-------------------------GCCSTSDSH--HQEECCCHGHHE-----**

**Hpylori-GAM210Bi__459506981_re** **------MAHHE----------------EQHGGHHH------------HHHHTHHHHYHGGEHHH-HHHSSHHEE-------------------------GCCSTSDSH--HQEEGCCHGHHE-----**

**Hpylori-J99__12057207_reverse_** **------MAHHE----------------EQHGGHHH------------HHHHTHHHHYHGGEHHH-HHHSSHHEE-------------------------GCCSTSDSH--HQEEGCCHGHHE-----**

**Hpylori-Hp-M6__393145710_rever** **------MAHHE----------------EQHGG-HH------------HHHHTHHHHYHGGEHHH-HHHSSHHEE-------------------------GCCSTSDSH--HQEEGCCHGHHE-----**

**Hpylori-Hp-A-20__393045038_rev** **--------------------------------------------------------------------SSHHEE-------------------------GCCSTSDSH--HQEEGCCHGHHE-----**

**Hpylori-2017__325996870_revers** **------MAHHE----------------EQHGGHHH------------HHHHTHHHHYHGGEHHH-HHHSSHHEE-------------------------GCCSTSDSH--HQEEGCCHGHHE-----**

**Hpylori-2018__325995266_revers** **------MAHHE----------------EQHGGHHH------------HHHHTHHHHYHGGEHHH-HHHSSHHEE-------------------------GCCSTSDSH--HQEEGCCHGHHE-----**

**Hpylori-908__307636682_reverse** **------MAHHE----------------EQHGGHHH------------HHHHTHHHHYHGGEHHH-HHHSSHHEE-------------------------GCCSTSDSH--HQEEGCCHGHHE-----**

**Hpylori-Hp-P-13b__393129874_fo** **------MAHHE----------------EQHGGHHH------------HHHHTHHHHYHGGEHHH-HHHSSHHEE-------------------------GCCSTSDSH--HQEEGCCHGHHE-----**

**Hpylori-Hp-H-34__393096911_rev** **------MAHHE----------------EQHGGHHH------------HHHHTHHHHYHGGEHHH-HHHSSHHEE-------------------------GCCSTSDSH--HQEEGCCHGHHE-----**

**Hpylori-Hp-H-36__393060291_for** **------MAHHE----------------EQHGGHHH------------HHHHTHHHHYHGGEHHH-HHHSSHHEE-------------------------GCCSTSDSH--HQEEGCCHGHHE-----**

**Hpylori-Hp-A-6__393066921_reve** **------MAHHE----------------EQHGGHHH------------HHHHTHHHHYHGGEHHH-HHHSSHHEE-------------------------GCCSTSDSH--HQEEGCCHGHHE-----**

**Hpylori-Hp-H-1__471384880_reve** **------MAHHE----------------EQHGGHHH------------HHHHTHHHHYHGGEHHH-HHHSSHHEE-------------------------GCCSTSDSH--HQEEGCCHGHHE-----**

**Hpylori-Hp-A-8__393067645_reve** **------MAHHE----------------EQHGGHHH------------HHHHTHHHHYHGGEHHH-HHHSSHHEE-------------------------GCCSTSDSH--HQEEGCCHGHHE-----**

**Hpylori-Hp-H-41__393058399_rev** **------MAHHE----------------EQHGGHHH------------HHHHTHHHHYHGGEHHH-HHHSSHHEE-------------------------GCCCTSDSH--HQEEGCCHGHHE-----**

**Hpylori-Hp-H-30__393054214_rev** **------MAHHE----------------EQHGGHHH------------HHHHTHHHHYHGGEHHH-HHHSSHHEE-------------------------GCCSTSDSH--HQEEGCCHGHHE-----**

**Hpylori-Hp-H-10__393083611_rev** **------MAHHE----------------EQHGGHHH------------HHHHTHHHHYHGGEHHH-HHHSSHHEE-------------------------GCCSTSDSH--HQEEGCCHGHHE-----**

**Hpylori-Hp-H-5b__393125211_rev** **------MAHHE----------------EQHGGHHH------------HHHHTHHHHYHGGEHHH-HHHSSHHEE-------------------------GCCSTSDSH--HQEEGCCHGHHE-----**

**Hpylori-Hp-P-3__393098685_reve** **------MAHHE----------------EQHGGHHH------------HHHHTHHHHYHGGEHHH-HHHSSHHEEGCCSTSDSHHQEX-----------XGCCSTSDSH--HQEEGCCHGHHE-----**

**Hpylori-Hp-H-19__393090315_rev** **------MAHHE----------------EQHGGHHH------------HHHHTHHHHYHGGEHHH-HHHSSHHEE-------------------------GCCSTSDSH--HQEEGCCHGHHE-----**

**Hpylori-Hp-H-44__393062481_rev** **------MAHHE----------------EQHGGHHH------------HHHHTHHHHYHGGEHHH-HHHSSHHEE-------------------------GCCSTSDSH--HQEEGCCHGHHE-----**

**Hpylori-Hp-H-6__393078985_reve** **------MAHHE----------------EQHGGHHH------------HHHHTHHHHYHGGEHHH-HHHSSHHEE-------------------------GCCSTSDSH--HQEEGCCHGHHE-----**

**Hpylori-Hp-H-42__393059666_for** **------MAHHE----------------EQHGGHHH------------HHHHTHHHHYHGGEHHH-HHHSSHHEE-------------------------GCCSTSDSH--HQEEGCCHGHHE-----**

**Hpylori-Hp-H-4__393077812_reve** **------MAHHE----------------EQHGGHHH------------HHHHTHHHHYHGGEHHH-HHHNSHHEE-------------------------GCCSTSDSH--HQEEGCCHGHHE-----**

**Hpylori-PeCan18__384561342_for** **------MAHHE----------------EQHGGHHH------------HHHHTHHHHYHGGEHHH-HHHSSHHEE-------------------------GCCSTSDSH--HQEEGCCHGHHE-----**

**Hpylori-NQ1671__327407074_forw** **------MAHHE----------------EQHGGHHH------------HHHHTHHHHYHGGEHHH-HHHSSHHEE-------------------------GCCSTSDSH--HQEEGCCHGHHE-----**

**Hpylori-NQ4191__327407129_reve** **------MAHHE----------------EQHGGHHH------------HHHHTHHHHYHGGEHHH-HHHSSHHEE-------------------------GCCSTSDSH--HQEEGCCHGHHE-----**

**Hpylori-NQ4228__393029926_reve** **------MAHHE----------------EQHGGHHH------------HHHHTHHHHYHGGEHHH-HHHSSHHEE-------------------------GCCSTSDSH--HQEEGCCHGHHE-----**

**Hpylori-NQ4060__327406737_forw** **------MAHHE----------------EQHGGHHH------------HHHHTHHHHYHGGEHHH-HHHSSHHEE-------------------------GCCSTSDSH--HQEEGCCHGHHE-----**

**Hpylori-NQ4200__393025980_forw** **------MAHHE----------------EQHGGHHH------------HHHHTHHHHYHGGEHHH-HHHSSHHEE-------------------------GCCSTSDSH--HQEEGCCHGHHE-----**

**Hpylori-ELS37__380873809_rever** **------MAHHE----------------EQHGGHHH------------HHHHTHHHHYHGGEHHH-HHHSSHHEE-------------------------GCCSTSDSH--HQEEGCCHGHHE-----**

**Hpylori-UM111__529185287_forwa** **------MAHHE----------------EQHGGHHH------------HHHHTHHHHYHGGEHHH-HHHSSHHEE-------------------------GCCSSSDSH--HQEEGCCHGHHE-----**

**Hpylori-D33__657213133_forward** **------MAHHE----------------EQHGGHHH------------HHHHTHHHHYHGGEHHH-HHHSSHHEE-------------------------GCCSTSDSH--HQEEGCCHGHHE-----**

**Hpylori-wls-5-14__594649680_re** **------MAHHE----------------EQHGGHHH------------HHHHTHHHHYHGGEHHH-HHHSSHHEE-------------------------GCCSTSDSH--HQEEGCCHGHHE-----**

**Hpylori-wls-5-1__594682035_rev** **------MAHHE----------------EQHGGHHH------------HHHHTHHHHYHGGEHHH-HHHSSHHEE-------------------------GCCSTSDSH--HQEEGCCHGHHE-----**

**Hpylori-OK310__459943864_rever** **------MAHHE----------------EQHGGHHH------------HHHHTHHHHYHGGEHHH-HHHSSHHEE-------------------------GCCSTSDSH--HQEEGCCHGHHE-----**

**Hpylori-OK113__459942343_rever** **------MAHHE----------------EQHGGHHH------------HHHHTHHHHYHGGEHHH-HHHSSHHEE-------------------------GCCSTSDSH--HQEEGCCHGHHE-----**

**Hpylori-F30__317178309_reverse** **------MAHHE----------------EQHGGHHH------------HHHHTHHHHYHGGEHHH-HHHSSHHEE-------------------------GCCSTSDSH--HQEEGCCHGHHE-----**

**Hpylori-Shi417__384556702_reve** **------MAHHE----------------EQHG-EHH------------HHHHTHHHHYHGGEHHH-HHHSSHHEE-------------------------GCCSTSDSH--HQEEGCCHGHHE-----**

**Hpylori-Puno135__344335753_rev** **------MAHHE----------------EQHGGHHH------------HHHHTHHHHYHGGEHHH-HHHSSHHEE-------------------------GCCSTSDSH--HQEEGCCHGHHE-----**

**Hpylori-Aklavik117__425626953_** **------MAHHE----------------EQHGGHHH------------HHHHTHHHHYHGGEHHH-HHHSSHHEE-------------------------GCCSASDSH--HQEEGCCHGHHE-----**

**Hpylori-PeCan4__308064373_reve** **------MAHHE----------------EQHGGHHH------------HHHHTHHHHYHGGEHHH-HHHSSHHEE-------------------------GCCSTSDSH--HQEEGCCHGHHE-----**

**Hpylori-UM114__529216043_rever** **------MAHHE----------------EQHGGHHH------------HHHHTHHHHYHGGEHHH-HHHSSHHEE-------------------------GCCSTSDSH--HQEEGCCHGHHE-----**

**Hpylori-UM054__507146682_rever** **------MAHHE----------------EQHGGHHH------------HHHHTHHHHYHGGEHHH-HHHSSHHEE-------------------------GCCSTSDSH--HQEEGCCHGHHE-----**

**Hpylori-UM018__507146652_forwa** **------MAHHE----------------EQHGGHHH------------HHHHTHHHHYHGGEHHH-HHHSSHHEE-------------------------GCCSTSDSH--HQEEGCCHGHHE-----**

**Hpylori-India7__317008680_forw** **------MAHHE----------------EQHGGHHH------------HHHHTHHHHYHGGEHHH-HHHSSHHEE-------------------------GCCSTSDSH--HQEEGCCHGHHE-----**

**Hpylori-BM012A__558757555_forw** **------MAHHE----------------EQHGGHHH------------HHHHTHHHHYHGGEHHH-HHHSSHHEE-------------------------GCCSTSDSH--HQEECCCHGHHE-----**

**Hpylori-Hp-H-28__393051219_rev** **------MAHHE----------------EQHGGHHH------------HHHHTHHHHYHGGEHHH-HHHSSHHEE-------------------------GCCSTSDSH--HQEEGCCHGHHE-----**

**Hpylori-X47-2AL__558552313_for** **------MAHHE----------------EQHGGHHH------------HHHHTHHHHYHGGEHHH-HHHSSHHEE-------------------------GCCSTSDSH--HQEEGCCHGHHE-----**

**Hpylori-Lithuania75__317011857** **------MAHHE----------------EQHGGHHH------------HHHHTHHHHYHGGEHHH-HHHSSHHEE-------------------------GCCSTSDSH--HQEEGCCHGHHE-----**

**Hpylori-26695-a__409893163_rev** **------MAHHE----------------EQHGGHHH------------HHHHTHHHHYHGGEHHH-HHHSSHHEE-------------------------GCCSTSDSH--HQEEGCCHGHHE-----**

**Hpylori-N6__384527583_forward_** **------MAHHE----------------EQHGGHHH------------HHHHTHHHHYHGGEHHH-HHHSSHHEE-------------------------GCCSTSDSH--HQEEGCCHGHHE-----**

**Hpylori-P12__210132169_reverse** **------MAHHE----------------EQHGGEHH------------HHHHTHHHHYHGGEHHH-HHHSSHHEE-------------------------GCCSTSDSH--HQEEGCCHGHHE-----**

**Hpylori-G27__208431905_reverse** **------MAHHE----------------EQHGGHHH------------HHHHTHHHHYHGGEHHH-HHHSSHHEE-------------------------GCCSASDSH--HQEEGCCHGHHE-----**

**Hpylori-B128__216946188_revers** **------MAHHE----------------EQHGGHHH------------HHHHTHHHHYHGGEHHH-HHHSSHHEE-------------------------GCCSTSDSH--HQEEGCCHGHHE-----**

**Hpylori-B8__298354685_forward_** **------MAHHE----------------EQHGGHHH------------HHHHTHHHHYHGGEHHH-HHHSSHHEE-------------------------GCCSTSDSH--HQEEGCCHGHHE-----**

**Hpylori-J166output-1moA__63328** **------MAHHE----------------EQHGGHHH------------HHHHTHHHHYHGGEHHH-HHHSSHHEE-------------------------GCCCTSDSH--HQEEGCCHGHHE-----**

**Hpylori-Hp-P-23__393110378_rev** **------MAHHE----------------EQHGGHHH------------HHHHTHHHHYHGGEHHH-HHHSSHHEE-------------------------GCCSTSDSH--HQEEGCCHGHHE-----**

**Hpylori-UM045__507146963_rever** **------MAHHE----------------EQHGGHHH------------HHHHTHHHHYHGGEHHH-HHHSSHHEE-------------------------GCCSTSDSH--HQEEGCCHGHHE-----**

**Hpylori-NQ4076__393031057_reve** **------MAHHE----------------EQHGGHHH------------HHHHTHHHHYHGGEHHH-HHHSSHHEE-------------------------GCCSTSDSH--HQEEGCCHGHHE-----**

**Hpylori-NQ4099__393029120_forw** **------MAHHE----------------EQHGGHHH------------HHHHTHHHHYHGGEHHHHHHHSSHHEE-------------------------GCCSTSDSH--HQEEGCCHGHHE-----**

**Hpylori-NQ4044__393030900_reve** **------MAHHE----------------EQHGGHHH------------HHHHTHHHHYHGGEHHH-HHHSSHHEE-------------------------GCCSTSDSH--HQEEGCCHGHHE-----**

**Hpylori-SJM180__308059716_reve** **------MAHHE----------------EQHGGHHH------------HHHHTHHHHYHGGEHHH-HHHSSHHEE-------------------------GCCSTSDSH--HQEEGCCHGHHE-----**

**Hpylori-SouthAfrica20__5321058** **------MAHHE----------------EQHGGHHH------------HHHHTHHHHYHGGEHHH-HHHSSHHEE-------------------------GCCSSSNSH--HQEEGCCHGHHE-----**

**Hpylori-SouthAfrica50__5310740** **------MAHHE----------------EQHGGHHH------------HHHHTHHHHYHGGEHHH-HHHSSHHEE-------------------------GCCSSSNSH--HQEEGCCHGHHE-----**

**Hpylori-SouthAfrica7__31701028** **------MAHHE----------------EQHGGHHH------------HHHHTHHHHYHGGEHHH-HHHSSHHEE-------------------------GCCSSSNSH--HQEEGCCHGHHE-----**

**Hacinonychis-str.-Sheeba__1097** **------MAHHE----------------EKHGEHH-------------HHHHTHHHHYHGGEHHHHHHHGSHHEE-------------------------GCCNTHHEQ--H-EDGCCHGHHE-----**

**Hcetorum-MIT-00-7128__38455496** **------MAHH-----------------EHHGEHHH------------HHQHHHHHHYHGGEHHH-HHHHYSGGE-------------------------------EHHGEHHEEGCCHGHHGHHEED**

**Hcetorum-MIT-99-5656__38455327** **------MSGHK----------------EHHGEHHH------------HHSHTHHHHYHGGEHHH-HHHSSHHEE-------------------------NCCHDGKHHGEHHEEGCCHGHHGHHE--**

**Hsuis-HS5__321146766_forward_2** **------MSHHT----------------DECCHEHE--------ECAEHHHHHHHHHYYGGHHHH-HHH--HH---------------------------G-----EHH--HH-----HEHEEEHN--**

**Hsuis-HS1__321148481_forward_3** **------MSHHT----------------DECCHEHE--------ECAEHHHHHHHHHYYGGHHHH-HHH--HH---------------------------G-----EHH--HH-----HEHEE--EHN**

**Hheilmannii-ASB1.4__408906187_** **------MA-------------------DECCHDHNEGGCCHDHEGHGDHHHHHHHHYYGGHHHH-HHH--HH---------------------------GG--------------------------**

**Hbizzozeronii-CCUG-35545__3753** **------MSGHS----------------EECCHGHE-------NECSE-HHHHHHHHYHGGTHHH-HHH--HHGE-------------------------------HHH--HHHDGDHHHHEG-----**

**Hbizzozeronii-CIII-1__33533225** **------MSGHS----------------EECCHGHE-------NECSE-HHHHHHHHYHGGTHHH-HHH--HHGE-------------------------------HHH--HHHDGDHHHHEG-----**

**Hfelis-ATCC-49179__326319845_f** **------MSEHG-----------------ECCHGHE------GHEGHG-VHHHHHHHYHGGTHHH-HHH--HH---------------------------G--GEHHHH-HHNQHGEHGSN-------**

**Hmustelae-12198__290963437_rev** **MNFSTNGKHNG-----------------EHHHG-EHHH----------GEHHHGEHHHGEHHHGEHHHGEHHH--------------------------GEHHHGEHHHGEHHHGEHHHGK------**
