# Supplementary material for: Evolution of Helicobacter: Acquisition by Gastric Species of Two Histidine-Rich Proteins Essential for Colonization
Source: PLoS Pathog. 2015 Dec 7;11(12):e1005312. doi: 10.1371/journal.ppat.1005312 (PMC4671568; doi:10.1371/journal.ppat.1005312)
Supplement: S3 Table — (PDF) [file ppat.1005312.s007.pdf]

Suppl TABLE S3

Primers used in this study

Primer	Position of the primer	Sequence (5'-3')
**Construction of the *Δhpn::cm* mutant**		
H941	HP1427_Fwd	GAATGAAGTGAGGAAATGGCC
H942	HP1427_Cm_Rev	CGGATCTGTTGAAGGGCGGGATGACTCCTTTGATTAAAAA
H943	HP1427_Cm_Fwd	GCGTAAGGAGGAATAATGTATCGGTGTGGCTAGGGGCAA
H944	HP1427_Rev	CCCGCTATAGCCAAGACCGGC
**Construction of the Δ*hpn::km* mutant**		
H1081	HP1427_Fwd	GATTTGAACGATAGCCTAGAC
H1082	HP1427_Km_Rev	GTTAGTCACCCGGGTACGATGACTCCTTTGATTAAAAA
H1083	HP1427_Km_Fwd	TACCTGGAGGGAATAATGTATCGGTGTGGCTAGGGGCAA
H1084	HP1427_Rev	CCCGCTATAGCCAAGACCGGC
**Construction of the *Δhpn-2::km* mutant**		
H949	HP1432_Fwd	GCACAAATGGCTGTCTATCTC
H950	HP1432_Km_Rev	GTTAGTCACCCGGGTACGATGACTCCTTTAATTGAAAT
H951	HP1432_Km_Fwd	TACCTGGAGGGAATAATGTTGGGGCGTTTGTGGGGGCGG
H952	HP1432_Rev	ACCAACGCCCCAAAAGTCAAGGACAAACCC
**Construction of *hpn* and *hpn-2* expression plasmids in the pRSFDuet1 vector**		
pRSF-HpnUP	*hpn* gene	AAACCGGCCATGGCACACCATGAAGAACAAC
pRSF-HpnDO	*hpn* gene	TTTTAAGCTTTTACTCGTGATGCCCGTGGC
pRSF-Hpn-2UP	*hpn-2* gene	AAACCGGCATATGGCACACCATGAACAACAAC
pRSF-Hpn-2DO	*hpn-2* gene	TTTTGGTACCTCAATATTGTTGGTTTTG
**Construction of the recipient strain for recomplementation**		
PUreIUP	*ureI* promoter	CTGCAGGGCTTTTTTTGTTTTTATTTTTTGTC
PUreIDO	*ureI* promoter	GGATCCGATATCCATATGTTTCCTTCCAAACAAAAATTTTTACAAC
**Construction of the *hpn* complemented strain**		
hpnUP	*hpn* gene	AAAGATATCGCTTAGAGAGCGCTAGAATG
hpnDO	*hpn* gene	GGATCCTTTGATCAGAATGGGTTAGAATGG
**Construction of the *hpn-2* complemented strain**		
Hpn-2UP	*hpn-2* gene	GATATCATTGCTATCTATCTCATAAGTC
Hpn-2DO	*hpn-2* gene	GGATCCAAGGTGATTTGCCCCATATCGC

**Constructions for the bacterial two hybrid screen (UP: upstream primer, DO: downstream primer as a function of the amplified gene)**	
Cloning of *hpn*, *hpnΔCter*, *hpn-2* and *hpn-2ΔCter* into pUT18 and pNKT25	
HpnUT18UP	CTGCAGCAGTGAGCTAGAATTTAAATTC
HpnUT18DO	GAATTCGACTCGTGATGCCCGTGGCAAC
HpnΔcterUT18DO	GAATTCGAATGATGAGAGCTGTGGTGGTGATG
Hpn-2UT18UP	CTGCAGAGCGCTCAAATTGGTGCTG
Hpn-2UT18DO	GAATTCGAATATTGTTGGTTTTGTTGTTGTGC
Hpn-2ΔcterUT18DO	GAATTCGACGCATTATGGTGATGGTGTTCGCC
Cloning of *hpn*, *hpnΔCter*, *hpn-2* and *hpn-2ΔC* ter into pKT25 and pUT18C	
HpnKT25UP	CTGCAGGGATGGCACACCATGAAGAACAAC
HpnKT25DO	GAATTCTTACTCGTGATGCCCGTGGC
HpnΔcterKT25DO	GAATTCTTAATGATGAGAGCTGTGGTGGTG
Hpn-2KT25UP	CTGCAGGGATGGCACACCATGAACAACAAC
Hpn-2KT25DO	GAATTCTAAAAGCCAGGAGTAGCCC
Hpn-2ΔcterKT25DO	GAATTCTTACGCATTATGGTGATGGTGTTCGCC
Cloning of *ureA* into pKT25	
UreA 719 KT25UP	CACTCTAGATATGAAACTCACCCCAAAAG
UreA 720 KT25DO	CACGGTACCTTACTCCTTAATTGTTTTTAC
Cloning of *ureA* into pKNT25	
UreA 810 T18UP	GCGTCTAGATATGAAACTCACCCCAAAAG
UreA 811 T18DO	ACGGGTACCTTACTCCTTAATTGTTTTAC
Cloning of *ureE* into pUT18 and pKNT25	
UreEUT18UP	CTGCAGCCCTGCTTGGTTACTCTTTATCC

UreEUT18DO	GAATTCGGATTTCATGACCACTTTAAATCGC
Cloning of *ureF* into pUT18 and pKNT25	
UreFUT18UP	CTGCAGATGAAATAGAAAAATAACAAATG
UreFUT18DO	GAATTCGAAGACATATAAAGGCGCGAG
Cloning of *ureG* into pUT18 and pKNT25	
UreGUT18UP	CTGCAGCTCAAATTGAAAGGAATTTTATG
UreGUT18DO	GAATTCGAATCTTCCAATAAAGCGTTGC
Cloning of *ureH* into pUT18 and pKNT25	
UreHUT18UP	CTGCAGAACGCTTTATTGGAAGATTGATG
UreHUT18DO	GAATTCGAAATCTTTTGCGTGATGGTTTG
Cloning of *ureI* into pUT18 and pKNT25	
UreIUT18UP	CTGCAGTTGTTTGGAAGGAAAAGGCAATG
UreIUT18DO	GAATTCGACACCCAGTGTTGGATAAAGAG
Cloning of *hypA* into pUT18 and pKNT25	
HypAUT18UP	CTGCAGTTTAAAGATTAAGGTTTAGTATG
HypAUT18DO	GAATTCGATTCCGCTAACATTTCTAAAG
Cloning of *hypB* into pUT18 and pKNT25	
HypBUT18UP	CTGCAGTTTTAAAAGAAAGGAAAAACATG
HypBUT8DO	GAATTCGAAAACGAATGCGTGGACTGG
