# Supplementary material for: Evolution of Helicobacter: Acquisition by Gastric Species of Two Histidine-Rich Proteins Essential for Colonization
Source: PLoS Pathog. 2015 Dec 7;11(12):e1005312. doi: 10.1371/journal.ppat.1005312 (PMC4671568; doi:10.1371/journal.ppat.1005312)
Supplement: S4 Table — (PDF) [file ppat.1005312.s008.pdf]

SUPPL TABLE S4.

Bacterial and plasmids used in this study.

**Strains**	**Relevant characteristics**	**Reference**
Escherichia coli		
MC1061	*F^- Δ(ara-leu)7697 [araD139]B/r Δ(codB-lacI)3 galK16 galE15 λ- e14- mcrA0 relA1 rpsL150(strR) spoT1 mcrB1 hsdR2(r^- m^+)*	[62]
BTH101	*F^-, cya-99, araD139, galE15, galK16, rpsL1 (Str^R), hsdR2, mcrA1, mcrB1*	[64]
BL21(DE3)	*F^- dcm ompT hsdS(rB^- mB^-) gal λ(DE3)*	Novagen
Helicobacter pylori		
B128	Sequenced parental strain	[27,28]
B128 Δ*hpn Cm*	Cm^R	This work
B128 Δ*hpn Kn*	Km^R	This work
B128 Δ*hpn-2 Kn*	Km^R	This work
B128 Δ*hpn*Δ*hpn-2*	Cm^RKm^R	This work
B128Δ*hpn phpn*	Km^RCm^R	This work
B128Δ*hpn-2 phpn-2*	Km^RCm^R	This work
X47-2AL	Sequenced parental strain	[65]
X47-2AL Δ*hpn Kn*	Kn^R	This work
X47-2AL Δ*hpn-2 Kn*	Km^R	This work
SS1	Sequenced parental strain	[66]
SS1 Δ*hpn Cm*	Cm^R	This work
SS1 Δ*hpn Kn*	Kn^R	This work
SS1 Δ*hpn-2 Kn*	Km^R	This work
SS1 Δ*hpn*Δ*hpn-2*	Cm^RKm^R	This work
SS1Δ*hpn phpn*	Km^RCm^R	This work
SS1Δ*hpn-2 phpn-2*	Km^RCm^R	This work

**Plasmid**	**Relevant characteristics**	**Reference**
Vectors		
pKT25	BACTH vector designed to express a protein fused in frame at its N-terminus with T25 domain of CyaA; p15 ori; Km^R	[64]
pKNT25	BACTH vector designed to express a protein fused in frame at its C-terminus with T25 domain of CyaA; p15 ori; Km^R	[64]
pUT18	BACTH vector designed to express a protein fused in frame at its C-terminus with T18 domain of CyaA; ColE1 ori; Amp^R	[64]
pUT18C	BACTH vector designed to express a protein fused in frame at its N-terminus with T18 domain of CyaA; ColE1 ori; Amp^R	[64]
pKT25-Zip	BACTH control plasmid expressing T25-GCN4 leucine zipper	[64]
pUT18C-Zip	BACTH control plasmid expressing T18-GCN4 leucine zipper	[64]

pKNT25-Zip	BACTH control plasmid expressing GCN4 leucine zipper-T25	[64]
pUT18-Zip	BACTH control plasmid expressing GCN4 leucine zipper-T18	[64]
Plasmids		
*p*RSFDuet1	Cloning vector	Novagen
*p*RSFDuet1-*hpn*	Plasmid for *hpn* overexpression	This work
*p*RSFDuet1-*hpn-2*	Plasmid for *hpn-2* overexpression	This work
pIR203C04	Vector for chromosomal complementation of *H. pylori* mutants	[74]
p(PureI:*hpn*)	Plasmid for chromosomal complementation by *hpn*	This work
p(PureI:*hpn-2*)	Plasmid for chromosomal complementation by *hpn-2*	This work
pUT18(Hpn)<br>pUT18C(Hpn)<br>pKT25(Hpn)<br>pKNT25(Hpn)	Hpn BACTH expression plasmids	This work
pUT18(HpnΔCter)<br>pUT18C(HpnΔCter)<br>pKT25(HpnΔCter)<br>pKNT25(HpnΔCter)	HpnΔCter BACTH expression plasmids	This work
pUT18(Hpn-2)<br>pUT18C(Hpn-2)<br>pKT25(Hpn-2)<br>pKNT25(Hpn-2)	Hpn-2 BACTH expression plasmids	This work
pUT18(Hpn-2ΔCter)<br>pUT18C(Hpn-2ΔCter)<br>pKT25(Hpn-2ΔCter)<br>pKNT25(Hpn-2ΔCter)	Hpn-2ΔCter BACTH expression plasmids	This work
pKT25(UreA)<br>pKNT25(UreA)	UreA BACTH expression plasmids	This work
pUT18(UreE)<br>pKNT25(UreE)	UreE BACTH expression plasmids	This work
pUT18(UreF)<br>pKNT25(UreF)	UreF BACTH expression plasmids	This work
pUT18(UreG)<br>pKNT25(UreG)	UreG BACTH expression plasmids	This work
pUT18(UreH)<br>pKNT25(UreH)	UreH BACTH expression plasmids	This work
pUT18(UreI)<br>pKNT25(UreI)	UreI BACTH expression plasmids	This work
pUT18(HypA)<br>pKNT25(HypA)	HypA BACTH expression plasmids	This work
pUT18(HypB)<br>pKNT25(HypB)	HypB BACTH expression plasmids	This work
